# MACFIV: a novel framework for nonlinear causal inference in the body mass index–hypertension relationship with many weak and pleiotropic genetic instruments

**DOI:** 10.1093/bib/bbaf714

**Published:** 2026-01-11

**Authors:** Dong Chen, Yuquan Wang, Dapeng Shi, Yunlong Cao, Yue-Qing Hu

**Affiliations:** State Key Laboratory of Genetics and Development of Complex Phenotypes, Institute of Biostatistics, School of Life Sciences, Fudan University, 2005 Songhu Road, Yangpu District, Shanghai 200438, China; State Key Laboratory of Genetics and Development of Complex Phenotypes, Institute of Biostatistics, School of Life Sciences, Fudan University, 2005 Songhu Road, Yangpu District, Shanghai 200438, China; Shanghai Center for Mathematical Sciences, Fudan University, 2005 Songhu Road, Yangpu District, Shanghai 200438, China; State Key Laboratory of Genetics and Development of Complex Phenotypes, Institute of Biostatistics, School of Life Sciences, Fudan University, 2005 Songhu Road, Yangpu District, Shanghai 200438, China; State Key Laboratory of Genetics and Development of Complex Phenotypes, Institute of Biostatistics, School of Life Sciences, Fudan University, 2005 Songhu Road, Yangpu District, Shanghai 200438, China; Shanghai Center for Mathematical Sciences, Fudan University, 2005 Songhu Road, Yangpu District, Shanghai 200438, China

**Keywords:** nonlinear causal inference, model average, control function, weak genetic instruments, pleiotropic genetic instruments

## Abstract

Causal inference is an essential approach for understanding biological processes. Traditional causal inference methods assume a linear relationship between different biological traits, whereas their true causal relationship may be nonlinear, such as U-shaped. Moreover, when the instrument set includes weak and pleiotropic genetic instruments, accurately capturing the shape of these relationships becomes challenging. To address these issues, we propose model-averaged control function-based instrumental variable regression, a two-stage framework based on a model-averaged control function approach to estimate the marginal effect function, which represents the derivative of the causal relationship. In the first stage, a model averaging technique is employed to estimate the control function, thereby reducing weak genetic instrument bias. In the second stage, B-spline approximation is applied to estimate the marginal effect function, while SCAD penalization is used to minimize pleiotropic instrument bias. We establish the asymptotic properties of the proposed estimator and demonstrate its robust performance through simulations. Application to the Atherosclerosis Risk in Communities dataset highlights a nonlinear causal relationship between body mass index and hypertension, with the proposed method effectively estimating the specific shape and trend of the relationship.

## Introduction

In recent years, instrumental variable (IV) methods have been widely used in causal inference and Mendelian randomization studies to investigate the causal relationship between two complex traits by using single-nucleotide polymorphisms (SNPs) as instruments. Traditional IV methods, such as two-stage least squares and the limited information maximum likelihood (LIML), are commonly implemented within a two-stage framework [1, 2]. These approaches typically assume linear relationships both between exposure and outcome, as well as between instruments and exposure. However, empirical evidence increasingly suggests the potential for nonlinear relationships among traits, making it essential to extend linear models to nonlinear scenarios. Many studies have already addressed this issue, with some approaches considering nonlinearity between instruments and exposure and relaxing the linear assumption in the first stage of the two-stage framework [3, 4]. Some methods focus on the second stage, relaxing the linearity assumption between exposure and outcome. For example, Terza *et al.* [5] introduced the two-stage residual inclusion method, and Burgess *et al.* [6] proposed a stratified approach to estimate the localized average causal effect, capturing local nonlinearity. Furthermore, recent advancements have simultaneously relaxed linearity assumptions in both stages. For instance, Dai *et al.* [7] and Fan *et al.* [8] explored frameworks that allow for nonlinearities throughout the two-stage process. Overall, most methods aim to achieve accurate nonlinear estimation by strategically relaxing linearity assumptions in one or both stages of the IV framework.

Whether in linear or nonlinear frameworks of causal inference, most existing IV methods impose stringent requirements on the genetic instruments. Typically, instruments must satisfy three key assumptions: (A1: Relevance Restriction) instruments are associated with the exposure; (A2: Exclusion Restriction) instruments have no direct pathway to the outcome; and(A3: Exogenous Restriction) instruments are not related to unobserved confounders conditional on the exposure. A genetic instrument is considered weak if it has a weak association with the exposure, and if it does not satisfy (A2) or (A3), it shows pleiotropy or is regarded as an invalid instrument in IV methods. Most IV methods necessitate prescreening to exclude SNPs that violate these assumptions before proceeding with causal inference. Specifically, when using a large number of SNPs, it often means that many SNPs are weak and incapable of supporting accurate causal inference [9]. A common approach to address the weak instrument problem is to remove all weak instruments. For instance, Guo *et al.* [10] proposed a thresholding strategy to ensure the remaining IVs are sufficiently strong. This thresholding approach has also been extended to nonlinear frameworks to mitigate bias introduced by weak instruments [11]. Conversely, some studies aim to fully utilize all available instrument information, including weak instruments. Fan and Wu [12] developed an IV estimator robust to the existence of both invalid and irrelevant instruments (R2IVE), which divides candidate instruments into subgroups and efficiently incorporates information from weak instruments while controlling for bias. This approach demonstrates that leveraging the complete set of instruments, rather than discarding weaker ones, can yield more robust estimation results, and is also robust in more complex instrument scenarios [13].

In recent studies, model averaging has been recognized as an attractive approach for handling weak instruments, particularly when most or all instruments are relatively weak. Seng and Li [14] proposed a model averaging-based IV method that demonstrated promising results in Mendelian randomization studies [15]. Similarly, in nonlinear settings, Chen *et al.* [16] leveraged model averaging to mitigate bias introduced by weak instruments. Compared to variable selection and regularization approaches, model averaging offers a more robust alternative [17]. It integrates diverse submodels with appropriate weights to reduce the error from model misspecification, with weight estimation often relying on criteria such as Mallows’ criterion and minimization of Kullback–Leibler measures [18–22]. Moreover, it performs well in small-sample scenarios [23]. Its effectiveness in addressing variable selection and weak variable problems has been demonstrated in various statistical applications and Mendelian randomization studies [24–26].

Apart from weak genetic instruments, handling pleiotropy or invalid instruments is another significant challenge in Mendelian randomization. The presence of invalid instruments can lead to inconsistency in traditional estimators [27]. When prior knowledge about instruments is available, Liao [28] and Cheng and Liao [29] demonstrated that shrinkage estimation methods within the generalized method of moments (GMM) framework can identify and exclude invalid instruments. Similarly, Caner *et al.* [30] developed an adaptive Elastic-Net GMM approach under this framework. In the absence of prior knowledge, the sisVIVE method proposed by Kang *et al.* [31] allows for the identification of invalid instruments. Building on this, Windmeijer *et al.* [32] used median estimation and Adaptive Lasso to provide consistent estimation of the set of invalid instruments. Most of these methods rely on the majority rule, which assumes that the number of invalid instruments does not exceed half of the total number of instruments. To relax the majority rule, Guo *et al.* [10] and Windmeijer *et al.* [33] introduced the two-stage hard thresholding and confidence intervals IV procedures, respectively, both of which are based on the plurality rule. These methods utilize thresholding techniques to screen out weak and invalid instruments. Expanding on these approaches, Lin *et al.* [34] proposed the weak and invalid IV robust treatment effect estimator, which avoids the potential loss of instrument information caused by hard-thresholding selection.

In the study of nonlinear causal inference, much of the existing research focuses on estimating the nonlinear association between exposure and outcome, employing approaches such as machine learning methods, including TSCI [35], Deep IV [36], DeLIVR [37], and Quantile IV [38], as well as control function methods [39, 40]. These approaches excel at modeling complex relationships without strong parametric assumptions, making them powerful tools for general-purpose counterfactual prediction. However, they are not specifically designed to account for weak instruments or horizontal pleiotropy, both of which are central challenges in applied causal inference. Consequently, research on nonlinear causal inference with complex instrument sets, particularly under the simultaneous presence of weak and invalid instruments, remains limited.

To address this gap, we propose model-averaged control function-based instrumental variable regression (MACFIV), a two-stage IV regression framework tailored specifically for robust nonlinear causal inference in complex genetic settings. Our method is based on a model-averaged control function approach to estimate the derivative of the exposure–outcome relationship, referred to as the marginal effect function [41]. The novelty of our framework lies in its targeted design to simultaneously tackle the dual challenges of weak instruments and pleiotropy within a unified, interpretable semi-parametric structure. Specifically, our method incorporates model averaging to enhance estimation stability in the presence of weak genetic instruments and applies SCAD penalization to mitigate biases introduced by pleiotropic instruments. We provide theoretical guarantees for the validity of the method and support our conclusions through simulation studies.

The rest of the paper is organized as follows. First, we introduce the proposed IV regression framework based on model-averaged control function and provide a description of the algorithm and the theoretical results related to this method. Then we evaluate the performance of the proposed approach under various scenarios and compare it with existing methods, and consider an application of the method to the Atherosclerosis Risk in Communities dataset, illustrating the nonlinear relationship between body mass index (BMI) and hypertension-related indicators. Finally, we provide some related discussions. All proofs and supplementary simulation results are provided in the supplementary material.

## Materials and methods

### Nonlinear modeling of causal effect

We consider the following structural equation model:


(1) \begin{align*} & x = \boldsymbol{g}^{T}\boldsymbol{\gamma} + v, \end{align*}

(2) \begin{align*} & y = f(x)+\boldsymbol{g}^{T}\boldsymbol{\alpha} + u, \end{align*}

where $y$ is the scalar outcome, $x$ is the scalar exposure, $f(\cdot )$ is the unknown function of interest, $\boldsymbol{g} = (g_{1},\dots ,g_{p})^{T} $ denotes the vector of genetic IVs, and $(u,v)$ are unmeasured errors, $\boldsymbol{\gamma }$ and $\boldsymbol{\alpha }$ are unknown parameters. Due to the presence of unobserved confounding factors, the error terms $u$ and $v$ may be correlated, leading to the endogeneity of the exposure $x$. In this model, we introduce a nonlinear association between the exposure $x$ and the outcome $y$, which has been widely considered in the previous literature [8, 40, 42]. In our sample analysis, we use $\boldsymbol{X}\in \mathbb{R}^{n}$ and $\boldsymbol{Y}\in \mathbb{R}^{n}$ represent the exposure vector and outcome vector, where $x_{i}\in \mathbb{R}$ and $y_{i}\in \mathbb{R}$ represent the exposure and outcome values, respectively, for the $i$th observation. The genetic instruments $\boldsymbol{G}$ form a matrix in $\mathbb{R}^{n\times p}$, where each row corresponds to the $p$ IVs for a single observation. The residuals from the first-stage regression of $\boldsymbol{X}$ on $\boldsymbol{G}$ are denoted as $\boldsymbol{v}\in \mathbb{R}^{n}$, while the error term in the structural equation model for the outcome $\boldsymbol{Y}$ is denoted as $\boldsymbol{u}\in \mathbb{R}^{n}$. We assume that samples $\mathcal{D}=\left \{\boldsymbol{G}, \boldsymbol{X}, \boldsymbol{Y}\right \}$ is independently and identically distributed with each observation represented by $\left \{\boldsymbol{g}^{T}_{ i}, x_{ i}, y_{ i}\right \}, 1 \leq i \leq n$.

The following definitions summarize the concepts of interest:

Definition 1. The derivative function $f^{\prime }(\cdot )$ of $f(\cdot )$ is called the marginal effect function.

Definition 2. Genetic instrument $g_{j}, j\in \left \{1,\dots ,p\right \}$ is valid if $\alpha _{j} = 0$, and it is invalid if $\alpha _{j} \ne 0$. Let $\mathcal{A}_{I}$ denote the set of invalid instruments or pleiotropic instruments.

Definition 3. Genetic instrument $g_{j}, j\in \left \{1,\dots ,p\right \}$ is a relevant instrument if $\gamma _{j} \ne 0$, and is considered a weak instrument if the F-statistic for its regression on exposure $x$ is $<10$.

In our nonlinear model, our primary focus is on the estimation and statistical inference of the derivative function $f^{\prime }(\cdot )$ and on reducing estimation error in the presence of weak and pleiotropic instruments. The derivative function $f^{\prime }(\cdot )$ represents the instantaneous rate at which the outcome changes with respect to the exposure. It therefore provides direct information about how the strength or direction of the causal relationship varies across exposure levels and reveals features such as turning points, thresholds, and regions of saturation. In contrast, the structural function $f(x)$ itself describes the overall level of the relationship but does not correspond to a causal effect, and in settings with IVs its level is identified only up to an additive constant. From the perspective of potential outcomes, the causal effect of a continuous exposure is defined as the derivative of the mean potential outcome with respect to the treatment level. Let $Y(x)$ be the potential outcome under the intervention $X=x$. Under the structural equation (2), we obtain


\begin{align*} &Y(x) = f(x)+\boldsymbol{g}^T\boldsymbol{\alpha} + u.\end{align*}

For any conditioning variable $\boldsymbol{g}$, differentiating both sides with respect to $x$ yields


\begin{align*} &\frac{\partial}{\partial x}\mathbb{E}\left[Y(x)\mid\boldsymbol{g}\right] = f^{\prime}(x).\end{align*}

Thus, the derivative of the structural function corresponds exactly to the marginal treatment effect for a continuous exposure. This establishes $f^{\prime }(x)$ as the causal estimand in our setting.

In this paper, we use the control function approach [43] for identifying the nonlinear function $f(\cdot )$. Specifically, the control function is implemented by including the residuals from the first-stage regression as an additional covariate in the second-stage model. Compared to traditional two-stage regression, extensive literature has indicated that the control function has advantages in estimating nonlinear models. Specifically, we present the following conditions:


(3) \begin{align*}& \mathbb{E}\left[u\mid v, \boldsymbol{g}\right]=\mathbb{E}\left[u \mid v\right],\end{align*}

which is widely used in the literature. This condition can be viewed as a reformulation of the standard IV requirement that the instruments do not enter the structural outcome equation except through the exposure. In applied settings, it means that any unobserved factors shared by the exposure and the outcome are absorbed by $v$, so that the instruments do not explain the remaining variation in the outcome. Furthermore, we assume a linear relationship between $u$ and $v$, that is, we have the following decomposition:


(4) \begin{align*}& u=\rho v+e, \quad \text{ with} \quad \mathbb{E}\left[e \mid \boldsymbol{g}, v\right]=\mathbb{E}\left[e \mid v\right]=0.\end{align*}

For convenience, we further assume that all data are centered to omit the intercept term. This linear specification of the control function is the conventional assumption in the literature and serves as the basis for our main development. For completeness, we also provide in the supplementary material a complementary extension that allows for nonlinear control functions, which follows essentially the same two-step estimation strategy with only a minor augmentation in the second stage. This extension allows the model to capture more complex dependency structures between the error terms.

### Estimation of the marginal effect function

To estimate the marginal effect function, we first estimate the nonlinear function $f(\cdot )$. We use B-spline basis functions to approximate $f(\cdot )$. In our study, the choice of B-spline basis functions is motivated by their ability to capture complex nonlinear relationships, such as U-shaped or threshold effects. B-splines provide a structured yet flexible approach to modeling these relationships, making them particularly suitable for our causal inference framework. Compared to other nonparametric methods such as kernel smoothing and local polynomial regression, B-splines offer better control over smoothness. Furthermore, B-splines have strong theoretical properties in two-stage control function frameworks, as demonstrated in Fan *et al.* [8], which highlights their excellent convergence properties in such settings.

Let $S$ be the space of polynomial splines of degree $d>1$, from which we select the B-spline basis functions. After centering, we obtain the centered B-spline basis functions $\left \{B_{k}, k=1,\dots ,m\right \}$. Under sufficient smoothness assumptions, we can approximate $f(x)$ using B-spline basis functions by choosing coefficients $\left \{\beta _{1},\dots ,\beta _{m}\right \}$, that is


(5) \begin{align*}& f(x) \approx \sum_{k=1}^{m}\beta_{k}B_{k}(x).\end{align*}

Then the nonlinear model (2) can be written as


(6) \begin{align*}& y \approx \sum_{k=1}^{m}\beta_{k}B_{k}(x)+\boldsymbol{g}^{T}\boldsymbol{\alpha} + u.\end{align*}

Denote $\boldsymbol{B}=\left (B_{1}(x),\dots ,B_{m}(x)\right )^{T}$, $\boldsymbol{\beta }=\left (\beta _{1},\dots ,\beta _{m}\right )^{T}$, then (6) can be rewritten as


(7) \begin{align*}& y \approx \boldsymbol{B}^{T}\boldsymbol{\beta}+\boldsymbol{g}^{T}\boldsymbol{\alpha} + u.\end{align*}

Next, we consider a two-stage estimation framework. Taking into account the potential presence of weak instruments, we use a model averaging framework in the first stage to reduce errors caused by weak instruments. We rewrite equation (1) in matrix form for the sample:


\begin{align*} & \boldsymbol{X} = \boldsymbol{G} \boldsymbol{\gamma} + \boldsymbol{v}. \end{align*}

We assume that the $p$ instruments can be divided into ordered groups, i.e. $\boldsymbol{g}_{ i}=\left (\boldsymbol{g}^{T}_{1i},\dots ,\boldsymbol{g}^{T}_{Qi}\right )^{T}$, where $\boldsymbol{g}_{qi}$ is $p_{q}\times 1$ and the total number of predictors is $p=p_{1}+\cdots +p_{Q}$. Instead of using all the $p$ predictors of $\boldsymbol{G}$ to get $\hat{\boldsymbol{\gamma }}$, we consider $Q$ nested models, where the $q$th model can be written as:


(8) \begin{align*}& x_{i} = \tilde{\boldsymbol{g}}_{qi}^{T}\boldsymbol{\gamma}_{q} + v_{qi},\end{align*}

where $\tilde{\boldsymbol{g}}_{qi} = \left (\boldsymbol{g}^{T}_{1i},\dots ,\boldsymbol{g}^{T}_{qi}\right )^{T} = \boldsymbol{\Pi }_{q}\boldsymbol{g}_{i}$ and $\boldsymbol{\Pi }_{q} = \left (\boldsymbol{I}_{K_{q}}, \boldsymbol{0}_{K_{q}\times \left (p-K_{q}\right )}\right )$ is a $K_{q}\times p$ projection matrix with $K_{q} = p_{1}+\cdots +p_{q}$. The number of groups $Q$ is determined based on the number of instruments and their association strength with the exposure. To group the instruments, we first calculate the effect sizes for each instrument in relation to the exposure and rank the instruments accordingly. The grouping scheme is flexible and can be adapted based on the number of instruments, it is common to either assign each instrument to its own group (resulting in $Q = p$) or group a fixed number of instruments together [44]. To prevent overfitting when the number of instruments is large, we can adopt the widely used rule $Q=[3n^{\frac{1}{3}}]$ to ensure the number of groups remains manageable while preserving the robustness of the model averaging method [18].

For each submodel, we can obtain the estimate $\tilde{\boldsymbol{\gamma }}_{q}$ using the $q$th model through least squares. Then the estimator of $\boldsymbol{\gamma }$ using the $q$th model is $\hat{\boldsymbol{\gamma }}_{q}=\boldsymbol{\Pi }_{q}^{T}\tilde{\boldsymbol{\gamma }}_{q}$. Let $\boldsymbol{w}=\left (w_{1}, \dots , w_{ Q}\right )^{T}$ be a weight vector in the unit simplex $\mathcal{H}_{Q}=\left \{\boldsymbol{w} \in [0,1]^{Q}: \sum _{q=1}^{Q} w_{q}=1\right \}$. The averaging estimator of $\boldsymbol{\gamma }$ is


\begin{align*} & \hat{\boldsymbol{\gamma}}\left(\boldsymbol{w}\right)=\sum_{q=1}^Q w_{q} \hat{\boldsymbol{\gamma}}_q. \end{align*}

We can use the Mallows criterion, commonly applied in model averaging methods, to determine the optimal weights. Specifically, the optimal weights can be obtained by minimizing the following objective function:


(9) \begin{align*}& \mathcal{C}_{n}\left(\boldsymbol{w}\right)=\sum_{i=1}^{n}\left\{ x_{i} - \boldsymbol{g}_{ i}^{T}\hat{\boldsymbol{\gamma}}\left(\boldsymbol{w}\right) \right\}^{2}+2 \hat{\sigma}^{2} \sum_{q=1}^{Q} w_{q} K_{q},\end{align*}

where $\hat{\sigma }^{2}=\sum _{i=1}^{n}\left (x_{i}-\boldsymbol{g}_{i}^{T} \hat{\boldsymbol{\gamma }}_{Q}\right )^{2} /(n-p)$. Denote $\hat{\boldsymbol{w}}=\underset{\boldsymbol{w} \in \mathcal{H}_{Q}}{\arg \min } \ \mathcal{C}_{n}\left (\boldsymbol{w}\right )$, then the model averaging estimate of $\boldsymbol{\gamma }$ is given by:


(10) \begin{align*}& \hat{\boldsymbol{\gamma}}\left(\hat{\boldsymbol{w}}\right) =\sum_{q=1}^{Q} \hat{w}_{q} \hat{\boldsymbol{\gamma}}_{q}.\end{align*}

Thus, we can obtain the residuals from the model averaging estimate:


(11) \begin{align*}& \hat{\boldsymbol{v}}=\boldsymbol{X}-\boldsymbol{G} \hat{\boldsymbol{\gamma}}\left(\hat{\boldsymbol{w}}\right),\end{align*}

which we use as an estimate of $\boldsymbol{v}$. The use of several models in this stage is important because a single model may not represent the instrument–exposure relationship well. If a model contains many weak instruments, the fitted exposure carries substantial noise and the control function becomes inaccurate. If a model includes only a small number of strong instruments, it may leave out instruments that still contain useful information, which also results in an incorrect model. Since it is difficult to know in advance which set of instruments is most appropriate, using only one model can lead to unstable results. By averaging the fitted values from all Q models, the procedure assigns larger weights to models with better prediction accuracy and smaller weights to models influenced by weak instruments or by the omission of useful instruments. In this way, the averaged control function captures the stable part of the instrument–exposure relationship and provides a more reliable input for the second stage.

We then describe the second-stage procedure to select invalid instruments and, based on that, estimate the marginal effect function. Combining the assumption of the control function approach from equation (4) with the decomposition form of the B-spline basis functions from equation (7), we obtain:


(12) \begin{align*}& y \approx \boldsymbol{B}^{T}\boldsymbol{\beta}+\boldsymbol{g}^{T}\boldsymbol{\alpha} + \rho v + e.\end{align*}

Denote $\boldsymbol{B}_{i}=\left (B_{1}(x_{i}),\cdots ,B_{m}(x_{i})\right )^{T}$, $\mathcal{B}=\left (\boldsymbol{B}_{1},\cdots , \boldsymbol{B}_{n}\right )^{T}$, then the sample form of (12) is given by


(13) \begin{align*}& \boldsymbol{Y} \approx \mathcal{B}\boldsymbol{\beta}+\boldsymbol{G}\boldsymbol{\alpha} + \rho \boldsymbol{v} + \boldsymbol{e}.\end{align*}

Substituting $\hat{\boldsymbol{v}}$ from (11) into (13), we have


(14) \begin{align*}& \boldsymbol{Y} \approx \mathcal{B}\boldsymbol{\beta}+\boldsymbol{G}\boldsymbol{\alpha} + \rho \hat{\boldsymbol{v}} + \boldsymbol{e}^{\prime}.\end{align*}

We separate the bias caused by invalid instruments from the estimation of the coefficient vector $\boldsymbol{\beta }$ through sparse regression:


(15) \begin{align*}& \min_{\boldsymbol{\alpha},\boldsymbol{\beta},\rho}\left\{\left\|\boldsymbol{Y} - \mathcal{B}\boldsymbol{\beta}-\boldsymbol{G}\boldsymbol{\alpha} - \rho \hat{\boldsymbol{v}}\right\|_{2}^{2}\right\} \quad \text{ s.t. }\|\boldsymbol{\alpha}\|_{0} \leq K,\end{align*}

where $\|\boldsymbol{\alpha }\|_{0}=\sum _{j=1}^{p}\text{I}(\alpha _{j}\ne 0)$ and $K\ge 0$ is an integer tuning parameter that controls the number of invalid instruments.

To facilitate computation, we replace the $L_{0}$-norm penalty with a more manageable surrogate while preserving its sparsity-inducing properties. Many regularization methods, such as LASSO [31, 32], SCAD [7], and MCP [34], have been proposed as substitutes for $L_{0}$ and successfully applied in similar two-stage frameworks. These methods achieve sparsity by shrinking small coefficients to zero while remaining computationally feasible. Lin *et al.* [34] further provided theoretical support for their effectiveness under appropriate conditions. In our model, we adopt the SCAD penalty, which not only selects variables but also satisfies the oracle property [45]. We formulate the objective function with the SCAD penalty as follows:


(16) \begin{align*}& \left(\hat{\boldsymbol{\alpha}},\hat{\boldsymbol{\beta}},\hat{\rho}\right) = \underset{\boldsymbol{\alpha},\boldsymbol{\beta},\rho}{\arg\min}\left\{\left\|\boldsymbol{Y} - \mathcal{B}\boldsymbol{\beta}-\boldsymbol{G}\boldsymbol{\alpha} - \rho \hat{\boldsymbol{v}}\right\|_{2}^{2} + \sum_{j=1}^{p}p_{\lambda}^{\text{SCAD}}(\alpha_{j})\right\},\end{align*}

where


\begin{align*} &p_{\lambda}^{\text{SCAD}}(\alpha_j)=\begin{cases}\lambda\left|\alpha_j\right|, & \text{ if}\ \left|\alpha_j\right| \leq \lambda, \\ -\frac{\alpha_j^2-2 a \lambda\left|\alpha_j\right|+{\lambda}^2}{2(a-1)}, & \text{ if}\ \lambda<\left|\alpha_j\right| \leq a \lambda, \\ \frac{(a+1) {\lambda}^2}{2}, & \text{ if}\ \left|\alpha_j\right|>a \lambda,\end{cases}\end{align*}

for some $a> 2$ and $\lambda>0$. Based on the above procedure, we obtain the estimate $\hat{\boldsymbol{\alpha }}$ for $\boldsymbol{\alpha }$ and an estimate of the set of invalid instruments $\hat{\mathcal{A}}_{I} = \left \{g_{j}: \hat{\alpha }_{j} \ne 0\right \}$. Besides, we obtain an estimate of the coefficient vector $\hat{\boldsymbol{\beta }}$ and, therefore, obtain an estimate of the nonlinear function $f(x)$:


(17) \begin{align*}& \hat{f}(x) = \sum_{k=1}^{m}\hat{\beta}_{k}B_{k}(x).\end{align*}

Then the plug-in estimator for the marginal effect function $f^{\prime }(x)$ is


(18) \begin{align*}& \hat{f}^{\prime}(x) = \sum_{k=1}^{m}\hat{\beta}_{k}B^{\prime}_{k}(x).\end{align*}

In summary, the proposed procedure, within the framework of the control function approach, uses model averaging and SCAD to reduce bias from weak and pleiotropic genetic instruments and provides an estimate of the marginal effect function. We refer to this method as MACFIV, as summarized in Algorithm 1.



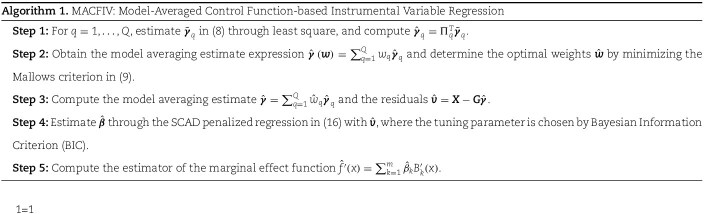



The framework can also be extended to binary or categorical outcomes by adopting generalized link functions, such as a logistic link for binary responses. Under this formulation, the nonlinear exposure–outcome relationship can be modeled in the same way, and the control function structure remains applicable for endogeneity adjustment. A brief discussion of this potential extension and related implementation considerations is provided in the supplementary material.

### Asymptotic properties

According to the above algorithm, our estimation of the marginal effect function $\hat{f}^{\prime }(x)$ should satisfy the following asymptotic properties.

Theorem 1.1. Suppose that Assumptions provided in the supplementary material hold, and assume the number of spline bases satisfy $m\asymp n^{\nu }$ with $\nu \geq \frac{1}{2(\theta -1)}$, then the estimate $\hat{f}^{\prime }(x)$ satisfies

(19) \begin{align*} & \underset{f}{\sup}\int_{\left[a,b\right]}\left[\hat{f}^{\prime}(x)-f^{\prime}(x)\right]^{2} = O_{p}(m^{-2\theta+2}), \end{align*}

 

(20) \begin{align*} & \sqrt{n}\left(\hat{f}^{\prime}(x)-f^{\prime}(x)\right) = \mathcal{N}\left(0, \boldsymbol{B}^{\prime}\left(x\right)^{T}\boldsymbol{U}\boldsymbol{B}^{\prime}\left(x\right)\right) + o_{p}(1), \end{align*}

The proof of Theorem 1.1, as well as the definition of $\boldsymbol{U}$, are given in Supplementary Section S1. The above asymptotic results indicate that we can obtain an accurate estimate of the marginal effect function, with the estimate showing good distributional properties that make subsequent statistical inference easier. In addition, we note that the residual from the first stage differs from the true error term by an additional component arising from the estimation of the first-stage parameters. While this term vanishes asymptotically under the stated regularity conditions and does not affect the limiting distribution in Theorem 1.1, it may, in finite samples, introduce extra variability that can slightly affect bias and variance estimation, as well as the construction of the final confidence intervals.

## Simulations

In this section, we conduct various simulation studies to evaluate the performance of our proposed method compared with other methods. We generate data based on a nonlinear structural equation model,


\begin{align*} &x_i = \boldsymbol{g}_i^T\boldsymbol{\gamma} + v_i,\end{align*}

\begin{align*} &y_i = f(x_i)+\boldsymbol{g_i}^T\boldsymbol{\alpha} + u_i.\end{align*}

The true causal relationship between the exposure $x$ and the outcome $y$ is defined by the nonlinear function $f(x)$, and the true causal effect is represented by the derivative $f^{\prime }(x)$. Specifically, when $f(x)$ is a linear function, that is, $f(x)=\beta x$, the ground-truth effect is $\beta $, which reduces to the problem of linear causal inference.

To reflect real-world scenarios, we set categorical IVs. Specifically, $\boldsymbol{g}_{i}^{T}=\left (g_{i1},\dots g_{ip}\right )^{T}$ are generated as $\boldsymbol{g}_{i} = \boldsymbol{\tau }_{i} + \boldsymbol{\xi }_{i}$ and then standardized, where $\boldsymbol{\tau }_{i}$ and $\boldsymbol{\xi }_{i}$ are independent Bernoulli random variables with a success probability of 0.3. For continuous IVs, we have considered them in Supplementary Section S2. To account for the presence of pleiotropy instruments, we set $\boldsymbol{\alpha } = \left (\boldsymbol{1}_{s}, \boldsymbol{0}_{p-s}\right )$, which means the first $s$ instruments are invalid, and the remaining instruments are valid. We also consider other magnitudes of pleiotropic effects in Supplementary Section S2. Additionally, we set $\boldsymbol{\gamma } = \left (\frac{\mu }{\sqrt{n}},\dots ,\frac{\mu }{\sqrt{n}}\right )$ to introduce weak instruments, following the “many weak” design by Fan and Wu [13], where $\mu =\sqrt{2}$. We precompute the F-statistics for the association between the instruments and the exposure under the “many weak” setting, considering different sample sizes and numbers of instruments. The average F-statistics ranged from 3.94 to 3.98, indicating that the instruments are weak (as an F-statistic $<10$ is typically considered indicative of weak instruments). The detailed distribution of F-statistics for each scenario is provided in Supplementary Table S1. For the error terms, we adopt the following generation scheme:


\begin{align*} &c_i \sim \mathcal{N}(0,1),\quad \epsilon_i \sim \mathcal{N}(0,1),\quad v_i = c_i + \epsilon_i,\end{align*}

and


\begin{align*} &e_i \sim \mathcal{N}(0,1),\quad u_i = v_i + e_i,\end{align*}

where $c_{i}$ acts as a confounder. For the nonlinear function $f(\cdot )$, we consider the following common functional forms and their combinations:

1. Null: $f(x)=0$
2. Linear: $f(x)=x$
3. Quadratic: $f(x)=0.01\cdot x^{2}$
4. Trigonometric: $f(x)=\sin (0.1\cdot x)$
5. Exponential: $f(x)=\exp (0.1\cdot x)$
6. Logarithmic: $f(x)=\log (x) \quad (x>0)$
7. Mixed: $f(x)=\cos (0.1\cdot x) + \exp (0.1\cdot x) + 0.01\cdot x^{2}$

We use cubic B-spline basis functions to approximate the nonlinear function $f(x)$ and set the number of basis functions to $m=5$ concerning Lu *et al.* [46]. As noted by Fan *et al.* [13], increasing the number of basis functions does not benefit the estimation variance. Additionally, we provide the parameter settings for model averaging and SCAD penalization. Before conducting the simulations, we first calculate the absolute values of the sample Pearson correlation coefficients between each instrument and the exposure $x$, then rank the instruments in descending order based on these absolute values. We also consider other orderings of instruments in Supplementary Section S2. For the ordered set of instruments, we set $p_{1}=\dots =p_{Q}=1$, which means we construct $Q=p$ nested models. In the $q$th model, the instrument set consists of the top $q$ instruments from the ordered list, and the parameter $K_{q}=p_{1}+\dots +p_{q}=q$. For the SCAD penalization step, we set $a=3.7$ following the theory of Fan and Li [45], and select the parameter $\lambda $ using the BIC. We also consider using joint cross-validation to simultaneously select the number of basis functions $m$ and the penalty parameter $\lambda $, and this part is presented in Supplementary Section S2.

To contextualize the performance of MACFIV, we compare it with several benchmark methods commonly used in IV analysis and nonlinear causal inference. These include:

1. TSP (two-stage prediction): a traditional nonlinear IV method, included as a baseline for comparison with nonlinear approaches.
2. TSP-SCAD (two-stage prediction with SCAD penalty): an extension of TSP that incorporates the SCAD penalty to handle invalid instruments, included to evaluate the impact of penalization in addressing pleiotropy and to contrast with MACFIV’s approach to handling pleiotropic instruments.
3. DeepIV: a deep learning-based nonlinear IV method [36], included to compare MACFIV with emerging deep learning approaches for nonlinear causal inference.
4. PolyMR (polynomial Mendelian randomization): a nonlinear Mendelian randomization method that uses polynomial approximations [42], included to compare the performance of different basis function approximations and to assess how MACFIV performs in settings with weak and pleiotropic instruments.
5. CF (control function): a control function-based method for nonlinear causal inference [40], included to compare MACFIV’s performance with traditional control function approaches, particularly in addressing weak instruments and pleiotropy.

For the competing methods, we follow the recommended or commonly used tuning settings in the literature. The SCAD-based procedures use the standard SCAD penalty with the tuning parameter chosen by BIC. All methods are applied using the same sample points and the same first-stage fitted values when applicable, so that the comparison focuses on differences in modeling strategies rather than differences in tuning choices.

To compare the performance of various methods under different parameter settings, we vary the sample size $n$, the number of instruments $p$, the number of invalid instruments $s$, and the proportion of weak instruments. We repeat each simulation $1000$ times and calculate the mean bias, root mean squared error (RMSE), and mean absolute error (MAE) between $f^{\prime }$ and $\hat{f}^{\prime }$ in the samples as evaluation metrics. More specifically, in each simulation replicate, we evaluate the true marginal effect $f^{\prime }$ and the estimated marginal effect $\hat{f}^{\prime }$ at the observed exposure values $\left \{x_{i}\right \}_{i=1}^{n}$, and compute


\begin{align*} &\text{Mean Bias} = \frac{1}{n}\sum_{i=1}^{n}\left[f^{\prime}(x_i)-\hat{f}^{\prime}(x_i)\right],\end{align*}

\begin{align*} &\text{RMSE} = \sqrt{\frac{1}{n}\sum_{i=1}^{n}\left[f^{\prime}(x_i)-\hat{f}^{\prime}(x_i)\right]^2},\end{align*}

\begin{align*} &\text{MAE} = \frac{1}{n}\sum_{i=1}^{n}\left|f^{\prime}(x_i)-\hat{f}^{\prime}(x_i)\right|.\end{align*}

These quantities measure how close the estimated marginal effect function is to the true one at the sample points. Furthermore, for ease of analyzing the results, we designed the following simulation scenarios.

**Scenario 1: change the number of invalid instruments $s$**

**Figure 1:**
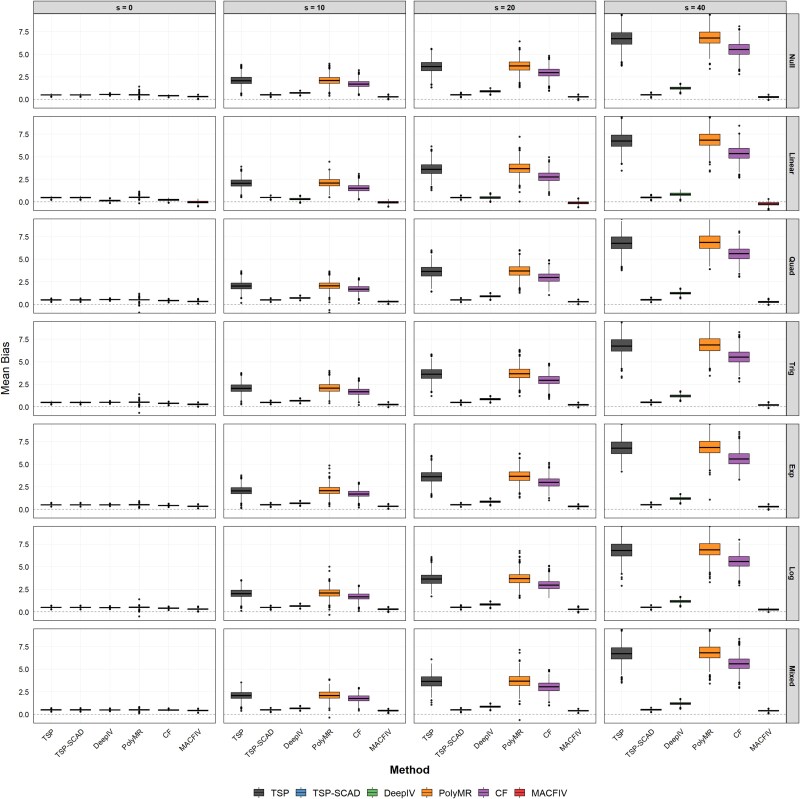
Boxplots of the estimation bias for the marginal effect function $f^{\prime }$ in Scenario 1: fix $n=2000$, $p=100$, change the number of pleiotropy instruments $s=0,10,20,40$.

First, we consider the impact of the number of invalid instruments. In this scenario, we fix $n=2000$, $p=100$, while varying the number of invalid instruments $s=0,10,20,40$ to investigate the impact of the number of invalid instruments on estimation. Figure 1 presents the mean bias for the estimated marginal effect function across the four settings using boxplots, and Table 1 reports the corresponding RMSE results. The MAE results are provided in Supplementary Section S2.

**Table 1:** Mean and standard deviation of RMSE results for estimating the marginal effect function $f^{\prime }$ in Scenario 1: fix $n=2000$, $p=100$, change the number of pleiotropy instruments $s=0,10,20,40$

		TSP		TSP-SCAD		DeepIV		PolyMR		CF		MACFIV	
	$f$	Mean	SD	Mean	SD	Mean	SD	Mean	SD	Mean	SD	Mean	SD
$s=0$	Null	0.616	0.100	0.615	0.100	0.556	0.049	1.016	1.347	0.429	0.062	0.314	0.084
	Linear	0.763	0.121	0.763	0.121	0.351	0.074	0.956	1.603	0.455	0.064	0.341	0.066
	Quad	0.613	0.102	0.613	0.102	0.557	0.049	1.053	1.916	0.431	0.061	0.323	0.081
	Trig	0.620	0.099	0.620	0.098	0.522	0.053	1.069	1.691	0.420	0.062	0.295	0.084
	Exp	0.605	0.100	0.605	0.100	0.521	0.053	1.040	1.777	0.440	0.062	0.344	0.080
	Log	0.609	0.098	0.609	0.098	0.495	0.057	1.103	3.026	0.430	0.062	0.317	0.082
	Mixed	0.604	0.097	0.603	0.097	0.521	0.053	1.038	1.683	0.477	0.057	0.411	0.072
$s=10$	Null	3.833	1.126	0.624	0.109	0.731	0.090	3.490	5.747	1.803	0.432	0.300	0.085
	Linear	3.854	1.141	0.771	0.127	0.432	0.109	3.968	5.568	1.728	0.427	0.336	0.069
	Quad	3.756	1.183	0.623	0.110	0.731	0.091	3.501	5.553	1.774	0.434	0.315	0.081
	Trig	3.806	1.152	0.630	0.105	0.695	0.093	3.820	5.956	1.766	0.433	0.282	0.087
	Exp	3.813	1.177	0.618	0.109	0.695	0.093	3.998	9.590	1.795	0.416	0.337	0.083
	Log	3.775	1.127	0.628	0.104	0.672	0.097	4.034	8.835	1.765	0.439	0.309	0.082
	Mixed	3.781	1.091	0.609	0.103	0.695	0.093	3.513	4.768	1.818	0.420	0.412	0.072
$s=20$	Null	5.759	1.488	0.637	0.117	0.907	0.116	5.501	7.396	3.147	0.583	0.284	0.089
	Linear	5.857	1.517	0.776	0.130	0.573	0.135	5.953	8.709	3.045	0.594	0.340	0.073
	Quad	5.790	1.477	0.638	0.121	0.907	0.116	5.862	8.448	3.112	0.571	0.300	0.087
	Trig	5.827	1.468	0.647	0.114	0.870	0.119	5.570	8.118	3.121	0.593	0.266	0.088
	Exp	5.795	1.505	0.631	0.125	0.869	0.119	5.592	5.737	3.146	0.575	0.329	0.082
	Log	5.780	1.486	0.637	0.119	0.840	0.120	6.462	24.086	3.140	0.598	0.289	0.090
	Mixed	5.737	1.474	0.626	0.117	0.869	0.118	5.940	9.577	3.186	0.560	0.410	0.077
$s=40$	Null	9.272	1.732	0.677	0.139	1.260	0.164	8.857	7.308	5.884	0.811	0.260	0.099
	Linear	9.250	1.786	0.808	0.153	0.895	0.187	8.856	7.470	5.722	0.808	0.370	0.110
	Quad	9.232	1.769	0.686	0.157	1.260	0.165	9.209	10.436	5.833	0.818	0.284	0.097
	Trig	9.292	1.799	0.679	0.139	1.222	0.167	10.065	44.805	5.798	0.802	0.233	0.095
	Exp	9.217	1.784	0.671	0.142	1.221	0.166	9.502	12.164	5.864	0.826	0.309	0.096
	Log	9.195	1.809	0.671	0.134	1.192	0.168	10.109	31.244	5.843	0.778	0.269	0.098
	Mixed	9.153	1.753	0.656	0.136	1.220	0.167	8.790	7.262	5.872	0.803	0.401	0.084

**Scenario 2: change the number of sample size $n$**

**Figure 2:**
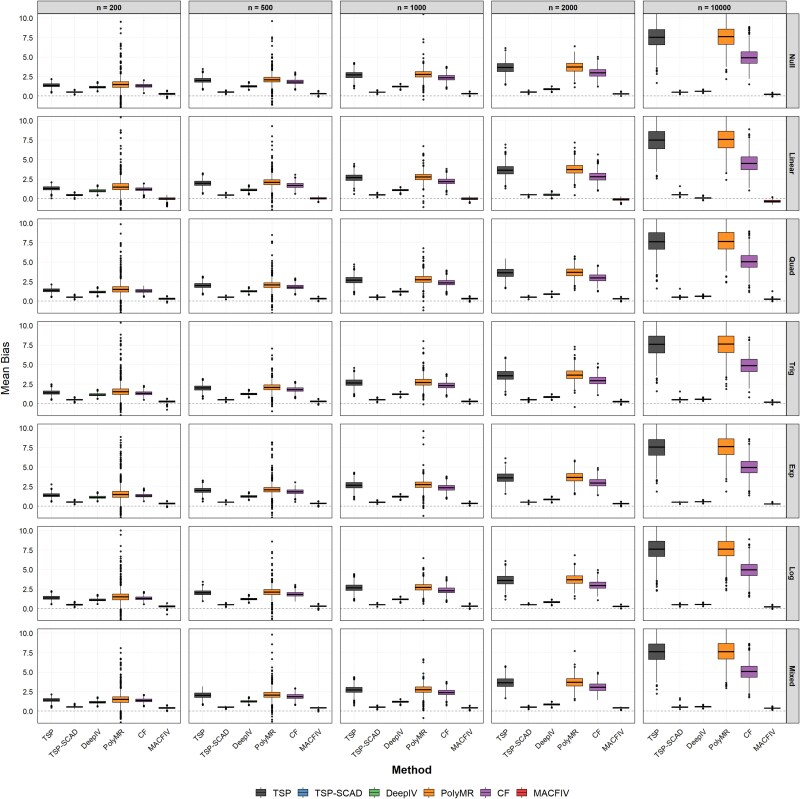
Boxplots of the estimation bias for the marginal effect function $f^{\prime }$ in Scenario 2: fix $p=100$, $s=20$, change the sample size $n=200,500,1000,2000, 10\,000$.

Next, to better demonstrate the asymptotic behavior, we consider the impact of sample size on the estimation performance, including the behavior in small sample sizes. In this scenario, we fix the number of instruments $p=100$, and the number of invalid instruments $s=20$, while varying the sample size $n=200,500,1000,2000,10\,000$. Figure 2 summarizes the mean bias results for the estimation of the marginal effect function in this scenario using boxplots, and the RMSE results are presented in Table 2. The MAE results are summarized in Supplementary Section S2.

**Table 2:** Mean and standard deviation of RMSE results for estimating the marginal effect function $f^{\prime }$ in Scenario 2: fix $p=100$, $s=20$, change the sample size $n=200,500,1000,2000, 10\,000$

		TSP		TSP-SCAD		DeepIV		PolyMR		CF		MACFIV	
	$f$	Mean	SD	Mean	SD	Mean	SD	Mean	SD	Mean	SD	Mean	SD
$n=200$	Null	2.171	0.497	0.723	0.145	1.216	0.190	14.511	38.480	1.762	0.334	0.403	0.126
	Linear	2.502	0.573	1.176	0.280	1.094	0.210	18.544	92.615	2.067	0.413	0.729	0.196
	Quad	2.145	0.499	0.720	0.154	1.217	0.190	15.825	46.916	1.750	0.336	0.406	0.130
	Trig	2.182	0.488	0.760	0.160	1.203	0.192	20.701	103.119	1.774	0.320	0.414	0.136
	Exp	2.146	0.491	0.691	0.143	1.202	0.192	15.484	40.705	1.732	0.317	0.405	0.125
	Log	2.186	0.498	0.733	0.158	1.191	0.194	14.103	49.617	1.767	0.322	0.403	0.134
	Mixed	2.105	0.497	0.670	0.131	1.203	0.193	14.916	50.278	1.679	0.311	0.453	0.109
$n=500$	Null	3.131	0.756	0.668	0.123	1.282	0.149	10.887	58.936	2.082	0.365	0.353	0.100
	Linear	3.284	0.738	0.934	0.178	1.171	0.166	9.834	27.542	2.205	0.370	0.521	0.112
	Quad	3.115	0.769	0.663	0.122	1.283	0.149	13.171	90.007	2.069	0.351	0.364	0.099
	Trig	3.200	0.784	0.686	0.131	1.271	0.151	11.298	54.425	2.088	0.365	0.344	0.102
	Exp	3.136	0.808	0.646	0.118	1.270	0.151	9.145	26.566	2.052	0.372	0.368	0.099
	Log	3.167	0.789	0.672	0.128	1.259	0.152	9.355	28.206	2.082	0.377	0.359	0.103
	Mixed	3.061	0.747	0.633	0.122	1.271	0.151	9.675	40.065	2.064	0.366	0.424	0.085
$n=1000$	Null	4.210	1.048	0.647	0.122	1.246	0.122	6.381	13.518	2.537	0.456	0.324	0.095
	Linear	4.263	1.016	0.839	0.148	1.136	0.136	6.825	14.069	2.521	0.458	0.413	0.083
	Quad	4.214	1.037	0.641	0.120	1.246	0.121	6.654	14.754	2.528	0.449	0.336	0.095
	Trig	4.223	1.037	0.662	0.126	1.234	0.122	6.975	18.633	2.516	0.455	0.310	0.096
	Exp	4.223	1.067	0.638	0.118	1.234	0.123	6.515	14.163	2.514	0.455	0.350	0.092
	Log	4.232	0.998	0.646	0.117	1.224	0.124	7.056	21.352	2.536	0.449	0.324	0.093
	Mixed	4.222	1.046	0.630	0.120	1.233	0.123	6.641	14.510	2.516	0.442	0.419	0.085
$n=2000$	Null	5.803	1.471	0.633	0.114	0.907	0.116	5.618	6.704	3.129	0.601	0.292	0.091
	Linear	5.869	1.470	0.773	0.130	0.573	0.135	5.435	6.552	3.009	0.594	0.341	0.080
	Quad	5.812	1.476	0.632	0.118	0.908	0.116	5.682	7.131	3.148	0.588	0.303	0.088
	Trig	5.796	1.443	0.648	0.117	0.870	0.119	6.054	9.974	3.146	0.607	0.269	0.087
	Exp	5.808	1.467	0.639	0.120	0.869	0.119	5.402	6.914	3.148	0.591	0.333	0.084
	Log	5.871	1.535	0.639	0.121	0.840	0.120	5.423	7.236	3.129	0.587	0.300	0.092
	Mixed	5.822	1.492	0.623	0.113	0.869	0.118	5.790	12.393	3.176	0.608	0.407	0.078
$n=10\,000$	Null	12.464	3.224	0.601	0.098	0.623	0.068	8.106	2.445	5.122	1.157	0.214	0.081
	Linear	12.413	3.212	0.696	0.119	0.252	0.071	8.029	2.359	4.809	1.224	0.393	0.147
	Quad	12.153	3.059	0.601	0.112	0.624	0.068	7.993	2.710	5.083	1.133	0.246	0.084
	Trig	12.441	3.378	0.613	0.254	0.573	0.070	8.145	3.037	5.075	1.187	0.171	0.079
	Exp	12.323	3.179	0.610	0.105	0.572	0.070	7.951	2.880	5.042	1.089	0.271	0.070
	Log	12.395	3.290	0.610	0.116	0.542	0.072	8.016	2.353	5.062	1.162	0.228	0.080
	Mixed	12.413	3.225	0.604	0.119	0.572	0.070	8.175	3.513	5.215	1.150	0.376	0.058

**Scenario 3: change the number of instruments $p$**

**Figure 3:**
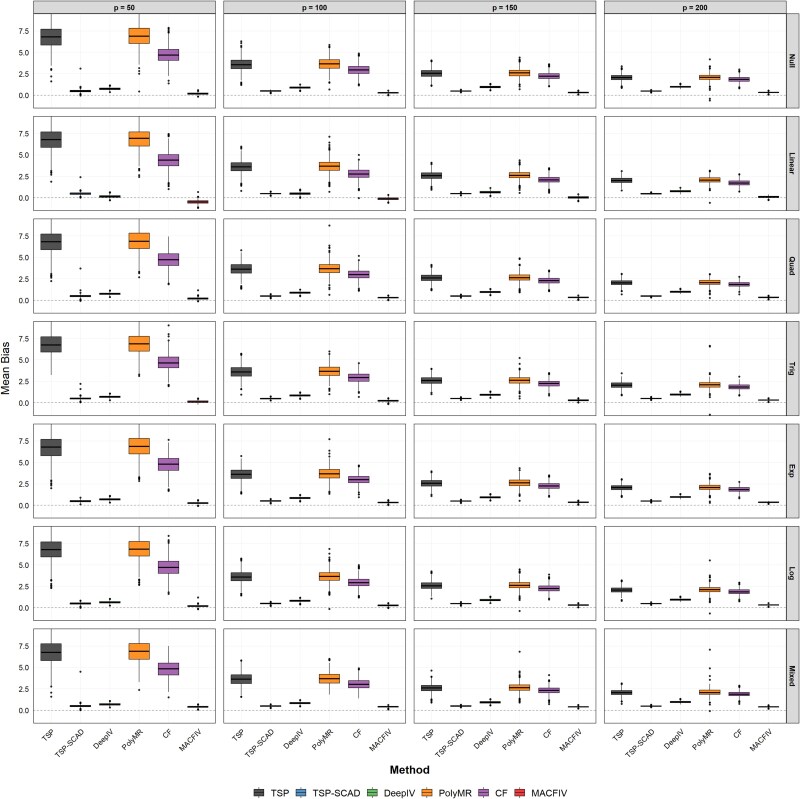
Boxplots of the estimation bias for the marginal effect function $f^{\prime }$ in Scenario 3: fix $n=2000, s=20$, change the number of instruments $p=50,100,150,200$.

We further consider the stability of the estimation in the case of a different number of instruments. In this scenario, we fix the sample size $n=2000$ and the number of invalid instruments $s=20$, while varying the number of instruments $p=50,100,150,200$. Figure 3 summarizes the mean bias results for the estimation of the marginal effect function in this scenario using boxplots, and the RMSE results are presented in Table 3. The MAE results are summarized in Supplementary Section S2.

**Table 3:** Mean and standard deviation of RMSE results for estimating the marginal effect function $f^{\prime }$ in Scenario 3: fix $n=2000, s=20$, change the number of instruments $p=50,100,150,200$

		TSP		TSP-SCAD		DeepIV		PolyMR		CF		MACFIV	
	$f$	Mean	SD	Mean	SD	Mean	SD	Mean	SD	Mean	SD	Mean	SD
$p=50$	Null	11.143	2.858	0.822	0.445	0.781	0.127	9.928	10.771	4.973	0.983	0.199	0.100
	Linear	11.146	2.822	0.919	0.227	0.353	0.123	10.040	16.144	4.726	1.040	0.532	0.184
	Quad	11.122	2.872	0.799	0.309	0.780	0.127	9.575	10.654	4.969	0.989	0.221	0.104
	Trig	11.017	2.869	0.812	0.297	0.726	0.129	10.343	21.616	4.931	0.951	0.172	0.094
	Exp	11.216	3.064	0.809	0.426	0.725	0.130	8.866	6.397	4.991	1.017	0.269	0.105
	Log	10.989	3.012	0.792	0.228	0.683	0.132	9.367	10.576	4.888	0.985	0.207	0.100
	Mixed	11.174	2.876	0.787	0.236	0.726	0.129	9.721	23.485	5.016	0.970	0.400	0.095
$p=100$	Null	5.829	1.500	0.639	0.116	0.907	0.116	5.870	8.533	3.123	0.598	0.290	0.089
	Linear	5.900	1.522	0.778	0.131	0.573	0.135	5.541	6.296	3.067	0.607	0.338	0.079
	Quad	5.792	1.439	0.630	0.117	0.906	0.117	5.882	9.067	3.148	0.617	0.299	0.089
	Trig	5.822	1.509	0.649	0.114	0.870	0.119	5.475	5.871	3.155	0.595	0.271	0.091
	Exp	5.732	1.474	0.634	0.122	0.869	0.119	5.953	7.363	3.148	0.599	0.325	0.086
	Log	5.699	1.419	0.640	0.117	0.840	0.120	5.569	8.061	3.118	0.589	0.296	0.089
	Mixed	5.716	1.408	0.623	0.118	0.869	0.118	5.812	6.683	3.172	0.573	0.409	0.079
$p=150$	Null	3.948	0.984	0.594	0.080	0.978	0.116	4.092	3.913	2.382	0.422	0.326	0.073
	Linear	4.017	0.943	0.742	0.109	0.732	0.138	5.324	20.139	2.359	0.430	0.354	0.061
	Quad	3.956	0.978	0.590	0.079	0.978	0.117	4.490	6.828	2.389	0.421	0.339	0.076
	Trig	3.927	0.979	0.598	0.080	0.951	0.118	4.479	5.424	2.353	0.437	0.311	0.079
	Exp	4.029	1.007	0.582	0.078	0.950	0.118	4.168	5.587	2.360	0.439	0.350	0.074
	Log	3.971	0.983	0.592	0.084	0.931	0.120	4.354	5.168	2.378	0.435	0.331	0.075
	Mixed	3.991	0.955	0.581	0.083	0.951	0.118	4.207	5.333	2.381	0.428	0.406	0.068
$p=200$	Null	3.068	0.730	0.571	0.065	1.001	0.104	3.784	5.355	1.963	0.315	0.347	0.067
	Linear	3.150	0.733	0.723	0.098	0.809	0.124	3.609	5.513	1.972	0.347	0.382	0.063
	Quad	3.060	0.744	0.565	0.064	1.002	0.104	3.680	6.216	1.955	0.328	0.354	0.067
	Trig	3.034	0.717	0.581	0.067	0.981	0.106	3.680	7.184	1.944	0.335	0.338	0.068
	Exp	3.038	0.705	0.565	0.062	0.980	0.106	3.657	4.249	1.968	0.337	0.365	0.063
	Log	3.088	0.728	0.575	0.064	0.966	0.108	4.469	17.354	1.955	0.333	0.349	0.068
	Mixed	3.025	0.710	0.562	0.063	0.981	0.106	3.622	5.815	1.966	0.344	0.410	0.059

**Scenario 4: change the proportion of weak instruments**

**Figure 4:**
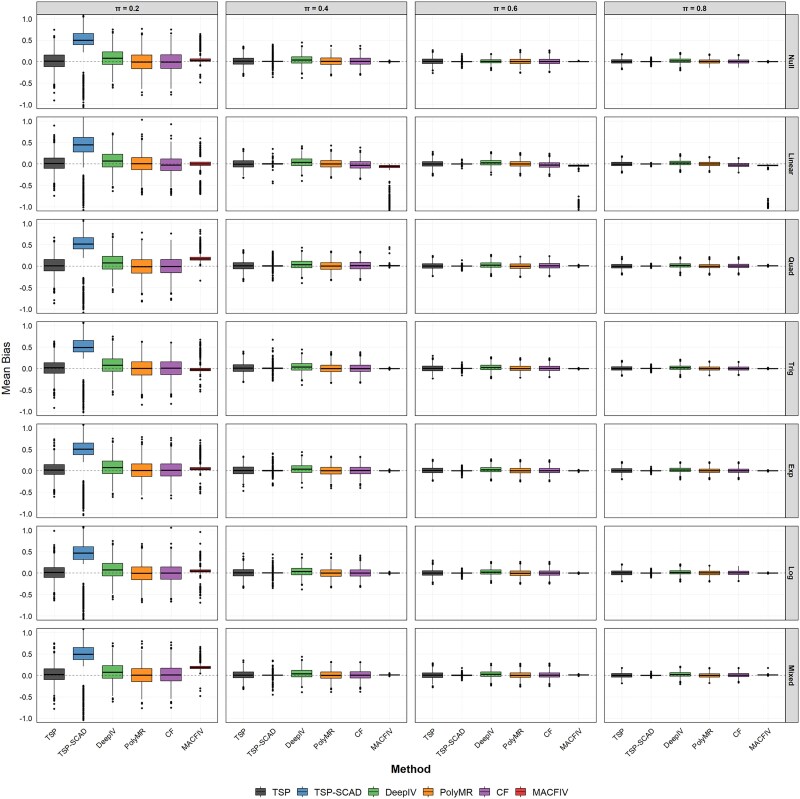
Boxplots of the estimation bias for the marginal effect function $f^{\prime }$ in Scenario 4: fix $n=2000, p=100, s=20$, start with $\boldsymbol{\gamma } = \left (\sqrt{\frac{2}{n}},\dots ,\sqrt{\frac{2}{n}}\right )$, then set the first $[\pi p]$ elements to follow a standard multivariate normal distribution to represent strong instruments, varying $\pi =0.2,0.4,0.6,0.8$.

Our simulations above are based on the “many weak” assumption. To better assess the robustness of the proposed method, we adjust the proportion of weak instruments in this setting. Specifically, we fix $n=2000, p=100$, and $s=20$, and based on the “many weak” setting with $\boldsymbol{\gamma } = \left (\frac{\mu }{\sqrt{n}},\dots ,\frac{\mu }{\sqrt{n}}\right )$, we further set the first $[\pi p]$ elements of $\boldsymbol{\gamma }$ to follow a standard multivariate normal distribution to represent strong instruments. We vary $\pi =0.2,0.4,0.6,0.8$ to adjust the proportion of strong and weak instruments. Figure 4 summarizes the mean bias results for the estimation of the marginal effect function in this scenario using boxplots, and the RMSE results are presented in Table 4. The MAE results summarized in Supplementary Section S2.

**Table 4:** Mean and standard deviation of RMSE results for estimating the marginal effect function $f^{\prime }$ in Scenario 4: fix $n=2000, p=100, s=20$, start with $\boldsymbol{\gamma } = \left (\sqrt{\frac{2}{n}},\dots ,\sqrt{\frac{2}{n}}\right )$, then set the first $[\pi p]$ elements to follow a standard multivariate normal distribution to represent strong instruments, varying $\pi =0.2,0.4,0.6,0.8$

		TSP		TSP-SCAD		DeepIV		PolyMR		CF		MACFIV	
	$f$	Mean	SD	Mean	SD	Mean	SD	Mean	SD	Mean	SD	Mean	SD
$\pi =0.2$	Null	1.389	0.683	0.160	0.147	0.246	0.110	0.359	0.189	0.088	0.035	0.018	0.008
	Linear	1.456	0.701	0.387	0.099	0.264	0.104	0.385	0.289	0.350	0.078	0.335	0.104
	Quad	1.393	0.686	0.177	0.151	0.249	0.109	0.359	0.251	0.099	0.031	0.054	0.010
	Trig	1.427	0.706	0.181	0.174	0.247	0.109	0.359	0.261	0.096	0.037	0.029	0.009
	Exp	1.391	0.719	0.162	0.136	0.249	0.109	0.367	0.199	0.089	0.037	0.020	0.008
	Log	1.368	0.703	0.168	0.186	0.248	0.109	0.369	0.250	0.088	0.034	0.025	0.006
	Mixed	1.384	0.679	0.172	0.142	0.251	0.108	0.357	0.242	0.101	0.030	0.061	0.013
$\pi =0.4$	Null	0.990	0.460	0.125	0.116	0.128	0.063	0.260	0.179	0.058	0.023	0.014	0.006
	Linear	1.069	0.452	0.355	0.084	0.160	0.058	0.260	0.156	0.326	0.074	0.324	0.106
	Quad	1.020	0.471	0.148	0.113	0.137	0.060	0.252	0.127	0.088	0.019	0.070	0.013
	Trig	0.989	0.473	0.129	0.129	0.131	0.062	0.258	0.152	0.061	0.023	0.019	0.006
	Exp	0.987	0.472	0.123	0.125	0.139	0.059	0.254	0.173	0.058	0.022	0.015	0.005
	Log	1.020	0.487	0.125	0.125	0.132	0.061	0.255	0.160	0.062	0.023	0.023	0.004
	Mixed	0.998	0.484	0.143	0.095	0.148	0.057	0.260	0.151	0.083	0.020	0.064	0.012
$\pi =0.6$	Null	0.833	0.376	0.107	0.109	0.090	0.042	0.209	0.123	0.047	0.018	0.011	0.004
	Linear	0.920	0.372	0.353	0.085	0.132	0.039	0.213	0.128	0.330	0.077	0.328	0.099
	Quad	0.824	0.391	0.143	0.094	0.104	0.038	0.204	0.113	0.093	0.016	0.083	0.015
	Trig	0.820	0.392	0.103	0.091	0.096	0.040	0.211	0.130	0.047	0.019	0.013	0.005
	Exp	0.847	0.384	0.108	0.109	0.116	0.037	0.207	0.139	0.049	0.018	0.015	0.005
	Log	0.817	0.389	0.109	0.109	0.095	0.040	0.212	0.157	0.050	0.017	0.022	0.003
	Mixed	0.821	0.383	0.131	0.077	0.131	0.035	0.206	0.107	0.086	0.017	0.075	0.014
$\pi =0.8$	Null	0.705	0.313	0.092	0.089	0.071	0.032	0.185	0.112	0.039	0.015	0.009	0.004
	Linear	0.808	0.309	0.342	0.081	0.119	0.029	0.190	0.124	0.321	0.075	0.327	0.117
	Quad	0.710	0.332	0.145	0.084	0.090	0.028	0.185	0.143	0.102	0.018	0.096	0.017
	Trig	0.712	0.320	0.091	0.094	0.080	0.030	0.187	0.112	0.040	0.015	0.011	0.003
	Exp	0.708	0.333	0.091	0.077	0.119	0.030	0.182	0.105	0.044	0.015	0.021	0.011
	Log	0.730	0.356	0.097	0.092	0.077	0.030	0.183	0.110	0.045	0.015	0.021	0.002
	Mixed	0.721	0.325	0.136	0.084	0.138	0.030	0.186	0.119	0.095	0.016	0.087	0.016

**Figure 5:**
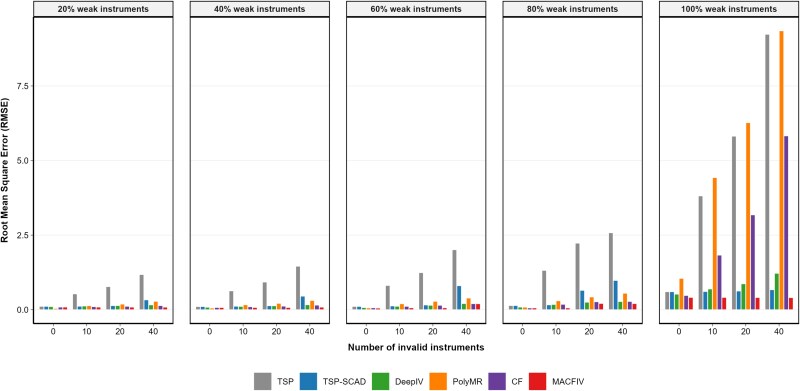
Grouped bar plot of the RMSE of the estimated marginal effect function $f^{\prime }(x)$ for TSP, TSP-SCAD, DeepIV, PolyMR, CF, and MACFIV methods under varying numbers of invalid instruments ($s=0,10,20,40$) and weak-instrument proportions (20%, 40%, 60%, 80%, 100%), with sample size, $n=2000$ number of instruments $p=100$, data generated from the mixed functional form $f(x)=\cos (0.1x)+\exp (0.1x)+0.01x^{2}$, and results based on 1000 replications.

In addition to the separate scenarios above, we further consider a joint setting where both the number of invalid instruments ($s$) and the proportion of strong instruments ($\pi $) vary simultaneously. Specifically, we fixed the sample size at $n = 2000$ and the number of instruments at $p = 100$, and generated the data under the mixed functional form $f(x)=\cos (0.1\cdot x) + \exp (0.1\cdot x) + 0.01\cdot x^{2}$ as an example. This extended design allows us to evaluate the robustness of different methods under more realistic conditions in which pleiotropy and weak instruments coexist. The corresponding results are summarized in Fig. 5.

**Figure 6:**
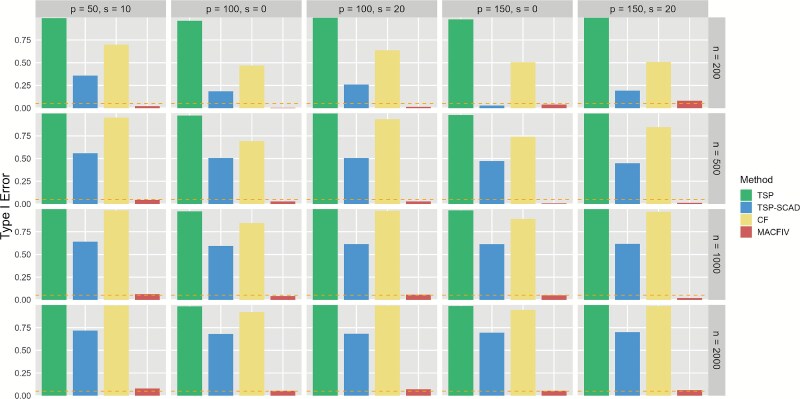
Bar plot of Type I error rates of TSP, TSP-SCAD, CF, and MACFIV method under the null hypothesis $H_{0}: f^{\prime }(x)=0$ in the “many weak” IV setting, with sample size $n=200,500,1000,2000$ and the number of IVs and invalid IVs $(p,s)=(50,10), (100,0), (100,20), (150,0), (150,20)$, based on 1000 replications, where the horizontal reference line indicates the nominal significance level of 0.05.

**Figure 7:**
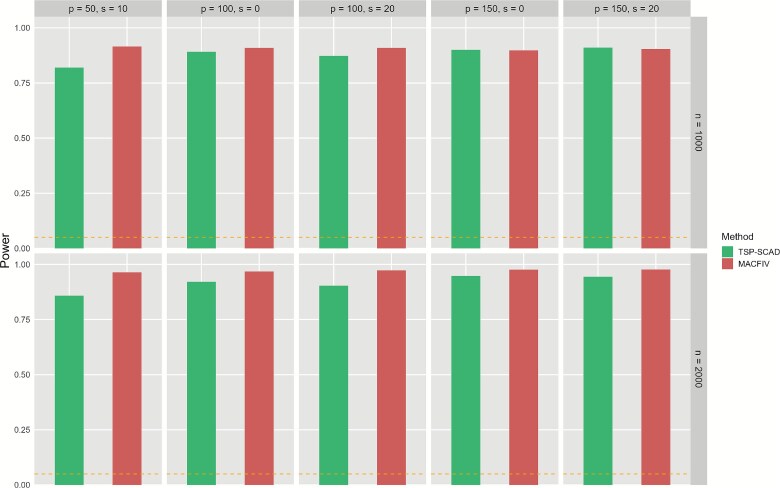
Bar plot of power of TSP-SCAD and MACFIV method under the alternative hypothesis $H_{1}: f^{\prime }(x)=-0.1\cdot \sin (0.1\cdot x) + 0.1\cdot \exp (0.1\cdot x) + 0.02\cdot x$ with $f(x)$ follows the mixed function form in the “many weak” IV setting, with sample size $n=1000,2000$ and the number of IVs and invalid IVs $(p,s)=(50,10), (100,0), (100,20), (150,0), (150,20)$, based on 1000 replications, where the horizontal reference line indicates the nominal significance level of 0.05.

To comprehensively assess both the validity and efficiency of the proposed MACFIV method, we also conduct hypothesis testing for the marginal effect function $f^{\prime }(x)$. Specifically, we test the null hypothesis $H_{0}: f^{\prime }(x)=0$ against the alternative hypothesis $H_{1}: f^{\prime }(x)\ne 0$. According to the asymptotic results of Theorem 1.1, under the null hypothesis, the test statistic $T(x)=\frac{\hat{f}^{\prime }(x)}{\text{SE}(\hat{f}^{\prime }(x))}$ follows a standard normal distribution when the sample size is sufficiently large, where $\hat{f}^{\prime }(x)$ is the estimated marginal effect and $\text{SE}(\hat{f}^{\prime }(x))$ is its standard error. We reject $H_{0}$ at significance level $\alpha =0.05$ if the $P$-value is $<.05$. To evaluate the validity of MACFIV, we generate data under the null hypothesis, where $f(x)=0$ and $f^{\prime }(x)=0$, corresponding to the previously mentioned case where the functional form is null. To evaluate the efficiency, we generate data under the alternative hypothesis, using the functional form of the mixed function $f(x)=\cos (0.1\cdot x) + \exp (0.1\cdot x) + 0.01\cdot x^{2}$ as an example of the data generation mechanism, corresponding to a nonzero marginal effect function. We compute the proportion of the null hypothesis $H_{0}$ that is rejected in the simulation, representing the Type I error rate and the power, respectively. We calculate these metrics under different $(n, p, s)$ parameter settings and various forms of marginal effect functions and compare them with other methods. Since the high bias in the TSP and CF methods may lead to an inflated power, we only compare power with the TSP-SCAD method. DeepIV, which mainly focuses on prediction and does not provide formal statistical testing procedures, is therefore not included in the hypothesis testing comparison. This also highlights an innovation of MACFIV, as it combines flexible nonlinear estimation with valid statistical inference. The specific results are presented in Figs 6 and 7.

## Real data application

In the real data application, we focus on studying the causal relationship between BMI and blood pressure. While many studies have explored the observational relationship between BMI and blood pressure [47, 48], determining whether this relationship is nonlinear remains challenging. Moreover, recent studies by Staley and Burgess [49], Sulc *et al.* [42], and Chen *et al.* [50] suggest that a nonlinear causal relationship exists between BMI and blood pressure, but in general, only a few studies have explored the specific shape of the causal relationship. We believe that the shape of causality is important in practical applications, so our analysis leverages data from the Atherosclerosis Risk in Communities (ARIC) study to examine the causal relationship between BMI and blood pressure and to characterize its shape using the marginal effect function.

The ARIC study is a long-term cohort investigation that began in 1987 and continues to collect data. Over the initial recruitment period (1987–89), 15 792 individuals aged 45–64 years were enrolled from four distinct regions across the United States. Comprehensive health assessments, along with biological and genetic data collection, were performed during each follow-up phase.

## Results

### Simulations: better performance of MACFIV over other methods

We evaluate the performance and robustness of the five methods under different scenarios using the mean and standard deviation of RMSE and MAE calculated from 1000 simulations. Figure 1 illustrates the bias patterns across all settings and shows that our estimator produces lower and more stable mean bias, while methods such as DeepIV, PolyMR, and CF exhibit increasing and highly variable bias as the proportion of invalid instruments rises. The results in Table 1 and Supplementary Table S2 also indicate that when there are no invalid instruments, our method demonstrates superior estimation performance compared to the methods used for comparison, even in the presence of numerous weak instruments. This highlights the robustness of the model averaging approach to weak instruments. When invalid instruments are present, the methods used for comparison, except for TSP-SCAD, fail to adequately address invalid instruments, leading to unstable estimates that deteriorate as the proportion of invalid instruments increases. In contrast, our method maintains relatively stable results under these conditions and outperforms TSP-SCAD in terms of estimation accuracy.

Figure 2 shows that MACFIV achieves consistently low mean bias across all sample sizes and data-generating mechanisms, with boxplots tightly centered around zero. In contrast, several competing methods exhibit noticeably larger or more variable biases. This visual evidence highlights the superior finite-sample stability of MACFIV. The results from Table 2 and Supplementary Table S3 show that as the sample size increases, the RMSE and MAE of MACFIV decrease steadily in both mean and variance. The relatively poorer performance in small samples is largely due to the greater estimation uncertainty of the first-stage model-averaged control function, which propagates to the second stage and inflates bias. As the sample size grows, this propagated uncertainty diminishes, and the empirical performance approaches the method’s asymptotic properties. Even at sample sizes comparable with large-scale GWAS, MACFIV continues to provide accurate estimates, indicating suitability for biobank-scale applications. Additionally, it is worth noting that under the “many weak” design, the instruments become progressively weaker as the sample size grows, which explains the worsening performance of the TSP and CF methods. In contrast, our proposed method maintains robust performance even with weaker instruments and achieves more accurate estimates than other methods, further validating its asymptotic properties as the sample size increases.

Figure 3 illustrates that the MACFIV estimates remain tightly concentrated around zero across all instrument settings, with smaller spread compared with the competing methods. The dispersion of the other methods increases when the number of instruments is small, and although it narrows as the instrument count grows, their variability remains substantially larger than that of MACFIV. Table 3 and Supplementary Table S4 present the corresponding numerical results. The results show that across different instrument settings, the MACFIV method consistently maintains the smallest mean and variance. As the number of instruments increases, the mean error of the MACFIV method increases slightly but remains small, whereas the mean errors of other methods gradually decrease from larger initial values. This may be attributed to the decreasing proportion of invalid instruments as the total number of instruments increases, leading to a reduction in bias caused by invalid instruments in other methods.

Figure 4 together with Table 4 and Supplementary Table S5 present the performance comparison of various methods after adjusting the proportion of strong and weak instruments. As stronger instruments are introduced, the bias caused by weak instruments is significantly reduced, especially for methods that are more sensitive to weak instruments. Overall, as the proportion of strong instruments increases, the mean and variance of the errors for all methods decrease. The MACFIV method continues to show better performance in most cases, while other methods also achieve reasonable estimation accuracy. This highlights the significant impact of weak instruments on model estimation. In real-world data, where all instruments may be weak, other methods can result in large estimation errors, showing the importance of improving methods to handle weak instruments effectively.

In addition to the results reported for each scenario, we also observed a common pattern in the linear settings across all simulations. When the true structural function is linear, the marginal effect $f^{\prime }(x)$ is constant, and any remaining correlation between the exposure and the estimation error of the control function can shift the estimated slope in the same direction. This residual correlation may arise not only from sampling variability but also from small amounts of horizontal pleiotropy that are not fully removed in finite samples. Since the second stage uses smoothing to estimate this constant effect, this remaining variation can lead to a slight upward shift in the estimated value. The phenomenon is more visible when the instruments are weak, because the control function is then estimated with greater uncertainty and the influence of horizontal pleiotropy becomes relatively more pronounced. In contrast, when the structural relationship is nonlinear, the marginal effect varies with the exposure level. The smoothing step then acts locally, and the remaining variation from the first stage is distributed across different regions of the curve rather than moving the entire function in one direction. This leads to more stable estimates in the nonlinear settings. These observations suggest that the small inflation seen in the linear settings is caused by finite sample variation in the control function combined with mild horizontal pleiotropy, and does not reflect a limitation of the proposed method.

To provide a more comprehensive view, Fig. 5 displays the RMSE of all six methods across different combinations of invalid instruments and weak-instrument proportions. The grouped bar plots clearly show that as the proportion of weak instruments increases, the estimation errors of competing methods rise sharply, and the presence of additional invalid instruments further amplifies this deterioration. In contrast, MACFIV remains stable with consistently low RMSE across all settings. The difference is particularly striking under the most challenging case with 100% weak instruments and a large number of invalid instruments ($s = 40$), where the competing methods fail severely while MACFIV continues to deliver accurate estimates. These results further highlight the robustness of MACFIV in realistic scenarios where weak instruments and pleiotropy occur jointly.

For the validity and efficiency of our proposed method, as shown in Fig. 6, our method maintains the Type I error rate close to the nominal level (5%) in most cases under the null hypothesis, whereas other methods fail to control it at the nominal level. This demonstrates the validity of our approach. Furthermore, as shown in Fig. 7, our method consistently maintains a high power ($>0.8$) and achieves an improvement in power compared with the TSP-SCAD method in most cases, confirming the higher efficiency of our approach.

Overall, in most of the considered scenarios, the proposed method demonstrates better performance. This can be attributed to two key factors: first, the robustness of the model averaging approach in the presence of weak instruments; and second, the SCAD-penalized regression effectively addresses invalid instruments. Consequently, the estimation bias introduced by these two types of instruments is mitigated within our framework, enabling high stability in complex instrument settings along with good asymptotic properties.

### Real data analysis: the causal relationships between body mass index and blood pressure

**Figure 8:**
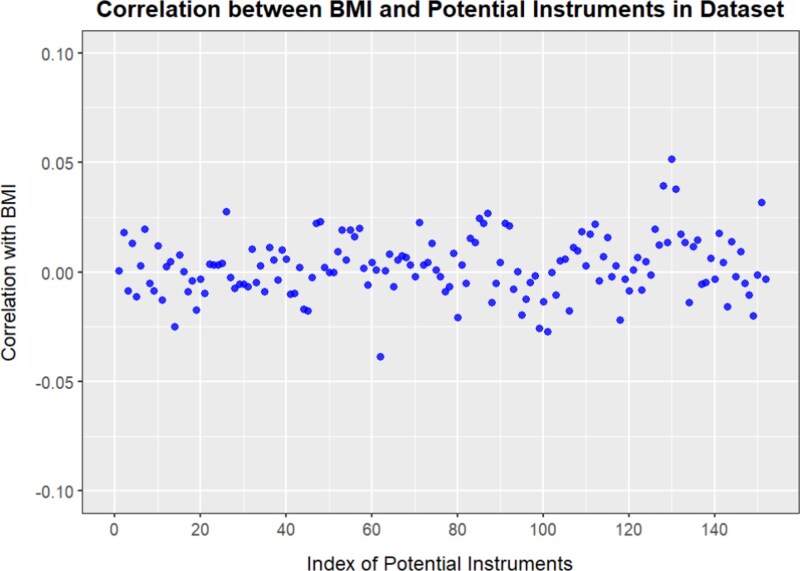
The Pearson correlation between the 152 SNP instruments and BMI.

In this section, we use the ARIC data recently analyzed by Chen *et al.* [50] to study the relationship between BMI and blood pressure, which includes BMI, systolic blood pressure (SBP), and diastolic blood pressure (DBP) measurements for a total of 8734 individuals, along with 152 SNPs associated with BMI. Figure 8 shows the sample Pearson correlations between all SNPs and BMI. It can be observed from the figure that the correlations between the SNPs and BMI are weak, as the sample correlation coefficients are small, indicating that they are all weak instruments. We treat BMI as the exposure and SBP/DBP as the outcomes, applying our model to conduct a nonlinear Mendelian randomization analysis.

**Figure 9:**
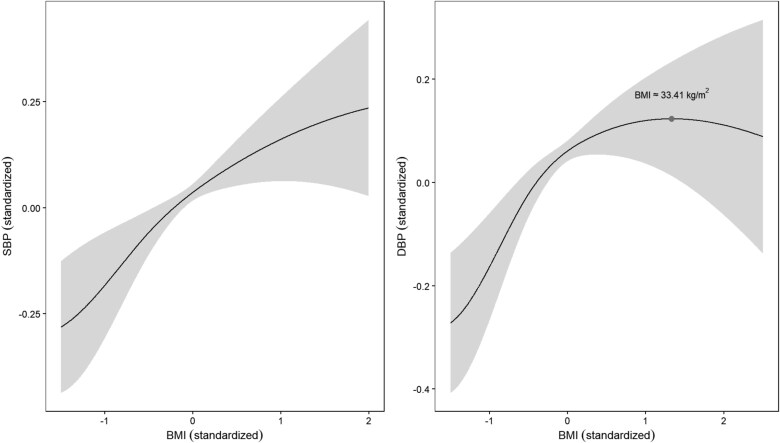
Causal relationship between BMI and SBP/DBP, with the shaded areas representing the 95% confidence intervals.

We first standardized the data for BMI, SBP, and DBP. To apply the MACFIV method, we follow the same procedure for hyperparameter selection as described in the Simulations section. Specifically, we use the BIC to tune $\lambda $ and set $a=3.7$, as recommended by Fan and Li [45]. Using the MACFIV method, we estimated the causal relationship between BMI and SBP/DBP. Figure 9 presents the causal relationship between BMI and SBP/DBP. It can be observed that SBP increases with BMI, with a gradually decreasing slope, indicating a slight nonlinearity. In contrast, the causal relationship between BMI and DBP exhibits strong nonlinearity, showing a critical point (BMI $\approx 33.41$  $\text{kg}/\text{m}^{2}$), before which DBP increases with increasing BMI, and after which the opposite conclusion is presented, with DBP decreasing with increasing BMI.

**Figure 10:**
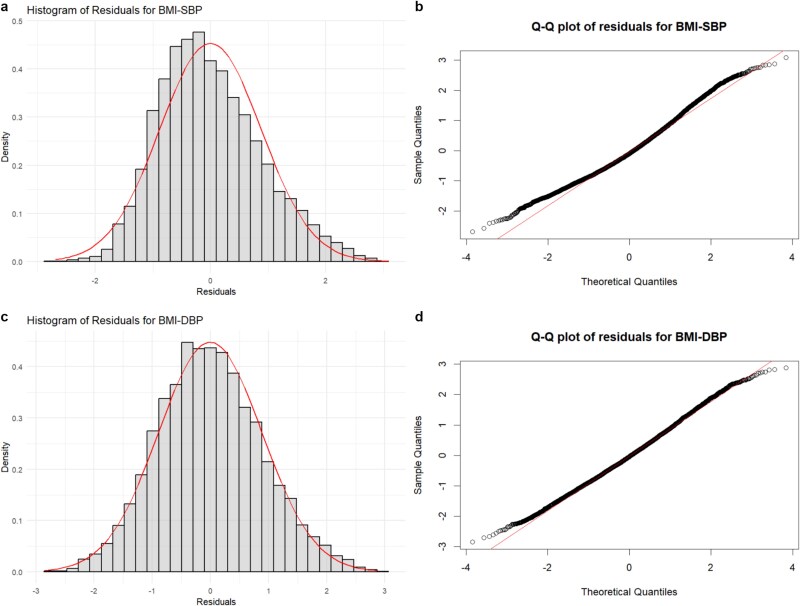
Residual analysis for the BMI–SBP and BMI–DBP relationships: (a) histogram of residuals for BMI–SBP; (b) Q-Q plot of residuals for BMI–SBP; (c) histogram of residuals for BMI–DBP; and (d) Q-Q plot of residuals for BMI–DBP.

To assess the adequacy of the B-spline approximation, we conduct residual analysis for both the BMI–SBP and BMI–DBP relationships. Figure 10 shows the histograms and Q-Q plots of the residuals for each outcome. The histograms (Figs 10a and 6c) indicate that the residuals are approximately symmetrically distributed around zero. This suggests that the MACFIV method adequately captures the underlying nonlinear trends in both relationships. The Q-Q plots (Figs 10b and 6d) further support the validity of the smoothness assumptions, as the residuals closely follow the theoretical distribution line. These results demonstrate that the MACFIV method is appropriate for modeling the nonlinear relationships between BMI and hypertension. In addition, to examine whether the turning point in the BMI–DBP curve is sensitive to the smoothing specification, we re-estimate the curve using different B-spline basis numbers ($m\in \{4,5,6\}$) with both quantile-based and evenly spaced knots, as well as kernel smoothing and local polynomial regression. Across all methods, the turning point estimates range from 31.76 to 34.34 $\text{kg}/\text{m}^{2}$, with our default setting ($m=5$, uniform knots) yielding 33.41 $\text{kg}/\text{m}^{2}$, suggesting that the result is robust to the choice of smoothing method. The detailed methods and results are provided in Supplementary Section S2. Given that the estimated turning point remains essentially unchanged across a variety of spline specifications, it is natural to consider how spline complexity is typically chosen in practice. A common strategy is to begin with a moderate number of basis functions and increase flexibility only when the fitted curve shows signs of under-smoothing, while information criteria such as AIC or BIC are frequently used as additional guidance. In our application, the stability of the turning point across these choices indicates that the selected spline specification is adequate.

In our real data analysis, the MACFIV method reveals a potential nonlinear causal relationship between BMI and SBP/DBP, confirming the findings of Chen *et al.* [50] and providing a specific causal relationship shape, which is valuable for practical applications. Furthermore, this conclusion aligns with the results of Staley and Burgess [49], validating the effectiveness of our proposed method in real data. This non-monotonic pattern may suggest that in severely obese individuals, further BMI increases are not accompanied by proportional increases in diastolic blood pressure. Possible physiological explanations include obesity-related vascular remodeling, reduced peripheral vascular resistance, or altered autonomic regulation, all of which could limit the rise in DBP despite continued weight gain. From a weight management perspective, these findings imply that for hypertensive patients whose BMI exceeds this threshold, weight reduction targets may need to be individualized. While current guidelines emphasize weight loss as a primary strategy for blood pressure control, the marginal DBP-lowering benefit of further weight loss could be smaller in this subgroup. Consequently, clinical decision-making should balance blood pressure reduction goals with broader metabolic and cardiovascular risk factors, rather than focusing solely on DBP. In terms of antihypertensive therapy, the decline in DBP at very high BMI levels may reflect a shift in the dominant pathophysiological mechanisms underlying hypertension, with the primary driver changing from vascular tone-mediated elevation to hypertension driven more by volume overload or increased cardiac output. This shift could reduce the relative efficacy of medications primarily targeting vascular tone (such as calcium channel blockers and $\alpha $-blockers), while increasing the potential relevance of diuretics or agents acting on the renin–angiotensin–aldosterone system. Taken together, these results suggest the existence of a distinct subgroup of obese hypertensive patients with BMI above $\sim 33$–34 $\text{kg}/\text{m}^{2}$, who may benefit from tailored BP monitoring schedules and potentially different therapeutic priorities. Further clinical studies are needed to confirm these observations and to translate them into specific treatment guidelines.

## Discussion

In this paper, we propose a new IV regression method based on model averaging and control function to estimate the marginal effect function, particularly for estimating the shape of a nonlinear causal relationship. When dealing with larger and more complex sets of instruments, the model averaging approach integrates different subsets of genetic instruments, helping to reduce bias arising from model misspecification. This is because any single first-stage model may include instruments that have very weak effects or may omit instruments that carry useful signal. By combining several nested models, each using a different group of instruments, the final estimate does not rely on one specific model and is less affected by an incorrect choice of instruments. This leads to a more stable estimate of the exposure, which is important for the control function in the second stage. This approach is also suitable in situations where many or even all of the genetic instruments are weak. To address pleiotropy, we use the SCAD penalty, which effectively identifies invalid instruments, thus reducing bias introduced by them. Unlike most existing methods that require the prior exclusion of weak instruments, our approach leverages the valid information from all genetic instruments, providing relatively accurate estimates of the causal relationship shape. Theoretical results indicate that the estimates provided by our method have favorable asymptotic properties. We compare the performance of MACFIV with several alternative methods under various scenarios. Our simulations demonstrate that MACFIV achieves a better balance between bias and variance compared with other methods, particularly in the presence of weak and invalid instruments. For instance, when the proportion of invalid instruments increased, MACFIV maintained relatively low bias while controlling variance, whereas other methods exhibited significant bias or increased variance. This balance is achieved through the model averaging approach in the first stage, which reduces bias from weak instruments, and the SCAD penalization in the second stage, which mitigates the impact of invalid instruments. In large-scale GWAS settings with sample sizes comparable to those of contemporary biobank studies, MACFIV is expected to remain both robust and computationally practical. Our simulations at such scales indicate that the estimation accuracy continues to improve as the sample size grows, because the uncertainty from the first-stage control function estimation diminishes and the empirical performance aligns more closely with the method’s asymptotic properties. In terms of computational feasibility, while runtime naturally increases with larger datasets, the procedure remains tractable for single-sample analyses and can be further accelerated through parallel computation. These results suggest that MACFIV can be readily applied to biobank-scale Mendelian randomization studies without compromising accuracy or interpretability. However, we also acknowledge that MACFIV may exhibit slightly higher computational complexity in certain settings, which could be a limitation in large-scale applications. Overall, MACFIV provides a robust framework for causal inference in complex scenarios with many weak and invalid instruments.

Several recent Mendelian randomization methods, such as MR-Egger [51], the weighted median estimator [52], MR-PRESSO [53], and CAUSE [54] have been proposed to mitigate bias from pleiotropic instruments. These approaches are primarily designed for linear exposure–outcome models and typically rely on summary-level data, achieving robustness through bias-resistant estimation, median aggregation, outlier correction, or Bayesian modeling of invalid instruments. MACFIV complements these approaches by targeting individual-level data and accommodating nonlinear exposure–outcome relationships through the integration of control functions and SCAD penalization. Conceptually, these methods all address pleiotropy but from different angles: MR-Egger and weighted median focus on robustness through alternative identification assumptions, MR-PRESSO detects and corrects for horizontal pleiotropic outliers, CAUSE leverages genome-wide summary statistics to jointly model correlated pleiotropy, while MACFIV uses first-stage model averaging to stabilize weak-instrument prediction and second-stage penalization to directly detect invalid instruments. Together, these approaches enrich the methodological toolkit for Mendelian randomization, covering a spectrum of data types, modeling assumptions, and inferential goals.

Beyond MR-specific approaches, recent deep learning-based IV frameworks provide powerful tools for flexible nonlinear modeling but are primarily oriented toward predictive tasks rather than formal causal inference. While such neural network-driven methods can capture highly complex functional relationships, they generally lack procedures for hypothesis testing or uncertainty quantification of causal effects, and their computational cost is often considerable due to intensive model training and hyperparameter optimization. In contrast, MACFIV emphasizes statistical interpretability and causal testability while maintaining computational efficiency through its combination of model averaging and penalized correction. This balance enables MACFIV to deliver stable, interpretable, and scalable causal estimates, making it well suited for large-scale biomedical studies where nonlinear exposure–outcome relationships coexist with imperfect instruments and transparent inference is essential for scientific interpretation.

The nonlinear causal relationship between BMI and hypertension revealed in our case study has important clinical and biological implications. Our analysis reveals a nonlinear causal relationship between BMI and hypertension. The observed nonlinear relationship between BMI and blood pressure aligns with established physiological mechanisms [55, 56]. The increase in SBP with BMI may be attributed to increased cardiac output and arterial stiffness, which progressively worsen with obesity. In contrast, DBP appears to plateau beyond BMI $\approx 33.41$  $\text{kg}/\text{m}^{2}$, suggesting a possible compensatory mechanism or saturation effect. This threshold-like behavior may reflect adaptive responses such as increased vascular compliance at extreme BMI levels. Our findings emphasize the importance of considering nonlinear causal effects in epidemiological studies, as traditional linear models may underestimate risks at higher BMI levels.

In addition, two practical issues deserve attention. The first concerns the possibility that the relationship between the exposure and the instruments is not perfectly linear. Our method uses a linear first stage, which is the standard choice in Mendelian Randomization and usually provides a good approximation. If the true relationship contains nonlinear components, the linear first stage may add extra variation to the control function residuals. This may reduce efficiency but does not create clear bias unless the nonlinearity is very strong. Future extensions of MACFIV could use more flexible first-stage models, such as nonparametric regression, provided that the required identifiability conditions are satisfied. The second issue concerns correlations among instruments. Genetic variants often show correlation, which reduces the effective amount of independent information but does not violate the assumptions of the method. The model averaging step helps to stabilize the first stage by combining several nested models rather than depending on one chosen model. This makes the procedure less sensitive to correlation among instruments. Our supplementary simulations, where instruments follow an autoregressive correlation pattern with moderate or strong dependence, show that MACFIV continues to outperform the comparison methods. These findings suggest that MACFIV remains reliable in realistic settings where instruments are correlated.

Moreover, our method also has some limitations and areas for further research. First, MACFIV is developed for individual-level data and is not directly applicable to summary-level data. Although individual-level data provide greater flexibility for modeling, they are often difficult to obtain. Extending MACFIV to summary-level data presents both conceptual and technical challenges. In particular, the control function approach relies on sample-level residuals and the joint covariance structure among instruments, exposure, and outcome, which cannot be recovered from marginal summary statistics. As a result, the nonlinear adjustment terms in MACFIV become unidentified without individual-level variation. Furthermore, penalization methods such as SCAD and the model averaging procedure require likelihood-based selection across candidate models, which cannot be replicated using aggregated regression coefficients. These obstacles make a direct summary-data implementation nontrivial and highlight the need for new identification strategies tailored to nonlinear instrument settings. Addressing these challenges in a nonlinear framework remains an important direction for future research. Second, many studies in the nonlinear framework often incorporate the nonlinearity between exposure and instruments, including dimensionality reduction model [7] and additive model [13], to investigate the potential nonlinear effects of instruments on exposure. We believe that, under the condition of ensuring model identifiability, considering nonlinearity in both stages is a direction worth further exploration. In addition, the number and form of spline basis functions have a significant impact on the nonlinear function fitting process, and determining appropriate spline settings for different scenarios remains a challenge. The flexibility of B-splines, while advantageous for capturing complex nonlinear relationships, may lead to overfitting in simpler settings, such as when the true relationship is linear. This overfitting can result in increased estimation error, particularly in the presence of weak instruments or when the model is misspecified. In addition, spline estimators may become less stable near the boundaries of the exposure distribution because fewer observations fall in these regions. To improve stability in our implementation, we place interior knots only and avoid placing knots too close to the boundaries, and the control function is smoothed before entering the second stage to further reduce variation at the edges. Therefore, careful selection of the number and form of spline basis functions is crucial to balance flexibility and overfitting, especially when the true relationship is unknown or may vary across scenarios. Furthermore, using model averaging based on nonlinearity in the first stage is another potential extension to consider. Although Chen *et al.* [16] have explored this, there may be some potential challenges in the framework where both stages are nonlinear. From the perspective of statistical testing, developing specific test statistics for assessing nonlinearity in a nonlinear framework also remains a challenge. While it is possible to test for local nonlinearity at specific sample points, testing the overall nonlinearity of the function is difficult. The local nature of spline functions results in potential correlations between the second derivatives at different sample points, complicating the task of synthesizing these local tests into a global statistic for evaluating overall nonlinearity. Further research is needed to address this issue and establish appropriate global test statistics for nonlinearity. Finally, when the number of genetic instruments is large, some exogenous variables may be spuriously correlated with some instruments due to high dimensionality, and correlation methods can be developed to address this issue. These remain for future research.

**Key Points**

- The presence of nonlinear causal relationships may distort the conclusions of traditional linear causal inference, while the existence of weak and invalid instrumental variables (IVs) can bias causal reasoning.
- We proposed a model-averaged control function-based instrumental variable regression (MACFIV) framework that can identify nonlinear causal relationships while being robust to weak associations and invalid IVs.
- The performance of our proposed MACFIV is validated through extensive simulation studies and real data analysis, revealing a potential nonlinear causal relationship between BMI and hypertension.

## Supplementary Material

Supplementary_Materials_bbaf714

## Data Availability

The R codes used for the simulations and real analysis in this article are available in the website: https://github.com/YQHuFD/MACFIV.
