# Supplementary material for: MACFIV: a novel framework for nonlinear causal inference in the body mass index–hypertension relationship with many weak and pleiotropic genetic instruments
Source: Brief Bioinform. 2026 Jan 11;27(1):bbaf714. doi: 10.1093/bib/bbaf714 (PMC12790626; doi:10.1093/bib/bbaf714)
Supplement: Supplementary_Materials_bbaf714 [file supplementary_materials_bbaf714.pdf]

Supplementary Materials for "MACFIV: A Novel Framework for Nonlinear Causal Inference in BMI-Hypertension Relationship with Many Weak and Pleiotropic Genetic Instruments"

Dong Chen[1], Yuquan Wang[1], Dapeng Shi[2], Yunlong Cao[1], and Yue-Qing Hu[1,2,*]

[1]State Key Laboratory of Genetics and Development of Complex Phenotypes, Institute of Biostatistics, School of Life Sciences, Fudan University, Shanghai, China

[2]Shanghai Center for Mathematical Sciences, Fudan University, Shanghai, China

[*]*email*: yuehu@fudan.edu.cn

Contents

S1: Proof of Theorem 1 . . . **3**

S2: Additional Demonstrations . . . **7**

1. Extension to nonlinear control function . . . 7
2. Methodology for the binary outcome extension . . . 8

S3: Additional Simulation and Empirical Results . . . **10**

1. The F-statistic of instruments strength . . . 10
2. Simulation results using categorical instrumental variables . . . 10
3. Simulation results using continuous instrumental variables . . . 15
4. Computational time of MACFIV and other methods . . . 28
5. Sensitivity analysis of MACFIV to initial instrument ordering in the first stage . . . 29
6. Joint tuning of B-spline number and SCAD parameter . . . 31
7. Sensitivity of MACFIV to pleiotropic effect magnitude . . . 33

8. Simulation results for the extended algorithm with nonlinear control functions . . . 35
9. Simulation results for the binary outcome extension of MACFIV 37
10. Robustness checks for the nonlinear BMI–DBP relationship 40

S1: Proof of Theorem 1

We first provide the following assumptions.

Assumption 1. Suppose that the data $\mathcal{D} = \{\boldsymbol{G}, \boldsymbol{X}, \boldsymbol{Y}\}$ is independently and identically distributed and x has finite second moments.

Assumption 2. Assume that $\mathbb{E}[u \mid v, \boldsymbol{g}] = \mathbb{E}[u \mid v]$, and $\mathbb{E}[v \mid \boldsymbol{g}] = 0$. Furthermore, assume that $u = \rho v + e$, with $\mathbb{E}[e \mid \boldsymbol{g}, v] = \mathbb{E}[e \mid v] = 0$. $\sigma_v^2 = \mathbb{E}\left[v^2 \mid \boldsymbol{g}\right]$ and $\sigma_e^2 = \mathbb{E}\left[e^2 \mid \boldsymbol{g}\right]$ are assumed to be positive constants, and assume that $\mathbb{E}\left[e^4 \mid \boldsymbol{g}, v\right]$ is bounded by some positive constant independent of n and p.

Assumption 3. $\boldsymbol{\gamma} = \boldsymbol{\gamma}_n = \boldsymbol{\delta}/\sqrt{n}$, where $\boldsymbol{\delta}$ is an unknown constant vector.

Assumption 4. As $n \to \infty$, $n^{-1}\boldsymbol{G}^T\boldsymbol{G} \xrightarrow{p} \boldsymbol{L}$ and $n^{-1/2}\boldsymbol{G}^T\boldsymbol{v} \xrightarrow{d} \boldsymbol{R} \sim \mathcal{N}(\boldsymbol{0}, \boldsymbol{\Omega})$, where $\boldsymbol{L} = \mathbb{E}\left[\boldsymbol{g}\boldsymbol{g}^T\right]$ is a finite and full-rank matrix, and $\boldsymbol{\Omega} = \mathbb{E}\left[\boldsymbol{g}\boldsymbol{g}^T v^2\right]$.

Assumption 5. Denote $\lambda_{\min}(A)$ and $\lambda_{\max}(A)$ as the minimum and maximum eigenvalues of a positive definite matrix A, respectively. Define $\boldsymbol{S} = (\mathcal{B}, \boldsymbol{G}, \boldsymbol{v})$. Assume that

$$\lambda_1 \leq \lambda_{\min}\left(\frac{1}{n}\boldsymbol{S}^T\boldsymbol{S}\right) \leq \lambda_{\max}\left(\frac{1}{n}\boldsymbol{S}^T\boldsymbol{S}\right) \leq \lambda_2$$

where λ_1 and λ_2 are two positive constants.

Assumption 6. The number of invalid instruments $s = |\mathcal{A}_I|$ is less than $p/2$.

Assumption 7. The support of x is $[a, b]$, where a and b are finite real number. Additionally, the marginal density function of x is bounded below by $c > 0$ and above by $C > 0$.

Assumption 8. Suppose that $f(\cdot) \in \mathcal{H}_{[a,b]}(\theta, L)$ for some $\theta \geq 2$, where $\mathcal{H}_{[a,b]}(\theta, L)$ is the set of θ-times differentiable functions $f : [a, b] \to \mathbb{R}$ such that its derivative $f^{(l)}$ with $l = \lfloor \theta \rfloor$ satisfies:

$$|f^{(l)}(t_1) - f^{(l)}(t_2)| \leq L|t_1 - t_2|^{\theta - l}, \text{ for any } t_1, t_2 \in [a, b] \text{ and a constant } L > 0.$$

Remark. Assumption 1 is a mild restriction on the instruments to ensure large sample properties. Assumption 2 guarantees the identifiability of the control function and the correctness of the model, which is commonly used in control function methods [1, 2]. Assumptions 3 and 4 ensure the asymptotic properties of the model averaging method, as discussed in model averaging theory [3, 4, 5]. Assumption 3 requires that all instruments have a relatively weak association with the exposure, which is also the definition of weak instruments. This reflects a common characteristic and intuitively explains why model averaging methods can handle weak

instrument scenarios. Assumption 5 guarantees the asymptotic properties of the SCAD penalty, especially when using control function estimates as substitutes, ensuring that they do not differ significantly from the original form. Assumption 6 is the commonly used majority rule, which can be extended from the linear framework to the nonlinear framework. Assumptions 7 and 8 pertain to the approximation error of B-splines, commonly used in nonparametric methods with B-spline approximations [1, 6, 7]. These assumptions ensure that the approximation error of both the original function and its derivatives is sufficiently small, thereby enabling the provision of accurate estimates.

Under the assumptions mentioned above, we now gradually provide the asymptotic properties of the marginal effect function.

Lemma 1. Suppose that Assumptions 3–4 hold. As $n \to \infty$, we have

$$\mathcal{C}_n(\boldsymbol{w}) \xrightarrow{d} \boldsymbol{w}^T \boldsymbol{\zeta} \boldsymbol{w} + 2\sigma^2 \boldsymbol{K}^T \boldsymbol{w},$$

where $\boldsymbol{\zeta}$ is a $Q \times Q$ matrix with the (i,j)th element $\zeta_{i,j} = \boldsymbol{R}_i^T \boldsymbol{L} \boldsymbol{R}_j$ and $\boldsymbol{R}_i = \boldsymbol{C}_i \boldsymbol{\delta} + (\boldsymbol{P}_i - \boldsymbol{L}^{-1})\boldsymbol{R}$, $\boldsymbol{C}_i = \boldsymbol{P}_i \boldsymbol{L} - \boldsymbol{I}_p$, $\boldsymbol{P}_i = \boldsymbol{\Pi}_i(\boldsymbol{\Pi}_i^T \boldsymbol{L} \boldsymbol{\Pi}_i)^{-1}\boldsymbol{\Pi}_i^T$, and $\sigma^2 = \mathbb{E}\left[v^2\right]$, $\boldsymbol{K} = (K_1, \ldots, K_Q)^T$. Also, we have

$$\hat{\boldsymbol{w}} \xrightarrow{d} \boldsymbol{w}^* = \underset{\boldsymbol{w} \in \mathcal{H}_Q}{\arg\min} \left(\boldsymbol{w}^T \boldsymbol{\zeta} \boldsymbol{w} + 2\sigma^2 \boldsymbol{K}^T \boldsymbol{w}\right),$$

and

$$\|\hat{\boldsymbol{\gamma}}(\hat{\boldsymbol{w}}) - \boldsymbol{\gamma}\| = o_p(1).$$

Proof. See Liu [5], Proof of Theorem 4. □

Corollary 1. The residuals of the model averaging estimate satisfy

$$\|\hat{\boldsymbol{v}} - \boldsymbol{v}\| = o_p(1).$$

Proof. It is sufficient to note that

$$\|\hat{\boldsymbol{v}} - \boldsymbol{v}\| = \|(\boldsymbol{X} - \boldsymbol{G}\hat{\boldsymbol{\gamma}}) - (\boldsymbol{X} - \boldsymbol{G}\boldsymbol{\gamma})\| = \|\boldsymbol{G}(\hat{\boldsymbol{\gamma}} - \boldsymbol{\gamma})\| = o_p(1).$$ □

Lemma 2. Suppose that $\lambda_n \to 0$ and $\sqrt{n}\lambda_n \to \infty$ as $n \to \infty$, under Assumptions 1-6, we have

1. (Consistency in variable selection) $\lim_{n\to\infty} P\left(\hat{\mathcal{A}}_I = \mathcal{A}_I\right) = 1.$

2. (Consistency of $\hat{\boldsymbol{\beta}}$) $\|\hat{\boldsymbol{\beta}} - \boldsymbol{\beta}\| = o_p(1)$.

3. (Asymptotic normality) $\sqrt{n}\left(\hat{\boldsymbol{\beta}} - \boldsymbol{\beta}\right) \xrightarrow{d} \mathcal{N}(\mathbf{0}, \boldsymbol{U})$, where $\boldsymbol{U} = [\boldsymbol{H}^{-1}\boldsymbol{V}\boldsymbol{H}^{-1}]_{\boldsymbol{\beta}\boldsymbol{\beta}}$. In the above expression, $\boldsymbol{H} = \mathbb{E}\left[\nabla^2_{\boldsymbol{\theta}} \ell(y, x, \boldsymbol{g}, v; \boldsymbol{\theta})\right]$ with $\boldsymbol{\theta} = (\boldsymbol{\beta}^T, \boldsymbol{\alpha}^T, \rho)^T$ and

$$\ell(y, x, \boldsymbol{g}, v; \boldsymbol{\theta}) = \frac{1}{2}(y - \boldsymbol{B}^T\boldsymbol{\beta} - \boldsymbol{g}^T\boldsymbol{\alpha} - \rho v)^2 + p_\lambda^{\text{SCAD}}(\boldsymbol{\alpha}).$$

Additionally, $\boldsymbol{V} = \text{Cov}(\nabla_{\boldsymbol{\theta}} \ell(y, x, \boldsymbol{g}, v; \boldsymbol{\theta}))$, and $[\cdot]_{\boldsymbol{\beta}\boldsymbol{\beta}}$ represents the block submatrix corresponding to $\boldsymbol{\beta}$.

Proof. According to the conclusion of Corollary 1, the results of Lemma 2 hold by Fan and Li [8]. □

Proof of Theorem 1.

According to the B-spline approximation theory [1, 6, 7], when $\theta \geq 2$ and $L > 0$, there exists a $\boldsymbol{\beta} \in \mathbb{R}^m$ such that

$$\sup_{x\in[a,b]} \left| \sum_{k=1}^{m} \beta_k B_k(x) - f(x) \right| = O\left(m^{-\theta}\right),$$

$$\sup_{x\in[a,b]} \left| \sum_{k=1}^{m} \beta_k B_k'(x) - f'(x) \right| = O\left(m^{-\theta+1}\right).$$

The $O(\cdot)$ holds uniformly for all $f \in \mathcal{H}(\theta, L)$.

Note that for any fixed $x \in [a, b]$,

$$\left[\hat{f}'(x) - f'(x)\right]^2 = \left[\sum_{k=1}^{m} \hat{\beta}_k B_k'(x) - f'(x)\right]^2 = \left[\sum_{k=1}^{m} (\hat{\beta}_k - \beta) B_k'(x) + \sum_{k=1}^{m} \beta_k B_k'(x) - f'(x)\right]^2.$$

Let $\sum_{k=1}^{m} \beta_k B_k'(x) - f'(x) := r_f'(x)$, then

$$\left[\hat{f}'(x) - f'(x)\right]^2 \leq 2\left[\sum_{k=1}^{m} (\hat{\beta}_k - \beta) B_k'(x)\right]^2 + 2\left[r_f'(x)\right]^2$$

and $\sup_f \left[r_f'(x)\right]^2 = O_p(m^{-2\theta+2})$. Denote $\boldsymbol{B}' = \boldsymbol{B}'(x) = (B_1'(x), \ldots, B_m'(x))^T$, and let $f_x(t)$

denote the probability density function of x, then we have

$$\begin{aligned}
\sup_f \int_{[a,b]} \left[\sum_{k=1}^{m}(\hat{\beta}_k - \beta)B'_k(x)\right]^2 &= \sup_f \left(\hat{\boldsymbol{\beta}} - \boldsymbol{\beta}\right)^T \left(\int_{[a,b]} \boldsymbol{B}'(t)\boldsymbol{B}'(t)^T \mathrm{d}t\right) \left(\hat{\boldsymbol{\beta}} - \boldsymbol{\beta}\right) \\
&\leq c^{-1}\sup_f \left(\hat{\boldsymbol{\beta}} - \boldsymbol{\beta}\right)^T \left(\int_{[a,b]} f_x(t)\boldsymbol{B}'(t)\boldsymbol{B}'(t)^T \mathrm{d}t\right) \left(\hat{\boldsymbol{\beta}} - \boldsymbol{\beta}\right) \\
&= c^{-1}\sup_f \left(\hat{\boldsymbol{\beta}} - \boldsymbol{\beta}\right)^T \mathbb{E}\left[\boldsymbol{B}'(x)\boldsymbol{B}'(x)^T\right] \left(\hat{\boldsymbol{\beta}} - \boldsymbol{\beta}\right) \\
&\lesssim m \cdot \sup_f \|\hat{\boldsymbol{\beta}} - \boldsymbol{\beta}\|_2^2 \\
&= o_p(1).
\end{aligned}$$

where the second row applies Assumption 7, the fourth row applies the Proposition C5 from Fan et al.[1]. Furthermore,

$$\sup_f \int_{[a,b]} \left[r'_f(x)\right]^2 \leq \int_{[a,b]} \sup_f \left[r'_f(x)\right]^2 = O_p(m^{-2\theta+2}).$$

Hence, we have

$$\begin{aligned}
\sup_f \int_{[a,b]} \left[\hat{f}'(x) - f'(x)\right]^2 &\leq 2 \cdot \sup_f \int_{[a,b]} \left[\sum_{k=1}^{m}(\hat{\beta}_k - \beta)B'_k(x)\right]^2 + 2 \cdot \sup_f \int_{[a,b]} \left[r'_f(x)\right]^2 \\
&= O_p(m^{-2\theta+2}).
\end{aligned}$$

For (20), it follows that

$$\begin{aligned}
\sqrt{n}\left(\hat{f}'(x) - f'(x)\right) &= \sqrt{n}\left(\sum_{k=1}^{m} \hat{\beta}_k B'_k(x) - f'(x)\right) \\
&= \sqrt{n}\left(\sum_{k=1}^{m} \beta_k B'_k(x) - f'(x)\right) + \sqrt{n}\boldsymbol{B}'(x)^T \left(\hat{\boldsymbol{\beta}} - \boldsymbol{\beta}\right) \\
&= O(\sqrt{n}m^{-\theta+1}) + \sqrt{n}\boldsymbol{B}'(x)^T \left(\hat{\boldsymbol{\beta}} - \boldsymbol{\beta}\right) \\
&= \mathcal{N}\left(0, \boldsymbol{B}'(x)^T \boldsymbol{U}\boldsymbol{B}'(x)\right) + o_p(1).
\end{aligned}$$

□

S2: Additional Demonstrations

1. Extension to nonlinear control function

To complement the baseline framework and provide a more comprehensive formulation, we introduce an extension that accommodates nonlinear control functions. This addition enriches the methodology and allows the framework to cover a broader range of dependence structures. Specifically, instead of restricting the control function to a linear form, we allow for a general nonlinear specification

$$u = q(v) + e, \qquad \text{with} \qquad \mathbb{E}\left[e \mid \boldsymbol{g}, v\right] = \mathbb{E}\left[e \mid v\right] = 0,$$

where $q(\cdot)$ is an unknown smooth function and e denotes the error term. This formulation preserves the same identifying condition as in the baseline framework, while complementing it with additional flexibility in modeling the relationship between u and v.

To approximate $q(\cdot)$ in practice we employ a series representation

$$q(v) \approx \sum_{j=1}^{M} \eta_j \psi_j(v),$$

where $\{\psi_j\}_{j=1}^{M}$ denotes a chosen basis (for example, B-spline or polynomial bases), M is the number of series terms used for q, and $\boldsymbol{\eta} = (\eta_1, \dots, \eta_M)^T$ are unknown coefficients. Let $\boldsymbol{\Psi}(\hat{v})$ denote the design matrix formed by evaluating these basis functions at the estimated residuals. The augmented second-stage estimator then solves

$$\left(\hat{\boldsymbol{\alpha}}, \hat{\boldsymbol{\beta}}, \hat{\boldsymbol{\eta}}\right) = \underset{\boldsymbol{\alpha}, \boldsymbol{\beta}, \rho}{\arg\min} \left\{ \|\boldsymbol{Y} - \boldsymbol{\mathcal{B}}\boldsymbol{\beta} - \boldsymbol{G}\boldsymbol{\alpha} - \boldsymbol{\Psi}(\hat{v})\boldsymbol{\eta}\|_2^2 + p_\lambda^{\text{SCAD}}(\boldsymbol{\alpha}, \boldsymbol{\eta}) \right\},$$

The only modification relative to the baseline procedure is the inclusion of the additional basis $\boldsymbol{\Psi}(\hat{v})$ in the second stage, so the computational and conceptual structure of the algorithm remains almost unchanged.

This extension shows that the nonlinear case can be addressed with a procedure almost identical to the baseline method: the only difference is the inclusion of an additional basis expansion in the second stage. Theoretical properties follow under standard sieve approximation conditions, and alternative estimators such as kernel regression or machine-learning models with cross-fitting may also be adopted if more complex nonlinearities are suspected. In practice, the

computational steps and regularization strategies remain the same as those already employed in the baseline framework. This complementary extension therefore broadens the scope of the method while preserving its overall structure and ease of implementation. A small-scale simulation study evaluating this extension is provided in Section S3.8.

2. Methodology for the binary outcome extension

To extend the MACFIV framework to binary outcomes, we adapt the second stage by integrating the control function method with a logistic regression model. The procedure directly extends the logic of our main framework:

- **Stage 1: Model-averaged control function estimation.**

  This stage remains identical to the procedure for continuous outcomes. The objective is to obtain the individual-level residuals,

  $$\hat{\boldsymbol{v}} = \boldsymbol{X} - \boldsymbol{G}\hat{\boldsymbol{\gamma}}\left(\hat{\boldsymbol{w}}\right)$$

  These residuals serve as the empirical proxy for the unobserved confounder that induces endogeneity.

- **Stage 2: Penalized logistic regression with a control function.**

  In the second stage, the binary outcome $\boldsymbol{Y}$ is modeled using a logistic regression. Crucially, the estimated residual $\hat{\boldsymbol{v}}$ from the first stage is included as an additional regressor to control for the endogeneity of the exposure $\boldsymbol{X}$. The model adopts a logistic link:

  $$\text{logit}\{\mathbb{E}(\boldsymbol{Y} \mid \boldsymbol{X}, \boldsymbol{G})\} = f(\boldsymbol{X}) + \boldsymbol{G}\boldsymbol{\alpha} + \rho\hat{\boldsymbol{v}}$$

  where $f(\boldsymbol{X})$ is approximated by a B-spline basis $f(\boldsymbol{X}) \approx \boldsymbol{\mathcal{B}}\boldsymbol{\beta}$, $\boldsymbol{G}\boldsymbol{\alpha}$ captures potential pleiotropic effects, and $\rho\hat{\boldsymbol{v}}$ represents the control-function adjustment term that accounts for endogeneity in $\boldsymbol{X}$. The estimation of parameters proceeds via penalized maximum likelihood:

  $$\min_{\boldsymbol{\alpha},\boldsymbol{\beta},\rho} \left\{ -\ell(\boldsymbol{Y}; \boldsymbol{X}, \boldsymbol{G}, \hat{\boldsymbol{v}}, \boldsymbol{\alpha}, \boldsymbol{\beta}, \rho) + \sum_{j=1}^{p} p_{\lambda}^{\text{SCAD}}\left(\alpha_j\right) \right\},$$

  where $\ell(\cdot)$ is the logistic log-likelihood function, $\boldsymbol{\beta}$ are the coefficients for the spline basis expansion of $f(\boldsymbol{X})$, and $p_{\lambda}^{\text{SCAD}}(\cdot)$ is the SCAD penalty used to identify and mitigate

pleiotropic instruments. The resulting fitted nonlinear component $\hat{f}(\boldsymbol{X})$ can be used to interpret the shape of the exposure–outcome relationship, while the sign and magnitude of the estimated ρ indicate the extent of endogeneity correction.

This binary-outcome formulation remains consistent with the structure and estimation logic of the primary MACFIV model. It conceptually extends the control function framework to binary settings by replacing the linear specification with a logistic link. The same formulation can also be adapted to categorical or ordinal responses through appropriate link functions, such as the multinomial or cumulative logit link, enabling the MACFIV approach to be applied to a broader range of generalized response models commonly encountered in biomedical data analysis. The implementation details and a simulation study evaluating this extension are provided in Section S3.9.

S3: Additional Simulation and Empirical Results

1. The F-statistic of instruments strength

Table S1 summarizes the distribution of F-statistics for the strength of instruments across simulation scenarios, based on 1000 replications. The F-statistic measures the strength of the association between the instruments and the exposure. The average F-statistic and standard deviation of F-statistics are reported for each scenario. An F-statistic below 10 is typically considered indicative of weak instruments.

Table S1: Distribution of F-statistics for the strength of instruments across simulation scenarios (based on 1000 replications).

Sample Size (n)	Number of Instruments (p)	Average F-statistics	Standard Deviation of F-statistic
200	100	3.98	0.77
500	100	3.97	0.52
1000	100	3.96	0.45
2000	50	3.94	0.56
2000	100	3.98	0.40
2000	150	3.96	0.35
2000	200	3.97	0.30
10000	100	3.94	0.37

2. Simulation results using categorical instrumental variables

Tables S2, S3, S4, and S5 provide supplementary simulation results using categorical instrumental variables, corresponding to the MAE results under Scenarios 1, 2, 3, and 4, respectively.

Table S2: Mean and standard deviation of MAE results for estimating the marginal effect function f' in Scenario 1: fix $n = 2000$, $p = 100$, change the number of pleiotropy instruments $s = 0, 10, 20, 40$.

		TSP		TSP-SCAD		DeepIV		PolyMR		CF		MACFIV	
	f	Mean	SD	Mean	SD	Mean	SD	Mean	SD	Mean	SD	Mean	SD
$s=0$	Null	0.534	0.073	0.534	0.073	0.536	0.049	0.527	0.070	0.417	0.062	0.304	0.084
	Linear	0.613	0.085	0.613	0.085	0.274	0.066	0.526	0.076	0.318	0.069	0.202	0.059
	Quad	0.531	0.073	0.531	0.073	0.538	0.049	0.525	0.076	0.421	0.060	0.314	0.081
	Trig	0.537	0.072	0.537	0.072	0.496	0.053	0.526	0.073	0.405	0.062	0.281	0.085
	Exp	0.525	0.074	0.525	0.074	0.495	0.053	0.525	0.076	0.432	0.062	0.337	0.080
	Log	0.532	0.073	0.531	0.073	0.464	0.056	0.527	0.094	0.419	0.062	0.307	0.082
	Mixed	0.523	0.069	0.523	0.069	0.497	0.053	0.527	0.077	0.473	0.056	0.407	0.072
$s=10$	Null	2.990	0.881	0.537	0.079	0.709	0.091	2.187	0.519	1.760	0.427	0.289	0.085
	Linear	3.042	0.887	0.615	0.088	0.378	0.101	2.186	0.499	1.630	0.433	0.204	0.067
	Quad	2.955	0.915	0.533	0.079	0.709	0.092	2.147	0.512	1.733	0.430	0.306	0.081
	Trig	3.009	0.906	0.542	0.079	0.668	0.095	2.161	0.517	1.719	0.428	0.267	0.088
	Exp	2.975	0.903	0.532	0.082	0.668	0.094	2.177	0.523	1.755	0.411	0.329	0.082
	Log	2.978	0.864	0.540	0.074	0.639	0.099	2.160	0.544	1.723	0.435	0.298	0.082
	Mixed	2.972	0.869	0.527	0.074	0.668	0.094	2.159	0.501	1.786	0.415	0.407	0.073
$s=20$	Null	4.643	1.137	0.542	0.085	0.882	0.117	3.789	0.689	3.076	0.577	0.273	0.089
	Linear	4.678	1.120	0.619	0.094	0.514	0.130	3.777	0.720	2.927	0.592	0.215	0.079
	Quad	4.651	1.118	0.541	0.087	0.882	0.117	3.760	0.682	3.043	0.564	0.291	0.087
	Trig	4.694	1.117	0.550	0.085	0.842	0.120	3.773	0.717	3.046	0.587	0.251	0.090
	Exp	4.642	1.111	0.539	0.092	0.841	0.119	3.780	0.658	3.079	0.569	0.321	0.082
	Log	4.657	1.140	0.542	0.086	0.808	0.121	3.811	0.876	3.069	0.592	0.279	0.090
	Mixed	4.617	1.102	0.537	0.085	0.841	0.119	3.801	0.694	3.128	0.553	0.405	0.078
$s=40$	Null	7.769	1.225	0.566	0.104	1.231	0.164	7.012	0.961	5.759	0.807	0.250	0.099
	Linear	7.741	1.247	0.638	0.115	0.835	0.187	6.950	0.918	5.555	0.809	0.272	0.134
	Quad	7.716	1.233	0.568	0.112	1.231	0.165	6.969	0.952	5.711	0.813	0.274	0.097
	Trig	7.735	1.274	0.568	0.104	1.190	0.167	6.973	1.366	5.667	0.798	0.217	0.096
	Exp	7.717	1.246	0.560	0.107	1.189	0.166	7.003	0.991	5.745	0.820	0.301	0.097
	Log	7.728	1.279	0.563	0.100	1.157	0.168	7.014	1.097	5.718	0.774	0.259	0.098
	Mixed	7.680	1.240	0.548	0.100	1.189	0.167	6.964	0.928	5.760	0.798	0.396	0.085

Table S3: Mean and standard deviation of MAE results for estimating the marginal effect function f' in Scenario 2: fix $p = 100$, $s = 20$, change the sample size $n = 200, 500, 1000, 2000, 10000$.

		TSP		TSP-SCAD		DeepIV		PolyMR		CF		MACFIV	
	f	Mean	SD	Mean	SD	Mean	SD	Mean	SD	Mean	SD	Mean	SD
$n = 200$	Null	1.788	0.362	0.612	0.105	1.152	0.183	3.054	2.911	1.563	0.283	0.343	0.115
	Linear	1.946	0.383	0.813	0.166	1.015	0.200	3.318	6.786	1.654	0.294	0.408	0.137
	Quad	1.770	0.368	0.610	0.110	1.152	0.183	3.160	3.491	1.551	0.289	0.347	0.116
	Trig	1.785	0.345	0.630	0.114	1.137	0.184	3.502	7.428	1.553	0.273	0.340	0.119
	Exp	1.765	0.354	0.590	0.107	1.136	0.183	3.122	3.092	1.537	0.275	0.353	0.115
	Log	1.792	0.358	0.612	0.113	1.120	0.186	3.060	3.727	1.558	0.279	0.340	0.121
	Mixed	1.727	0.349	0.572	0.093	1.137	0.184	3.093	3.723	1.503	0.274	0.403	0.109
$n = 500$	Null	2.541	0.537	0.569	0.089	1.236	0.148	2.703	2.674	1.963	0.350	0.324	0.099
	Linear	2.634	0.535	0.694	0.116	1.105	0.163	2.669	1.326	1.978	0.350	0.293	0.081
	Quad	2.536	0.554	0.563	0.086	1.236	0.148	2.808	4.084	1.956	0.343	0.337	0.099
	Trig	2.592	0.566	0.578	0.092	1.223	0.150	2.721	2.513	1.962	0.353	0.307	0.099
	Exp	2.535	0.580	0.551	0.086	1.222	0.149	2.608	1.279	1.942	0.358	0.345	0.099
	Log	2.569	0.575	0.570	0.095	1.208	0.151	2.635	1.384	1.960	0.364	0.328	0.101
	Mixed	2.508	0.556	0.543	0.089	1.221	0.150	2.653	1.866	1.973	0.357	0.405	0.088
$n = 1000$	Null	3.406	0.768	0.553	0.089	1.211	0.121	2.980	0.686	2.452	0.448	0.307	0.095
	Linear	3.435	0.748	0.646	0.100	1.079	0.134	2.971	0.666	2.361	0.458	0.238	0.066
	Quad	3.413	0.747	0.546	0.088	1.212	0.121	2.979	0.688	2.446	0.440	0.320	0.096
	Trig	3.410	0.766	0.560	0.090	1.197	0.121	2.982	0.790	2.426	0.446	0.288	0.096
	Exp	3.413	0.789	0.544	0.086	1.197	0.122	2.963	0.682	2.436	0.445	0.337	0.092
	Log	3.411	0.732	0.549	0.087	1.185	0.123	3.001	0.870	2.449	0.443	0.307	0.093
	Mixed	3.376	0.780	0.539	0.087	1.197	0.122	2.953	0.667	2.448	0.436	0.410	0.087
$n = 2000$	Null	4.640	1.089	0.542	0.084	0.882	0.117	3.764	0.712	3.058	0.595	0.281	0.091
	Linear	4.698	1.124	0.617	0.091	0.514	0.130	3.733	0.717	2.893	0.597	0.217	0.089
	Quad	4.669	1.089	0.544	0.088	0.882	0.116	3.799	0.709	3.078	0.582	0.294	0.088
	Trig	4.669	1.100	0.550	0.082	0.842	0.120	3.814	0.725	3.072	0.602	0.254	0.088
	Exp	4.658	1.102	0.545	0.088	0.841	0.119	3.773	0.700	3.081	0.583	0.326	0.083
	Log	4.729	1.160	0.544	0.086	0.808	0.121	3.751	0.696	3.059	0.580	0.290	0.092
	Mixed	4.655	1.103	0.533	0.082	0.841	0.119	3.788	0.761	3.116	0.600	0.403	0.078
$n = 10000$	Null	10.009	2.450	0.521	0.076	0.606	0.067	7.694	1.519	5.062	1.153	0.210	0.081
	Linear	9.907	2.431	0.561	0.090	0.209	0.062	7.683	1.582	4.731	1.222	0.362	0.165
	Quad	9.706	2.384	0.522	0.088	0.606	0.068	7.591	1.529	5.023	1.127	0.243	0.085
	Trig	9.941	2.607	0.529	0.155	0.553	0.069	7.643	1.550	5.013	1.181	0.164	0.081
	Exp	9.873	2.455	0.520	0.077	0.552	0.069	7.532	1.435	4.984	1.085	0.268	0.070
	Log	9.884	2.482	0.518	0.089	0.518	0.072	7.599	1.527	5.003	1.156	0.224	0.080
	Mixed	9.952	2.512	0.513	0.088	0.551	0.070	7.701	1.521	5.158	1.143	0.375	0.057

Table S4: Mean and standard deviation of MAE results for estimating the marginal effect function f' in Scenario 3: fix $n = 2000, s = 20$, change the number of instruments $p = 50, 100, 150, 200$.

		TSP		TSP-SCAD		DeepIV		PolyMR		CF		MACFIV	
	f	Mean	SD	Mean	SD	Mean	SD	Mean	SD	Mean	SD	Mean	SD
$p = 50$	Null	8.909	2.114	0.648	0.221	0.749	0.126	7.053	1.321	4.869	0.976	0.188	0.101
	Linear	8.939	2.164	0.704	0.173	0.297	0.110	7.037	1.388	4.583	1.041	0.492	0.211
	Quad	8.922	2.235	0.630	0.218	0.748	0.126	7.063	1.330	4.866	0.981	0.211	0.105
	Trig	8.858	2.212	0.648	0.232	0.690	0.128	7.011	1.348	4.827	0.945	0.157	0.095
	Exp	8.993	2.351	0.638	0.306	0.689	0.128	6.975	1.299	4.893	1.009	0.261	0.106
	Log	8.831	2.297	0.630	0.168	0.642	0.130	6.924	1.318	4.788	0.977	0.196	0.102
	Mixed	8.990	2.167	0.624	0.174	0.689	0.128	6.988	1.348	4.923	0.962	0.395	0.096
$p = 100$	Null	4.679	1.153	0.544	0.082	0.882	0.117	3.773	0.715	3.053	0.590	0.279	0.089
	Linear	4.751	1.155	0.619	0.095	0.514	0.130	3.803	0.721	2.951	0.606	0.216	0.087
	Quad	4.655	1.080	0.538	0.086	0.882	0.117	3.801	0.742	3.078	0.611	0.290	0.089
	Trig	4.675	1.099	0.552	0.085	0.842	0.120	3.802	0.692	3.082	0.591	0.256	0.092
	Exp	4.616	1.095	0.538	0.086	0.841	0.119	3.789	0.703	3.083	0.592	0.318	0.086
	Log	4.584	1.077	0.544	0.085	0.808	0.121	3.763	0.700	3.048	0.582	0.286	0.089
	Mixed	4.599	1.036	0.533	0.086	0.841	0.119	3.778	0.680	3.114	0.566	0.404	0.080
$p = 150$	Null	3.209	0.735	0.525	0.059	0.954	0.117	2.709	0.476	2.323	0.417	0.316	0.073
	Linear	3.246	0.709	0.600	0.073	0.678	0.137	2.737	0.649	2.252	0.432	0.210	0.050
	Quad	3.209	0.714	0.524	0.059	0.953	0.117	2.720	0.488	2.332	0.416	0.330	0.076
	Trig	3.186	0.724	0.528	0.062	0.924	0.119	2.685	0.494	2.293	0.432	0.296	0.079
	Exp	3.228	0.745	0.519	0.059	0.923	0.118	2.677	0.490	2.305	0.434	0.343	0.073
	Log	3.201	0.723	0.524	0.063	0.901	0.122	2.708	0.485	2.321	0.430	0.321	0.075
	Mixed	3.217	0.717	0.515	0.061	0.924	0.120	2.689	0.482	2.335	0.423	0.402	0.068
$p = 200$	Null	2.499	0.535	0.519	0.051	0.981	0.104	2.179	0.365	1.912	0.311	0.337	0.067
	Linear	2.552	0.534	0.590	0.062	0.765	0.124	2.172	0.398	1.867	0.349	0.237	0.058
	Quad	2.488	0.547	0.517	0.049	0.981	0.105	2.164	0.376	1.906	0.324	0.345	0.067
	Trig	2.475	0.544	0.527	0.052	0.959	0.106	2.154	0.388	1.890	0.332	0.323	0.069
	Exp	2.478	0.521	0.514	0.048	0.958	0.106	2.177	0.370	1.921	0.333	0.358	0.062
	Log	2.512	0.549	0.520	0.050	0.941	0.109	2.184	0.516	1.903	0.329	0.338	0.068
	Mixed	2.469	0.523	0.513	0.048	0.958	0.106	2.172	0.384	1.926	0.340	0.406	0.059

Table S5: Mean and standard deviation of MAE results for estimating the marginal effect function f' in Scenario 4: fix $n = 2000, p = 100, s = 20$, start with $\boldsymbol{\gamma} = \left(\sqrt{\frac{2}{n}}, \ldots, \sqrt{\frac{2}{n}}\right)$, then set the first $[\pi p]$ elements to follow a standard multivariate normal distribution to represent strong instruments, varying $\pi = 0.2, 0.4, 0.6, 0.8$.

		TSP		TSP-SCAD		DeepIV		PolyMR		CF		MACFIV	
	f	Mean	SD	Mean	SD	Mean	SD	Mean	SD	Mean	SD	Mean	SD
$\pi = 0.2$	Null	1.038	0.528	0.120	0.116	0.216	0.111	0.134	0.045	0.068	0.029	0.014	0.006
	Linear	1.087	0.552	0.229	0.079	0.228	0.103	0.135	0.045	0.190	0.046	0.177	0.097
	Quad	1.044	0.542	0.131	0.120	0.218	0.111	0.135	0.046	0.074	0.028	0.030	0.007
	Trig	1.075	0.571	0.137	0.142	0.217	0.110	0.135	0.047	0.074	0.030	0.019	0.007
	Exp	1.048	0.571	0.121	0.106	0.218	0.110	0.135	0.048	0.069	0.030	0.015	0.007
	Log	1.021	0.559	0.126	0.139	0.217	0.110	0.133	0.046	0.068	0.028	0.017	0.006
	Mixed	1.040	0.541	0.125	0.106	0.219	0.110	0.134	0.047	0.074	0.026	0.033	0.008
$\pi = 0.4$	Null	0.742	0.371	0.095	0.091	0.111	0.062	0.091	0.032	0.044	0.019	0.010	0.004
	Linear	0.796	0.365	0.201	0.061	0.132	0.056	0.094	0.032	0.171	0.040	0.172	0.102
	Quad	0.764	0.377	0.105	0.088	0.117	0.059	0.093	0.031	0.057	0.018	0.036	0.008
	Trig	0.741	0.373	0.097	0.098	0.113	0.061	0.093	0.031	0.047	0.018	0.013	0.005
	Exp	0.744	0.380	0.093	0.098	0.118	0.059	0.091	0.031	0.045	0.018	0.011	0.004
	Log	0.770	0.393	0.094	0.095	0.113	0.062	0.093	0.032	0.046	0.019	0.013	0.004
	Mixed	0.750	0.377	0.103	0.077	0.123	0.058	0.094	0.032	0.055	0.018	0.033	0.007
$\pi = 0.6$	Null	0.629	0.302	0.081	0.088	0.077	0.041	0.075	0.025	0.036	0.015	0.009	0.003
	Linear	0.682	0.312	0.195	0.057	0.104	0.036	0.076	0.027	0.172	0.040	0.172	0.088
	Quad	0.618	0.305	0.097	0.073	0.087	0.037	0.073	0.024	0.055	0.015	0.043	0.008
	Trig	0.618	0.318	0.078	0.072	0.080	0.039	0.075	0.027	0.036	0.015	0.009	0.004
	Exp	0.641	0.303	0.081	0.085	0.090	0.038	0.074	0.025	0.037	0.015	0.010	0.004
	Log	0.618	0.317	0.082	0.086	0.078	0.041	0.075	0.025	0.036	0.015	0.011	0.003
	Mixed	0.620	0.305	0.089	0.061	0.098	0.037	0.074	0.024	0.053	0.016	0.038	0.008
$\pi = 0.8$	Null	0.534	0.256	0.068	0.065	0.060	0.032	0.064	0.021	0.030	0.012	0.007	0.003
	Linear	0.593	0.261	0.188	0.052	0.092	0.026	0.066	0.021	0.166	0.038	0.175	0.118
	Quad	0.531	0.264	0.095	0.068	0.074	0.027	0.064	0.022	0.057	0.015	0.049	0.009
	Trig	0.534	0.254	0.067	0.068	0.066	0.029	0.065	0.022	0.030	0.012	0.008	0.003
	Exp	0.534	0.270	0.067	0.059	0.079	0.029	0.063	0.020	0.032	0.012	0.012	0.006
	Log	0.551	0.288	0.072	0.069	0.062	0.031	0.064	0.021	0.032	0.013	0.010	0.003
	Mixed	0.541	0.263	0.091	0.073	0.088	0.028	0.064	0.022	0.055	0.015	0.045	0.009

3. Simulation results using continuous instrumental variables

To further examine the performance of our method under a continuous instrumental variable setting, we also conducted simulations with continuous instruments. Specifically, while keeping other settings unchanged, we generate continuous instrumental variables: $\boldsymbol{g}_i^T = (g_{i1}, \dots g_{ip})^T$ is generated from $\mathcal{N}(\mathbf{0}_p, \boldsymbol{\Sigma})$ and $\boldsymbol{\Sigma} = (\rho_{j_1,j_2})_{p\times p}$ with $\rho_{j_1,j_2} = 0.5^{|j_1-j_2|}$ for $j_1, j_2 = 1, \dots, p$, and for each $i = 1, \dots, n$. Figures S1–S4 and Tables S6–S13 provide the supplementary simulation results for all scenarios using continuous instrumental variables. Specifically, Figures S1–S4 present the boxplots of the mean bias, Tables S6–S9 report the RMSE results for Scenarios 1–4, and Tables S10– S13 present the corresponding MAE results.

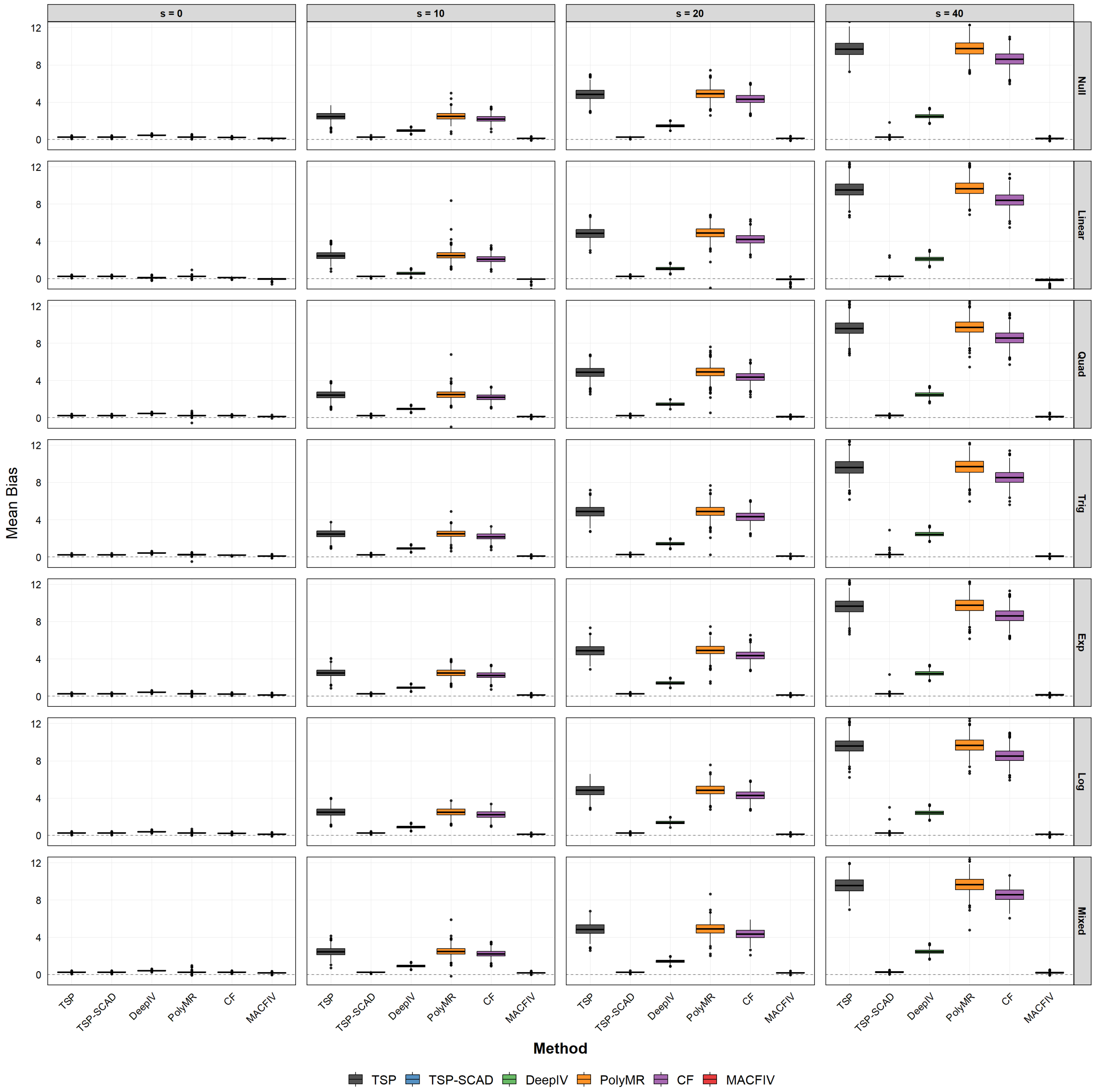

Figure S1: Boxplots of the estimation bias for the marginal effect function f' with continuous instruments in Scenario 1: fix $n = 2000$, $p = 100$, change the number of pleiotropy instruments $s = 0, 10, 20, 40$.

Alt text: Boxplots showing estimation bias of the marginal effect function across multiple methods when using continuous instruments, comparing settings with different numbers of pleiotropic instruments.

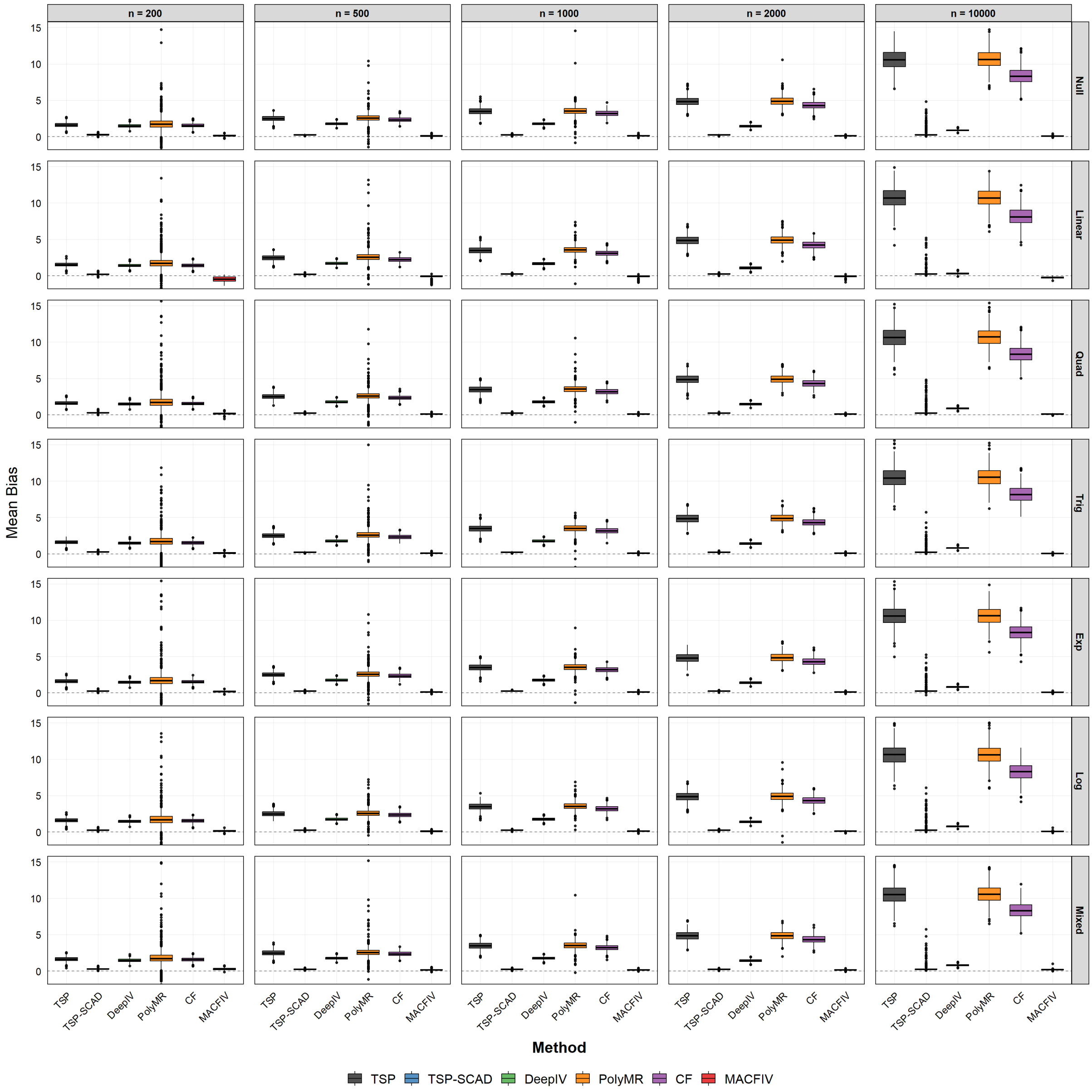

Figure S2: Boxplots of the estimation bias for the marginal effect function f' with continuous instruments in Scenario 2: fix $p = 100$, $s = 20$, change the sample size $n = 200, 500, 1000, 2000, 10000$.

Alt text: Boxplots showing estimation bias of the marginal effect function across multiple methods when using continuous instruments, comparing settings with different sample sizes.

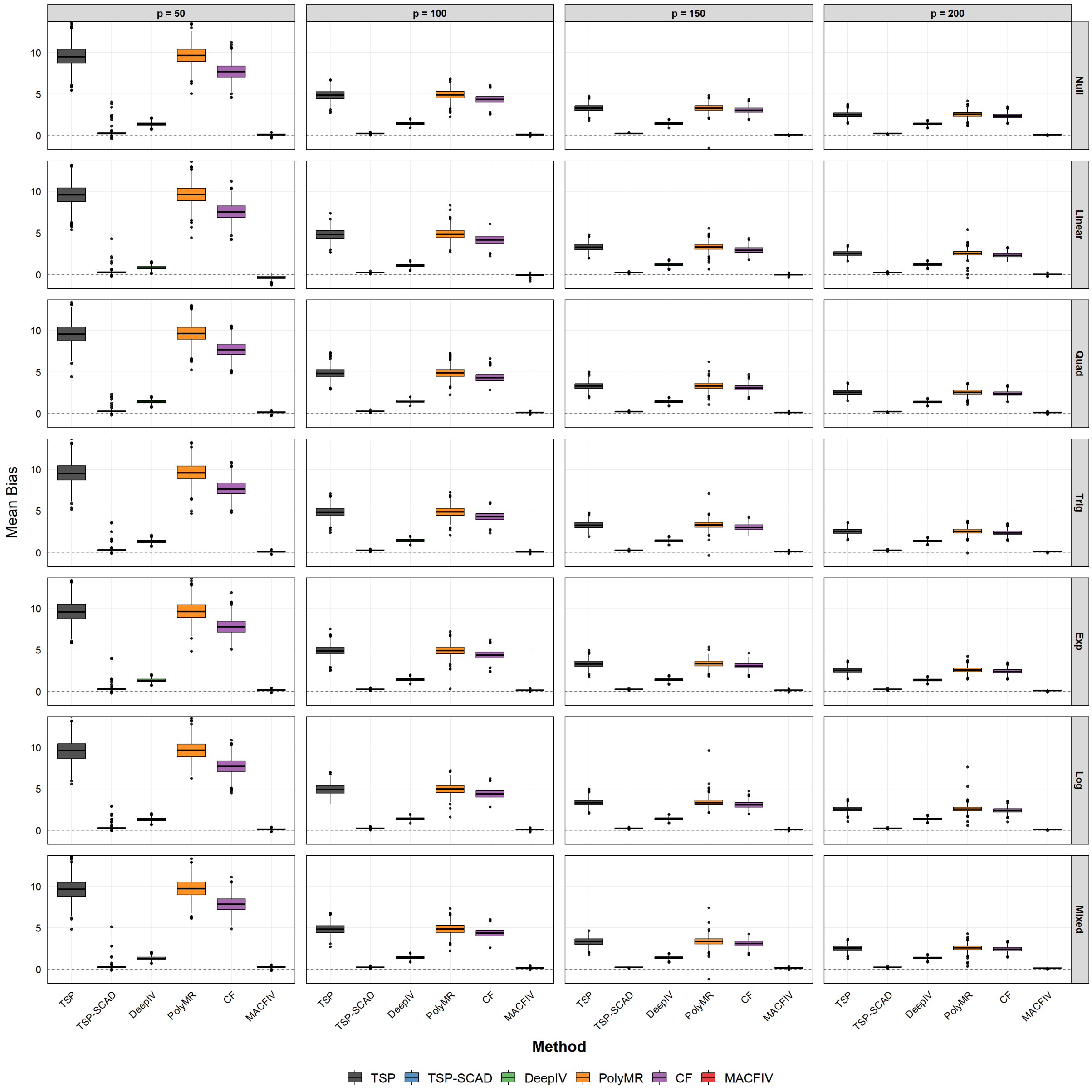

Figure S3: Boxplots of the estimation bias for the marginal effect function f' with continuous instruments in Scenario 3: fix $n = 2000, s = 20$, change the number of instruments $p = 50, 100, 150, 200$.

Alt text: Boxplots showing estimation bias of the marginal effect function across multiple methods when using continuous instruments, comparing settings with different numbers of instruments.

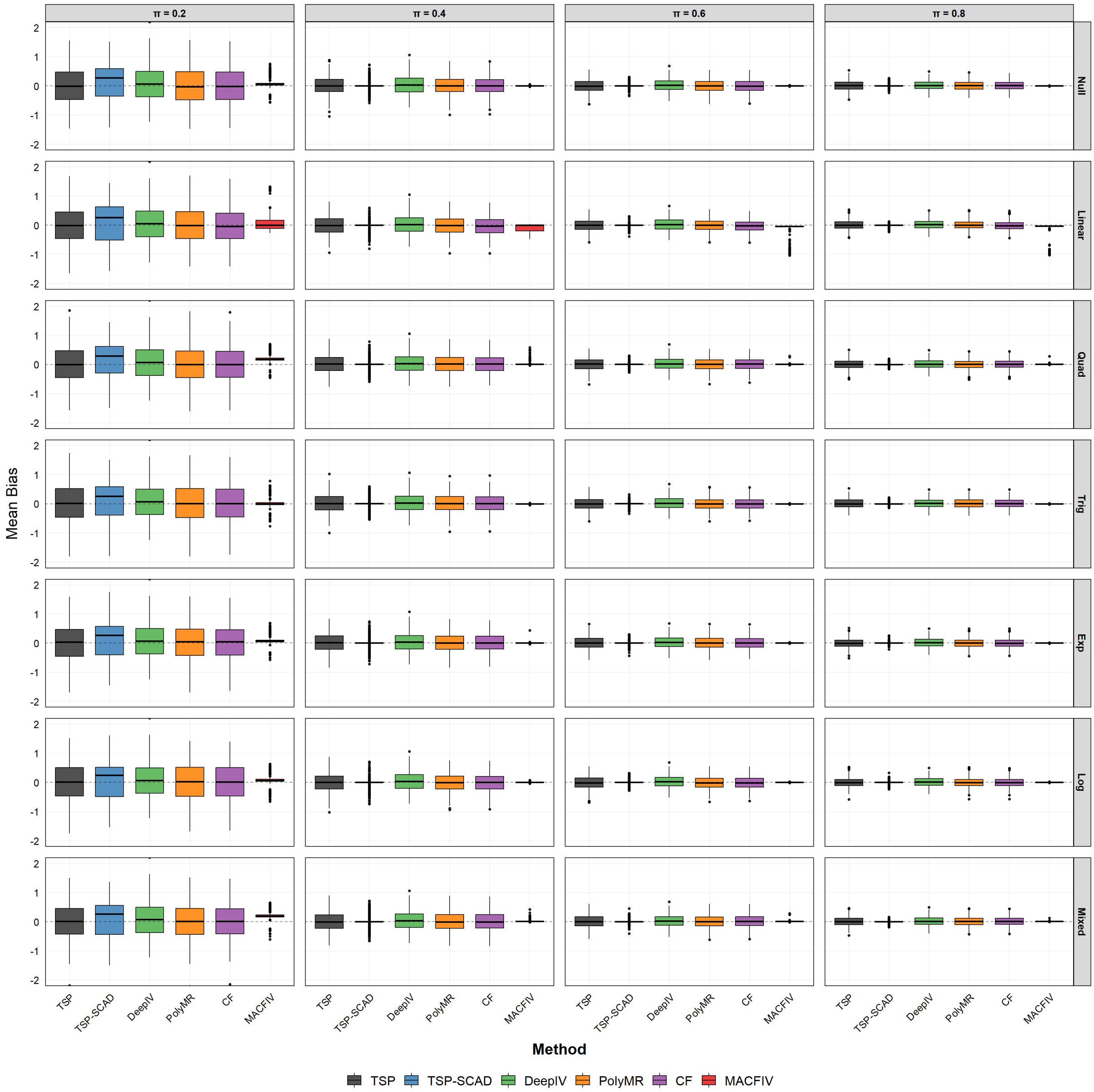

Figure S4: Boxplots of the estimation bias for the marginal effect function f' with continuous instruments in Scenario 4: fix $n = 2000, p = 100, s = 20$, start with $\gamma = \left(\sqrt{\frac{2}{n}}, \ldots, \sqrt{\frac{2}{n}}\right)$, then set the first $[\pi p]$ elements to follow a standard multivariate normal distribution to represent strong instruments, varying $\pi = 0.2, 0.4, 0.6, 0.8$.

Alt text: Boxplots showing estimation bias of the marginal effect function across multiple methods when using continuous instruments, comparing settings with different proportions of strong instruments.

Table S6: Mean and standard deviation of RMSE results for estimating the marginal effect function f' with continuous instruments in Scenario 1: fix $n = 2000$, $p = 100$, change the number of pleiotropy instruments $s = 0, 10, 20, 40$.

		TSP		TSP-SCAD		DeepIV		PolyMR		CF		MACFIV	
	f	Mean	SD	Mean	SD	Mean	SD	Mean	SD	Mean	SD	Mean	SD
$s = 0$	Null	0.430	0.124	0.429	0.124	0.482	0.043	0.680	1.292	0.238	0.047	0.138	0.052
	Linear	0.593	0.136	0.593	0.136	0.277	0.066	0.659	1.064	0.376	0.068	0.321	0.067
	Quad	0.435	0.128	0.434	0.128	0.482	0.043	0.772	3.570	0.243	0.047	0.144	0.054
	Trig	0.446	0.134	0.445	0.134	0.450	0.047	0.666	1.331	0.239	0.048	0.138	0.052
	Exp	0.431	0.130	0.431	0.130	0.449	0.047	0.725	1.647	0.246	0.047	0.152	0.050
	Log	0.434	0.124	0.433	0.123	0.427	0.050	0.79	4.094	0.240	0.050	0.140	0.055
	Mixed	0.431	0.129	0.431	0.129	0.450	0.047	0.748	1.810	0.268	0.045	0.193	0.049
$s = 10$	Null	5.467	1.806	0.449	0.133	0.966	0.128	4.145	5.700	2.315	0.391	0.140	0.058
	Linear	5.413	1.787	0.608	0.138	0.647	0.144	4.023	4.209	2.323	0.396	0.323	0.072
	Quad	5.433	1.835	0.446	0.134	0.966	0.128	4.121	5.308	2.327	0.400	0.143	0.055
	Trig	5.451	1.805	0.459	0.141	0.932	0.130	4.290	6.394	2.346	0.394	0.136	0.053
	Exp	5.353	1.816	0.445	0.135	0.931	0.130	4.039	4.207	2.327	0.396	0.149	0.055
	Log	5.382	1.813	0.462	0.138	0.906	0.131	4.471	9.356	2.330	0.377	0.141	0.055
	Mixed	5.344	1.717	0.444	0.138	0.931	0.130	4.091	4.392	2.364	0.384	0.196	0.052
$s = 20$	Null	8.587	2.374	0.472	0.150	1.486	0.184	6.837	5.990	4.600	0.567	0.137	0.060
	Linear	8.547	2.284	0.619	0.151	1.140	0.200	7.007	8.149	4.585	0.556	0.320	0.075
	Quad	8.391	2.270	0.472	0.144	1.485	0.183	7.546	15.982	3.112	0.571	0.142	0.059
	Trig	8.480	2.365	0.482	0.146	1.450	0.184	6.957	9.213	4.579	0.553	0.134	0.055
	Exp	8.479	2.274	0.471	0.141	1.450	0.185	7.290	10.222	4.582	0.579	0.153	0.057
	Log	8.428	2.303	0.467	0.142	1.424	0.186	6.689	6.945	4.543	0.550	0.137	0.057
	Mixed	8.344	2.228	0.466	0.145	1.449	0.185	7.094	7.930	4.577	0.552	0.201	0.055
$s = 40$	Null	13.614	2.751	0.520	0.266	2.526	0.253	11.538	8.200	9.049	0.761	0.134	0.063
	Linear	13.668	2.755	0.666	0.173	2.167	0.269	12.227	13.231	9.006	0.762	0.344	0.110
	Quad	13.535	2.602	0.520	0.331	2.526	0.254	12.755	20.454	9.056	0.762	0.142	0.064
	Trig	13.585	2.740	0.535	0.294	2.491	0.253	11.346	5.975	9.001	0.777	0.129	0.058
	Exp	13.382	2.472	0.509	0.172	2.490	0.254	11.834	8.901	8.985	0.771	0.157	0.063
	Log	13.528	2.715	0.531	0.270	2.463	0.256	12.010	11.176	9.073	0.733	0.138	0.063
	Mixed	13.437	2.633	0.541	0.465	2.489	0.255	12.663	18.629	9.066	0.767	0.209	0.062

Table S7: Mean and standard deviation of RMSE results for estimating the marginal effect function f' with continuous instruments in Scenario 2: fix $p = 100$, $s = 20$, change the sample size $n = 200, 500, 1000, 2000, 10000$.

		TSP		TSP-SCAD		DeepIV		PolyMR		CF		MACFIV	
	f	Mean	SD	Mean	SD	Mean	SD	Mean	SD	Mean	SD	Mean	SD
$n = 200$	Null	2.599	0.615	0.481	0.142	1.579	0.235	14.142	34.194	2.073	0.370	0.270	0.113
	Linear	2.890	0.690	0.974	0.264	1.518	0.244	14.305	35.148	2.368	0.460	0.719	0.187
	Quad	2.619	0.644	0.468	0.142	1.579	0.234	12.669	23.563	2.065	0.371	0.275	0.114
	Trig	2.612	0.608	0.505	0.139	1.572	0.235	15.797	42.790	2.080	0.358	0.272	0.115
	Exp	2.599	0.635	0.465	0.150	1.571	0.235	15.488	53.972	2.057	0.371	0.294	0.116
	Log	2.622	0.641	0.481	0.140	1.568	0.236	18.091	70.732	2.081	0.371	0.270	0.113
	Mixed	2.545	0.621	0.462	0.128	1.573	0.235	16.662	47.027	2.004	0.360	0.345	0.116
$n = 500$	Null	4.175	1.096	0.460	0.138	1.844	0.208	7.777	10.058	2.689	0.371	0.172	0.068
	Linear	4.306	1.039	0.759	0.185	1.773	0.214	9.325	29.308	2.834	0.390	0.489	0.130
	Quad	4.200	1.087	0.459	0.137	1.844	0.209	9.888	27.756	2.701	0.375	0.172	0.066
	Trig	4.205	1.080	0.471	0.135	1.837	0.208	8.876	15.628	2.714	0.361	0.180	0.067
	Exp	4.173	1.056	0.446	0.139	1.837	0.209	9.734	29.849	2.671	0.360	0.177	0.065
	Log	4.212	1.065	0.459	0.136	1.832	0.210	10.639	25.385	2.690	0.357	0.176	0.068
	Mixed	4.113	1.049	0.447	0.132	1.838	0.209	9.490	28.039	2.676	0.352	0.229	0.064
$n = 1000$	Null	5.928	1.599	0.470	0.143	1.835	0.193	7.811	15.817	3.456	0.429	0.154	0.059
	Linear	6.051	1.554	0.666	0.154	1.734	0.203	7.958	17.486	3.512	0.447	0.388	0.092
	Quad	5.973	1.614	0.458	0.139	1.834	0.193	7.130	9.864	3.472	0.425	0.155	0.062
	Trig	5.935	1.545	0.467	0.135	1.825	0.194	6.352	8.584	3.472	0.447	0.154	0.058
	Exp	5.885	1.563	0.465	0.146	1.825	0.193	8.851	47.285	3.454	0.444	0.163	0.058
	Log	6.012	1.639	0.468	0.144	1.817	0.194	7.595	16.177	3.460	0.430	0.154	0.061
	Mixed	5.985	1.662	0.448	0.132	1.826	0.193	6.674	11.420	3.490	0.443	0.210	0.054
$n = 2000$	Null	8.547	2.359	0.470	0.146	1.486	0.184	6.981	8.512	4.563	0.576	0.137	0.058
	Linear	8.478	2.245	0.619	0.143	1.140	0.200	6.854	9.263	4.556	0.570	0.322	0.074
	Quad	8.529	2.475	0.464	0.141	1.485	0.183	6.872	7.892	4.552	0.578	0.143	0.057
	Trig	8.494	2.353	0.474	0.142	1.450	0.184	6.763	5.464	4.572	0.559	0.133	0.055
	Exp	8.464	2.414	0.463	0.146	1.450	0.185	7.206	11.887	4.567	0.574	0.154	0.058
	Log	8.594	2.357	0.469	0.147	1.424	0.186	7.162	11.859	4.574	0.548	0.142	0.059
	Mixed	8.503	2.347	0.462	0.143	1.449	0.185	7.181	12.431	4.564	0.594	0.200	0.053
$n = 10000$	Null	18.777	5.349	0.734	1.418	0.894	0.109	11.066	2.750	8.513	1.149	0.099	0.095
	Linear	18.825	5.229	0.720	1.143	0.410	0.116	10.970	2.972	8.339	1.156	0.314	0.097
	Quad	18.391	5.106	0.643	1.095	0.895	0.109	10.950	2.138	8.468	1.115	0.105	0.050
	Trig	18.727	5.203	0.643	1.247	0.841	0.111	11.140	3.667	8.494	1.127	0.081	0.068
	Exp	18.881	5.256	0.659	1.196	0.842	0.112	10.983	1.627	8.559	1.167	0.124	0.051
	Log	18.391	5.106	0.643	1.095	0.809	0.113	10.950	2.138	8.468	1.115	0.105	0.050
	Mixed	18.818	5.394	0.679	1.173	0.841	0.111	10.944	1.916	8.553	1.191	0.200	0.052

Table S8: Mean and standard deviation of RMSE results for estimating the marginal effect function f' with continuous instruments in Scenario 3: fix $n = 2000, s = 20$, change the number of instruments $p = 50, 100, 150, 200$.

		TSP		TSP-SCAD		DeepIV		PolyMR		CF		MACFIV	
	f	Mean	SD	Mean	SD	Mean	SD	Mean	SD	Mean	SD	Mean	SD
$p = 50$	Null	16.558	4.518	0.786	1.058	1.442	0.200	11.873	9.636	8.192	0.982	0.136	0.071
	Linear	16.588	4.522	0.932	1.205	0.905	0.215	11.929	13.655	8.043	0.966	0.456	0.192
	Quad	16.443	4.484	0.789	1.050	1.442	0.201	12.492	11.487	8.173	0.960	0.151	0.075
	Trig	16.469	4.563	0.818	1.138	1.386	0.201	12.963	14.090	8.222	0.968	0.127	0.064
	Exp	16.444	4.601	0.815	1.079	1.385	0.202	12.279	9.652	8.109	0.956	0.174	0.076
	Log	16.515	4.560	0.800	0.980	1.342	0.202	12.011	10.589	8.120	0.938	0.142	0.072
	Mixed	16.569	4.615	0.771	0.886	1.385	0.201	12.480	15.811	8.193	0.961	0.257	0.080
$p = 100$	Null	8.513	2.443	0.463	0.139	1.486	0.184	6.926	7.066	4.627	0.554	0.132	0.058
	Linear	8.497	2.238	0.627	0.143	1.140	0.200	6.584	4.219	4.576	0.550	0.321	0.072
	Quad	8.410	2.275	0.464	0.148	1.485	0.183	7.051	12.157	4.548	0.551	0.143	0.057
	Trig	8.420	2.263	0.468	0.144	1.450	0.184	7.140	11.329	4.548	0.539	0.130	0.055
	Exp	8.469	2.320	0.465	0.140	1.450	0.185	6.705	8.814	4.567	0.563	0.151	0.056
	Log	8.505	2.331	0.466	0.133	1.424	0.186	7.080	12.579	4.572	0.584	0.142	0.057
	Mixed	8.336	2.246	0.464	0.143	1.449	0.185	6.935	8.792	4.567	0.569	0.201	0.054
$p = 150$	Null	5.620	1.530	0.399	0.102	1.468	0.166	5.015	6.468	3.204	0.404	0.136	0.050
	Linear	5.738	1.499	0.555	0.109	1.238	0.181	4.876	4.331	3.238	0.405	0.324	0.068
	Quad	5.689	1.529	0.391	0.102	1.468	0.165	5.551	10.211	3.216	0.405	0.140	0.050
	Trig	5.645	1.550	0.400	0.100	1.444	0.166	5.709	11.929	3.237	0.403	0.133	0.047
	Exp	5.695	1.579	0.390	0.098	1.443	0.167	4.789	4.065	3.224	0.409	0.143	0.046
	Log	5.641	1.514	0.399	0.100	1.427	0.168	5.118	6.154	3.224	0.399	0.136	0.048
	Mixed	5.627	1.617	0.393	0.102	1.443	0.166	5.074	7.412	3.207	0.409	0.181	0.045
$p = 200$	Null	4.256	1.101	0.361	0.079	1.410	0.157	4.454	10.998	2.528	0.337	0.131	0.046
	Linear	4.275	1.096	0.533	0.099	1.250	0.167	4.132	7.913	2.571	0.306	0.328	0.069
	Quad	4.260	1.111	0.352	0.078	1.410	0.156	3.931	3.750	2.531	0.338	0.132	0.044
	Trig	4.275	1.115	0.367	0.077	1.393	0.157	4.309	5.849	2.541	0.318	0.136	0.043
	Exp	4.190	1.123	0.355	0.076	1.394	0.158	4.002	4.502	2.519	0.320	0.139	0.041
	Log	4.280	1.164	0.358	0.079	1.384	0.158	4.262	6.030	2.539	0.320	0.131	0.045
	Mixed	4.151	1.094	0.349	0.077	1.394	0.158	4.065	5.323	2.509	0.315	0.170	0.038

Table S9: Mean and standard deviation of RMSE results for estimating the marginal effect function f' with continuous instruments in Scenario 4: fix $n = 2000, p = 100, s = 20$, start with $\gamma = \left(\sqrt{\frac{2}{n}}, \ldots, \sqrt{\frac{2}{n}}\right)$, then set the first $[\pi p]$ elements to follow a standard multivariate normal distribution to represent strong instruments, varying $\pi = 0.2, 0.4, 0.6, 0.8$.

		TSP		TSP-SCAD		DeepIV		PolyMR		CF		MACFIV	
	f	Mean	SD	Mean	SD	Mean	SD	Mean	SD	Mean	SD	Mean	SD
	Null	2.395	1.169	0.359	0.456	0.500	0.296	0.632	0.411	0.170	0.071	0.019	0.008
	Linear	2.458	1.172	0.496	0.363	0.513	0.286	0.611	0.437	0.384	0.089	0.327	0.117
	Quad	2.397	1.161	0.354	0.444	0.503	0.294	0.609	0.398	0.173	0.066	0.053	0.011
$\pi = 0.2$	Trig	2.429	1.202	0.369	0.468	0.501	0.295	0.636	0.403	0.179	0.075	0.028	0.009
	Exp	2.390	1.139	0.336	0.443	0.503	0.295	0.606	0.450	0.168	0.070	0.020	0.008
	Log	2.369	1.107	0.348	0.446	0.502	0.295	0.617	0.507	0.167	0.067	0.025	0.006
	Mixed	2.450	1.214	0.390	0.532	0.505	0.293	0.629	0.384	0.176	0.067	0.058	0.013
	Null	1.709	0.832	0.263	0.339	0.279	0.156	0.419	0.330	0.105	0.041	0.013	0.006
	Linear	1.747	0.803	0.424	0.217	0.302	0.147	0.444	0.293	0.347	0.077	0.332	0.128
	Quad	1.734	0.799	0.292	0.320	0.286	0.153	0.428	0.249	0.121	0.033	0.070	0.015
$\pi = 0.4$	Trig	1.700	0.789	0.267	0.337	0.280	0.155	0.429	0.270	0.107	0.040	0.019	0.007
	Exp	1.740	0.812	0.251	0.328	0.286	0.152	0.428	0.226	0.107	0.040	0.015	0.005
	Log	1.698	0.826	0.267	0.334	0.281	0.155	0.428	0.252	0.104	0.040	0.022	0.004
	Mixed	1.701	0.847	0.278	0.325	0.294	0.149	0.430	0.254	0.117	0.036	0.063	0.013
	Null	1.435	0.645	0.221	0.280	0.194	0.104	0.358	0.245	0.083	0.031	0.011	0.005
	Linear	1.486	0.670	0.382	0.163	0.225	0.095	0.363	0.280	0.328	0.074	0.324	0.129
	Quad	1.407	0.663	0.262	0.273	0.206	0.100	0.346	0.197	0.113	0.026	0.082	0.016
$\pi = 0.6$	Trig	1.385	0.635	0.233	0.285	0.198	0.103	0.356	0.216	0.082	0.031	0.013	0.005
	Exp	1.386	0.639	0.211	0.266	0.214	0.096	0.357	0.255	0.082	0.031	0.016	0.011
	Log	1.389	0.660	0.233	0.282	0.197	0.103	0.349	0.238	0.083	0.031	0.021	0.003
	Mixed	1.387	0.669	0.253	0.280	0.228	0.092	0.346	0.248	0.107	0.026	0.074	0.015
	Null	1.248	0.570	0.184	0.224	0.152	0.079	0.306	0.195	0.071	0.028	0.010	0.004
	Linear	1.251	0.554	0.375	0.142	0.188	0.069	0.305	0.192	0.327	0.079	0.335	0.149
	Quad	1.221	0.565	0.244	0.232	0.167	0.073	0.307	0.172	0.116	0.023	0.095	0.018
$\pi = 0.8$	Trig	1.244	0.574	0.206	0.267	0.158	0.076	0.314	0.196	0.072	0.029	0.012	0.004
	Exp	1.213	0.571	0.195	0.227	0.191	0.068	0.305	0.187	0.075	0.026	0.023	0.014
	Log	1.161	0.557	0.198	0.242	0.156	0.077	0.311	0.247	0.071	0.026	0.021	0.002
	Mixed	1.227	0.583	0.224	0.218	0.208	0.065	0.308	0.174	0.110	0.023	0.088	0.018

Table S10: Mean and standard deviation of MAE results for estimating the marginal effect function f' with continuous instruments in Scenario 1: fix $n = 2000$, $p = 100$, change the number of pleiotropy instruments $s = 0, 10, 20, 40$.

		TSP		TSP-SCAD		DeepIV		PolyMR		CF		MACFIV	
	f	Mean	SD	Mean	SD	Mean	SD	Mean	SD	Mean	SD	Mean	SD
$s=0$	Null	0.339	0.091	0.339	0.091	0.462	0.042	0.276	0.055	0.228	0.047	0.127	0.053
	Linear	0.431	0.108	0.431	0.107	0.235	0.059	0.275	0.055	0.226	0.042	0.175	0.041
	Quad	0.343	0.093	0.342	0.093	0.462	0.043	0.282	0.093	0.234	0.047	0.133	0.055
	Trig	0.354	0.101	0.353	0.101	0.425	0.046	0.276	0.058	0.224	0.048	0.121	0.052
	Exp	0.341	0.101	0.340	0.101	0.424	0.046	0.280	0.060	0.239	0.047	0.142	0.052
	Log	0.341	0.094	0.341	0.094	0.398	0.049	0.279	0.103	0.230	0.050	0.129	0.055
	Mixed	0.341	0.101	0.340	0.100	0.425	0.046	0.278	0.064	0.260	0.047	0.175	0.056
$s=10$	Null	4.232	1.397	0.354	0.101	0.936	0.128	2.573	0.438	2.255	0.385	0.128	0.059
	Linear	4.230	1.450	0.440	0.104	0.588	0.138	2.577	0.442	2.213	0.394	0.180	0.054
	Quad	4.192	1.411	0.352	0.103	0.935	0.128	2.581	0.454	2.268	0.396	0.133	0.056
	Trig	4.243	1.441	0.361	0.106	0.899	0.130	2.607	0.456	2.282	0.388	0.119	0.053
	Exp	4.171	1.435	0.349	0.103	0.898	0.130	2.583	0.437	2.272	0.390	0.140	0.057
	Log	4.185	1.448	0.361	0.104	0.870	0.131	2.596	0.461	2.270	0.371	0.130	0.056
	Mixed	4.100	1.306	0.347	0.103	0.898	0.130	2.614	0.425	2.316	0.379	0.179	0.059
$s=20$	Null	6.756	1.791	0.368	0.114	1.449	0.183	5.041	0.638	4.493	0.560	0.125	0.061
	Linear	6.768	1.719	0.454	0.119	1.078	0.199	5.048	0.624	4.438	0.553	0.184	0.066
	Quad	6.651	1.663	0.370	0.112	1.448	0.183	5.037	0.750	3.043	0.564	0.131	0.060
	Trig	6.698	1.778	0.375	0.114	1.411	0.184	5.030	0.632	4.470	0.547	0.118	0.056
	Exp	6.716	1.673	0.368	0.111	1.410	0.184	5.045	0.650	4.482	0.575	0.144	0.060
	Log	6.631	1.664	0.364	0.108	1.383	0.186	4.983	0.617	4.440	0.544	0.126	0.058
	Mixed	6.589	1.637	0.363	0.110	1.410	0.185	5.018	0.619	4.485	0.546	0.185	0.062
$s=40$	Null	11.243	1.839	0.400	0.196	2.479	0.253	9.850	0.844	8.855	0.756	0.123	0.063
	Linear	11.322	1.852	0.491	0.140	2.103	0.270	9.837	0.891	8.773	0.759	0.226	0.126
	Quad	11.149	1.691	0.403	0.255	2.478	0.253	9.891	0.940	8.863	0.760	0.132	0.065
	Trig	11.160	1.786	0.417	0.214	2.442	0.254	9.797	0.861	8.802	0.774	0.113	0.059
	Exp	11.114	1.690	0.395	0.135	2.441	0.254	9.789	0.860	8.799	0.766	0.147	0.065
	Log	11.221	1.828	0.407	0.175	2.413	0.257	9.888	0.861	8.879	0.729	0.126	0.064
	Mixed	11.148	1.759	0.418	0.331	2.440	0.255	9.883	0.959	8.887	0.765	0.194	0.069

Table S11: Mean and standard deviation of MAE results for estimating the marginal effect function f' with continuous instruments in Scenario 2: fix $p = 100$, $s = 20$, change the sample size $n = 200, 500, 1000, 2000, 10000$.

		TSP		TSP-SCAD		DeepIV		PolyMR		CF		MACFIV	
	f	Mean	SD	Mean	SD	Mean	SD	Mean	SD	Mean	SD	Mean	SD
$n = 200$	Null	2.109	0.425	0.385	0.105	1.487	0.226	3.413	2.601	1.814	0.300	0.223	0.102
	Linear	2.258	0.465	0.614	0.170	1.409	0.233	3.410	2.691	1.923	0.330	0.538	0.262
	Quad	2.118	0.445	0.376	0.102	1.487	0.225	3.286	1.929	1.810	0.299	0.229	0.104
	Trig	2.124	0.437	0.399	0.100	1.479	0.226	3.515	3.249	1.817	0.292	0.216	0.100
	Exp	2.108	0.439	0.372	0.108	1.479	0.225	3.497	3.997	1.812	0.300	0.249	0.109
	Log	2.126	0.446	0.380	0.101	1.471	0.227	3.704	5.118	1.821	0.305	0.221	0.103
	Mixed	2.061	0.447	0.371	0.091	1.479	0.226	3.577	3.511	1.779	0.306	0.296	0.121
$n = 500$	Null	3.337	0.758	0.365	0.103	1.770	0.207	3.099	0.666	2.532	0.348	0.145	0.064
	Linear	2.634	0.535	0.694	0.116	1.686	0.212	2.669	1.326	1.978	0.350	0.293	0.081
	Quad	3.358	0.798	0.361	0.103	1.770	0.208	3.214	1.368	2.543	0.349	0.147	0.065
	Trig	3.364	0.760	0.369	0.099	1.762	0.207	3.170	0.864	2.550	0.339	0.146	0.061
	Exp	3.311	0.749	0.352	0.104	1.762	0.208	3.192	1.437	2.517	0.341	0.151	0.064
	Log	3.344	0.745	0.360	0.101	1.755	0.208	3.226	1.261	2.527	0.340	0.147	0.065
	Mixed	3.291	0.755	0.351	0.098	1.761	0.208	3.199	1.342	2.541	0.339	0.187	0.071
$n = 1000$	Null	4.715	1.163	0.369	0.108	1.781	0.191	3.806	0.688	3.335	0.418	0.135	0.058
	Linear	4.776	1.132	0.469	0.116	1.666	0.202	3.807	0.747	3.325	0.433	0.210	0.072
	Quad	4.747	1.204	0.361	0.104	1.780	0.191	3.798	0.570	3.354	0.415	0.138	0.062
	Trig	4.711	1.158	0.367	0.102	1.769	0.192	3.760	0.553	3.347	0.437	0.132	0.057
	Exp	4.688	1.172	0.366	0.110	1.768	0.193	3.836	1.569	3.340	0.436	0.146	0.060
	Log	4.756	1.197	0.367	0.109	1.760	0.193	3.799	0.698	3.338	0.420	0.135	0.060
	Mixed	4.754	1.238	0.352	0.100	1.769	0.192	3.806	0.591	3.383	0.430	0.181	0.063
$n = 2000$	Null	6.719	1.719	0.368	0.114	1.449	0.183	5.017	0.651	4.461	0.569	0.126	0.059
	Linear	6.741	1.703	0.452	0.113	1.078	0.199	5.014	0.651	4.410	0.567	0.184	0.061
	Quad	6.725	1.853	0.363	0.107	1.448	0.183	4.997	0.647	4.450	0.570	0.133	0.058
	Trig	6.715	1.788	0.373	0.111	1.411	0.184	5.021	0.612	4.464	0.551	0.117	0.056
	Exp	6.669	1.750	0.363	0.111	1.410	0.184	5.019	0.685	4.467	0.568	0.144	0.061
	Log	6.788	1.752	0.367	0.111	1.383	0.186	5.022	0.643	4.470	0.542	0.131	0.059
	Mixed	6.645	1.730	0.365	0.110	1.410	0.185	5.004	0.699	4.473	0.587	0.183	0.061
$n = 10000$	Null	14.859	4.084	0.542	0.985	0.869	0.108	10.658	1.280	8.411	1.151	0.095	0.095
	Linear	14.768	4.121	0.531	0.836	0.353	0.106	10.644	1.288	8.218	1.159	0.267	0.111
	Quad	14.700	3.991	0.562	0.993	0.869	0.108	10.693	1.270	8.469	1.137	0.108	0.051
	Trig	14.768	3.934	0.488	0.950	0.813	0.110	10.634	1.255	8.390	1.128	0.073	0.069
	Exp	14.957	4.077	0.501	0.929	0.812	0.110	10.694	1.319	8.457	1.166	0.122	0.052
	Log	14.499	3.777	0.472	0.735	0.778	0.112	10.583	1.289	8.366	1.114	0.101	0.051
	Mixed	14.866	4.136	0.496	0.811	0.811	0.110	10.621	1.355	8.456	1.189	0.196	0.055

Table S12: Mean and standard deviation of MAE results for estimating the marginal effect function f' with continuous instruments in Scenario 3: fix $n = 2000, s = 20$, change the number of instruments $p = 50, 100, 150, 200$.

		TSP		TSP-SCAD		DeepIV		PolyMR		CF		MACFIV	
	f	Mean	SD	Mean	SD	Mean	SD	Mean	SD	Mean	SD	Mean	SD
$p = 50$	Null	13.111	3.363	0.596	0.787	1.390	0.201	9.841	1.157	8.020	0.981	0.125	0.071
	Linear	13.123	3.332	0.683	0.898	0.819	0.210	9.758	1.179	7.836	0.976	0.388	0.226
	Quad	13.042	3.303	0.594	0.802	1.390	0.201	9.835	1.149	8.004	0.958	0.140	0.077
	Trig	13.058	3.340	0.618	0.810	1.331	0.202	9.885	1.196	8.049	0.967	0.112	0.065
	Exp	13.075	3.517	0.609	0.749	1.330	0.203	9.748	1.155	7.942	0.954	0.164	0.079
	Log	13.024	3.358	0.589	0.598	1.283	0.204	9.755	1.145	7.948	0.938	0.130	0.073
	Mixed	13.116	3.428	0.583	0.672	1.330	0.202	9.807	1.208	8.034	0.957	0.245	0.087
$p = 100$	Null	6.729	1.804	0.362	0.110	1.449	0.183	5.073	0.619	4.522	0.548	0.120	0.058
	Linear	6.711	1.642	0.459	0.116	1.078	0.199	5.031	0.595	4.428	0.547	0.184	0.058
	Quad	6.654	1.687	0.364	0.114	1.448	0.183	4.998	0.656	4.447	0.545	0.133	0.057
	Trig	6.652	1.700	0.365	0.111	1.411	0.184	4.997	0.640	4.441	0.536	0.114	0.056
	Exp	6.680	1.746	0.362	0.108	1.410	0.184	5.013	0.643	4.467	0.557	0.142	0.059
	Log	6.685	1.729	0.364	0.102	1.383	0.186	5.032	0.691	4.469	0.578	0.130	0.059
	Mixed	6.603	1.709	0.364	0.111	1.410	0.185	5.021	0.650	4.474	0.561	0.184	0.061
$p = 150$	Null	4.428	1.092	0.321	0.077	1.434	0.165	3.426	0.450	3.124	0.399	0.125	0.051
	Linear	4.558	1.115	0.400	0.080	1.186	0.182	3.425	0.443	3.107	0.402	0.175	0.040
	Quad	4.496	1.144	0.313	0.075	1.435	0.165	3.442	0.477	3.137	0.399	0.130	0.051
	Trig	4.477	1.158	0.322	0.074	1.410	0.166	3.471	0.509	3.153	0.399	0.118	0.048
	Exp	4.498	1.200	0.311	0.070	1.408	0.167	3.437	0.437	3.146	0.403	0.133	0.048
	Log	4.465	1.131	0.317	0.072	1.391	0.168	3.444	0.433	3.141	0.392	0.125	0.049
	Mixed	4.466	1.204	0.314	0.073	1.409	0.166	3.428	0.461	3.138	0.404	0.163	0.051
$p = 200$	Null	3.383	0.811	0.296	0.056	1.378	0.156	2.676	0.427	2.457	0.332	0.120	0.046
	Linear	3.391	0.797	0.384	0.067	1.203	0.167	2.664	0.362	2.453	0.302	0.177	0.037
	Quad	3.374	0.844	0.290	0.056	1.379	0.155	2.665	0.356	2.462	0.332	0.122	0.045
	Trig	3.405	0.833	0.303	0.056	1.360	0.157	2.683	0.357	2.465	0.313	0.120	0.044
	Exp	3.321	0.798	0.291	0.054	1.361	0.157	2.659	0.351	2.453	0.314	0.130	0.042
	Log	3.394	0.844	0.294	0.056	1.349	0.158	2.681	0.359	2.467	0.317	0.120	0.045
	Mixed	3.303	0.804	0.286	0.053	1.361	0.158	2.653	0.347	2.449	0.311	0.150	0.043

Table S13: Mean and standard deviation of MAE results for estimating the marginal effect function f' with continuous instruments in Scenario 4: fix $n = 2000, p = 100, s = 20$, start with $\gamma = \left(\sqrt{\frac{2}{n}}, \ldots, \sqrt{\frac{2}{n}}\right)$, then set the first $[\pi p]$ elements to follow a standard multivariate normal distribution to represent strong instruments, varying $\pi = 0.2, 0.4, 0.6, 0.8$.

		TSP		TSP-SCAD		DeepIV		PolyMR		CF		MACFIV	
	f	Mean	SD	Mean	SD	Mean	SD	Mean	SD	Mean	SD	Mean	SD
$\pi = 0.2$	Null	1.816	0.940	0.272	0.356	0.481	0.301	0.234	0.084	0.139	0.060	0.015	0.007
	Linear	1.870	0.938	0.316	0.287	0.487	0.293	0.229	0.080	0.232	0.064	0.179	0.122
	Quad	1.819	0.932	0.265	0.342	0.482	0.300	0.233	0.083	0.140	0.058	0.030	0.008
	Trig	1.847	0.958	0.283	0.370	0.481	0.301	0.239	0.085	0.146	0.064	0.019	0.007
	Exp	1.821	0.935	0.252	0.336	0.482	0.300	0.230	0.085	0.138	0.060	0.015	0.007
	Log	1.798	0.903	0.263	0.343	0.481	0.301	0.228	0.079	0.134	0.057	0.017	0.006
	Mixed	1.851	0.967	0.290	0.408	0.483	0.299	0.237	0.084	0.142	0.059	0.032	0.009
$\pi = 0.4$	Null	1.287	0.660	0.197	0.251	0.261	0.159	0.155	0.054	0.082	0.033	0.010	0.005
	Linear	1.312	0.637	0.259	0.181	0.273	0.152	0.158	0.057	0.192	0.050	0.183	0.138
	Quad	1.309	0.634	0.215	0.251	0.264	0.157	0.155	0.053	0.089	0.030	0.036	0.012
	Trig	1.293	0.647	0.203	0.263	0.261	0.159	0.156	0.054	0.084	0.033	0.013	0.005
	Exp	1.317	0.651	0.186	0.240	0.264	0.156	0.156	0.053	0.084	0.033	0.011	0.004
	Log	1.285	0.650	0.200	0.253	0.262	0.159	0.154	0.051	0.080	0.032	0.013	0.004
	Mixed	1.279	0.664	0.202	0.246	0.267	0.155	0.153	0.051	0.087	0.032	0.033	0.008
$\pi = 0.6$	Null	1.084	0.516	0.165	0.208	0.178	0.106	0.126	0.042	0.064	0.025	0.009	0.004
	Linear	1.124	0.533	0.224	0.136	0.196	0.098	0.126	0.044	0.177	0.044	0.178	0.139
	Quad	1.063	0.523	0.186	0.212	0.184	0.101	0.127	0.043	0.076	0.024	0.043	0.009
	Trig	1.049	0.508	0.173	0.213	0.180	0.104	0.126	0.043	0.064	0.025	0.010	0.004
	Exp	1.052	0.510	0.159	0.203	0.187	0.102	0.126	0.042	0.064	0.025	0.011	0.006
	Log	1.063	0.544	0.176	0.216	0.179	0.106	0.125	0.042	0.063	0.026	0.011	0.003
	Mixed	1.055	0.549	0.183	0.217	0.193	0.099	0.125	0.042	0.074	0.025	0.039	0.009
$\pi = 0.8$	Null	0.948	0.462	0.139	0.173	0.137	0.080	0.109	0.037	0.055	0.023	0.008	0.003
	Linear	0.934	0.444	0.216	0.123	0.159	0.071	0.107	0.034	0.172	0.044	0.189	0.163
	Quad	0.932	0.458	0.171	0.183	0.146	0.074	0.109	0.036	0.073	0.022	0.049	0.009
	Trig	0.935	0.463	0.154	0.204	0.140	0.077	0.110	0.035	0.055	0.023	0.009	0.003
	Exp	0.916	0.469	0.144	0.173	0.152	0.075	0.109	0.035	0.057	0.022	0.013	0.007
	Log	0.885	0.457	0.148	0.186	0.138	0.079	0.107	0.036	0.054	0.022	0.010	0.003
	Mixed	0.922	0.463	0.159	0.173	0.160	0.072	0.109	0.034	0.071	0.022	0.046	0.010

4. Computational time of MACFIV and other methods

Table S14 summarizes the computational time of the proposed method (MACFIV) and other competing methods (TSP, TSP-SCAD, DeepIV, PolyMR, CF) in representative simulation scenarios.

Table S14: Comparison of computational time (in seconds) across different methods.

(n, p, s)	TSP	TSP-SCAD	DeepIV	PolyMR	CF	MACFIV
(200,100,20)	0.009	0.040	0.633	0.005	0.002	0.441
(500,100,20)	0.020	0.078	1.424	0.008	0.005	0.617
(1000,100,20)	0.037	0.164	2.544	0.019	0.011	0.993
(2000,50,20)	0.025	0.342	9.190	0.013	0.007	0.564
(2000,100,0)	0.071	0.364	10.514	0.031	0.014	1.628
(2000,100,10)	0.087	0.337	10.240	0.039	0.015	1.988
(2000,100,20)	0.072	0.365	10.632	0.035	0.015	1.637
(2000,100,40)	0.071	0.574	10.985	0.028	0.013	1.678
(2000,150,20)	0.157	0.483	10.247	0.060	0.029	4.702
(2000,200,20)	0.276	0.641	10.813	0.094	0.045	8.216
(10000,100,20)	3.411	8.380	66.333	0.417	0.210	21.398

Note: The above results are based on a simulation using the nonlinear relationship $f(x) = \sin(0.1 \cdot x)$ as an example. 1000 replications are performed and means of runtime are recorded. The computing environment is 16 CPU cores in one computer node.

5. Sensitivity analysis of MACFIV to initial instrument ordering in the first stage

The first stage of the MACFIV framework constructs a sequence of nested models by ordering the instrumental variables according to their marginal correlations with the exposure. While this ordering is primarily a practical device for generating a coherent model sequence, concerns may arise that, under the many weak instruments setting, sampling variability could render the ranking unstable and potentially affect the final estimation results. To evaluate the robustness of MACFIV to this aspect of the procedure, we conduct a targeted sensitivity analysis.

We consider a representative nonlinear simulation scenario where the true causal function is $f(x) = \sin(0.1 \cdot x)$, the sample size is $n = 2000$, the number of instruments is $p = 50$, and the number of pleiotropy instruments is $s = 20$. Three ordering strategies are compared:

1. Correlation-based ordering (baseline): the default ordering used in MACFIV, in which instruments are ranked by the absolute value of their marginal correlations with the exposure.
2. No ordering: instruments are kept in their original dataset order.
3. Random ordering: instruments randomly permuted in each simulation replicate.

For each strategy, MACFIV estimates the marginal effect function $f'(x)$, and we compute the root mean squared error (RMSE) and mean absolute error (MAE) over 1000 simulation replicates. The resulting RMSE and MAE distributions are shown in Figure S5.

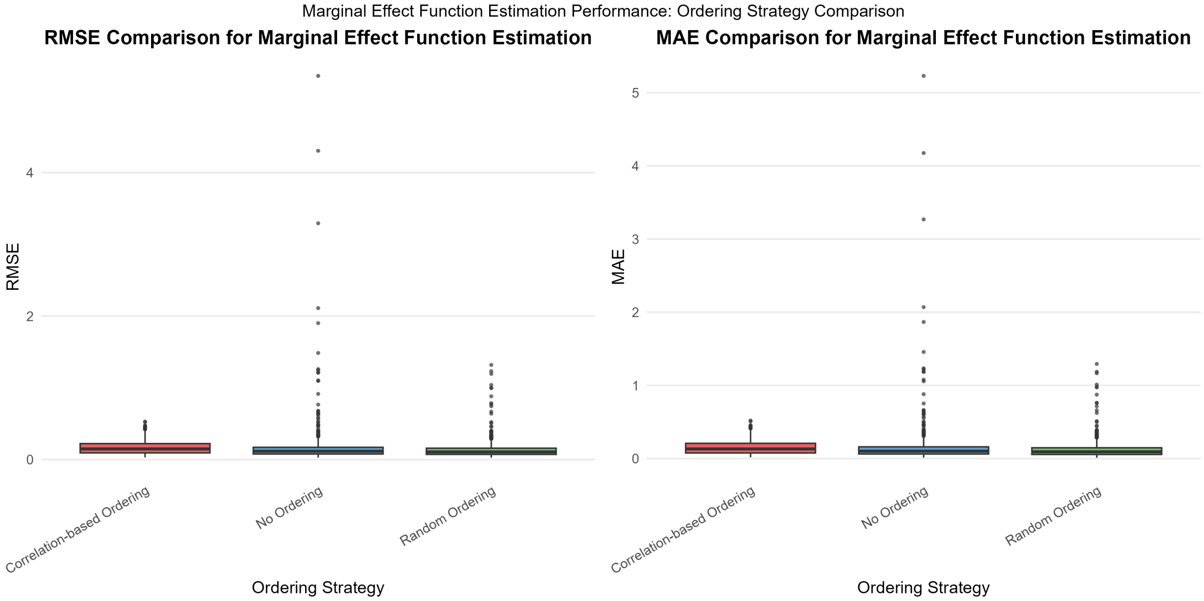

Figure S5: Sensitivity of MACFIV to initial instrument ordering in a representative nonlinear scenario ($f(x) = \sin(0.1 \cdot x), n = 2000, p = 50, s = 20$). Distributions of RMSE and MAE for the marginal effect function estimation $f'(x)$ are shown for correlation-based ordering (baseline, i.e., the default ordering used in MACFIV), no ordering, and random ordering strategies over 1000 simulation replicates.

Alt text: Boxplots showing distributions of root mean squared error and mean absolute error for marginal effect function estimation under different initial instrument ordering strategies, including correlation-based, no ordering, and random ordering.

The results indicate that the RMSE and MAE distributions are highly similar across all three strategies, confirming that MACFIV's performance is largely insensitive to the initial ordering of instruments. We also note that the correlation-based ordering yields slightly fewer extreme outliers, suggesting that informative ordering may offer minor benefits in some settings, although these differences are not systematic.

6. Joint tuning of B-spline number and SCAD parameter

The number of B-spline basis functions (m) and the SCAD tuning parameter λ are two key tuning parameters in MACFIV. While the default implementation fixes m and λ via the Bayesian Information Criterion (BIC) for computational efficiency, it is in principle possible to determine both parameters simultaneously. This joint tuning may offer additional flexibility in adapting to the smoothness of the causal function and the sparsity of pleiotropy effects.

To examine the practical impact of such a strategy, we conduct a simulation study under a representative nonlinear scenario where the true causal function is $f(x) = \sin(0.1 \cdot x)$, the sample size is $n = 2000$, the number of instruments is $p = 50$, and the number of pleiotropy instruments is $s = 20$. We compare:

1. Separate tuning (baseline) – The default approach used in MACFIV: m is fixed at 5 following related literature, and λ is selected via BIC.

2. Joint tuning via cross-validation – m is selected from the candidate set $\{3, 4, 5, 6, 7\}$ and λ is chosen simultaneously from a pre-specified grid using 5-fold cross-validation, selecting the pair with the lowest cross-validation error.

For each approach, MACFIV estimates the marginal effect function $f'(x)$, and we compute the root mean squared error (RMSE) and mean absolute error (MAE) across 1000 simulation replicates. The resulting RMSE and MAE distributions are shown in Figure S6.

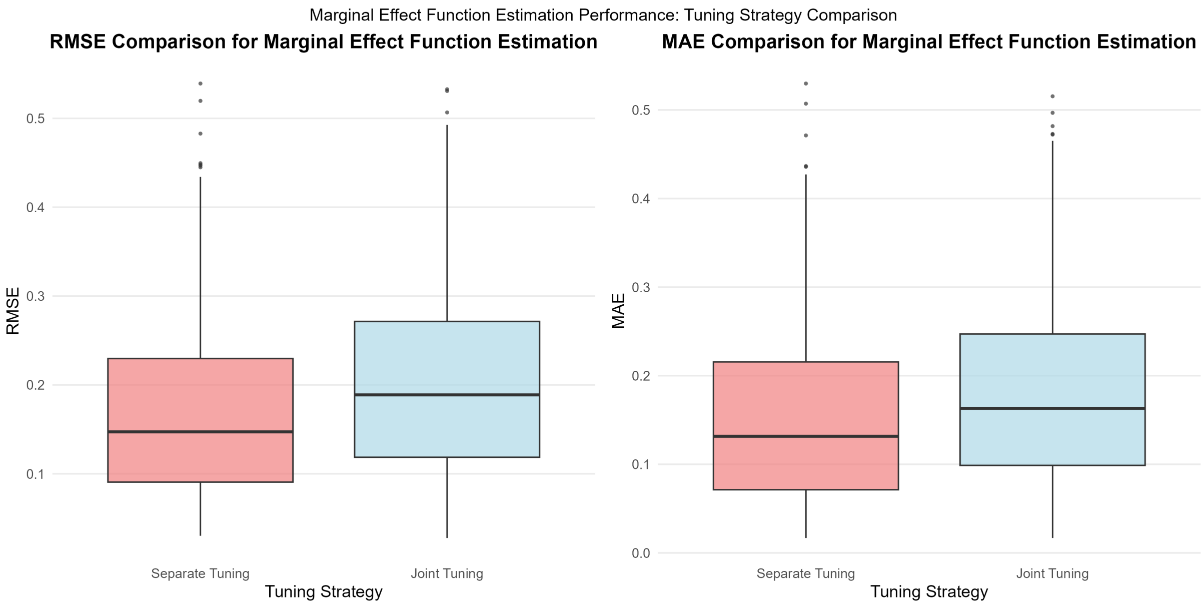

Figure S6: Comparison of MACFIV parameter tuning strategies in a representative nonlinear scenario ($f(x) = \sin(0.1 \cdot x), n = 2000, p = 50, s = 20$). Distributions of RMSE and MAE for the marginal effect function estimation $f'(x)$ are shown for separate tuning (baseline, i.e., fixed $m = 5$ with BIC-selected λ) and joint tuning via 5-fold cross-validation, over 1000 simulation replicates.

Alt text: Boxplots showing distributions of root mean squared error and mean absolute error for marginal effect function estimation under different parameter tuning strategies, comparing separate tuning and joint tuning approaches.

The results show that joint cross-validation yields RMSE and MAE values very close to those of the separate tuning approach, with differences generally within 3%. This suggests that the simpler fixed-m plus BIC-λ procedure used as the default in MACFIV achieves comparable accuracy while being substantially more computationally efficient.

7. Sensitivity of MACFIV to pleiotropic effect magnitude

This analysis examines how the performance of the MACFIV framework varies with the magnitude of pleiotropic effects. We consider a representative nonlinear scenario where the true causal function is $f(x) = \sin(0.1 \cdot x)$, the sample size is $n = 2000$, the number of instruments is $p = 50$, and the number of pleiotropy instruments is $s = 20$ and vary the true pleiotropic effect size across 0.2 (weak), 0.5 (moderate), 1.0 (strong). For each setting, we evaluate:

1. Estimation accuracy of the marginal effect function using root mean squared error (RMSE) and mean absolute error (MAE).
2. Variable selection performance for pleiotropic instruments, measured by false positive rate (FPR) and true positive rate (TPR).

We repeat the simulation 1,000 times for each pleiotropic effect size. The results are summarized in Figure S7.

The results show that RMSE and MAE remain stable across different pleiotropic effect sizes, indicating that estimation accuracy does not materially deteriorate when pleiotropic effects are weak. For variable selection, TPR is consistently close to 1.0, while FPR increases for weak pleiotropic effects due to the difficulty of distinguishing very weak effects from noise. These findings are consistent with the theoretical properties of SCAD, which tends to shrink small coefficients toward zero in sparse settings.

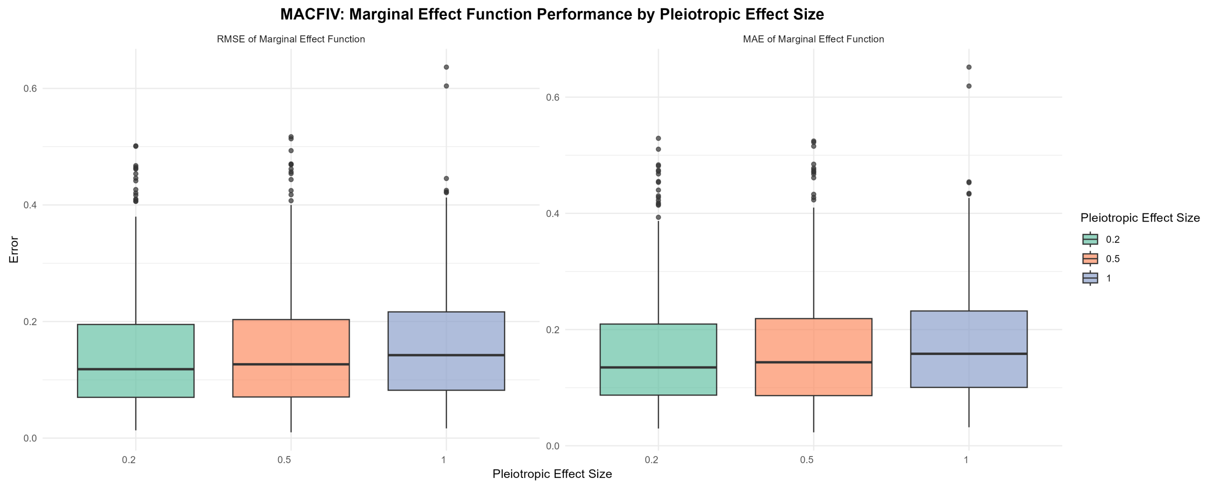

Figure S7: Sensitivity of MACFIV to pleiotropic effect size in a representative nonlinear scenario ($f(x) = \sin(0.1 \cdot x), n = 2000, p = 50, s = 20$), based on 1,000 simulation replicates. Top panels: RMSE and MAE of the estimated marginal effect function. Bottom panels: false positive rate (FPR) and true positive rate (TPR) for identifying pleiotropic instruments.

Alt text: Multi-panel plots showing estimation accuracy and pleiotropic instrument identification performance of the MACFIV method under different pleiotropic effect sizes, including root mean squared error, mean absolute error, false positive rate, and true positive rate.

8. Simulation results for the extended algorithm with nonlinear control functions

To further complement our main analysis, we conduct an additional simulation where the control function takes nonlinear forms. Specifically, instead of assuming $u = \rho v + e$, we consider $u = q(v) + e$ with $q(\cdot)$ following several nonlinear specifications, including cubic, exponential, logarithmic, quadratic, and sinusoidal functions. The estimation procedure follows exactly the same two-step strategy as in the baseline framework, except that the second stage is augmented with a nonparametric approximation of $q(\cdot)$, as mentioned in Section S2.1.

For different specifications of the control function $q(\cdot)$, we use a representative nonlinear causal function where the true causal function is $f(x) = \sin(0.1 \cdot x)$, the sample size is $n = 2000$, the number of instruments is $p = 50$, and the number of pleiotropy instruments is $s = 20$. The instruments are generated with a correlation structure as in the main simulations. Performance is evaluated by computing the root mean squared error (RMSE) and mean absolute error (MAE) of the estimated marginal effect function over 1000 simulation replicates.

Figure S8 summarizes the results. Across all specifications of $q(\cdot)$, the extended algorithm remains stable and produces estimates comparable to those obtained under the linear control function. Moderate nonlinearities, such as quadratic or cubic dependence, result in only minor efficiency loss. More complex nonlinearities, such as sinusoidal dependence, yield somewhat higher variability, but the estimates remain accurate on average. These results demonstrate that the nonlinear control function extension serves as a robust and practical complement to the baseline MACFIV procedure, broadening its applicability while preserving the overall structure and ease of implementation.

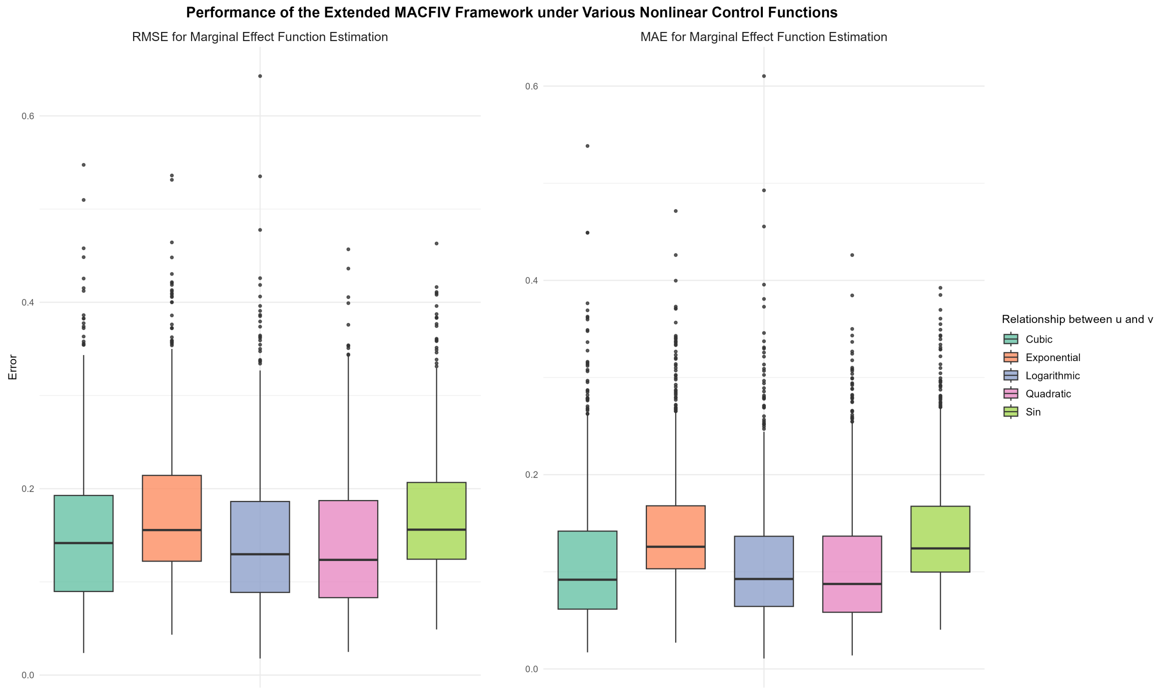

Figure S8: Performance of the extended MACFIV algorithm under nonlinear control functions in a representative scenario $(f(x) = \sin(0.1 \cdot x), n = 2000, p = 50, s = 20)$, based on 1,000 simulation replicates. Distributions of RMSE and MAE for the estimation of the marginal effect function $f'(x)$ are reported across 1,000 simulation replicates under different specifications of the control function $q(\cdot)$.

Alt text: Boxplots showing distributions of root mean squared error and mean absolute error for marginal effect function estimation under different specifications of the control function.

9. Simulation results for the binary outcome extension of MACFIV

To illustrate the performance of the binary outcome extension of MACFIV, we conduct a simulation study following the model described in Section S2.2. We follow exactly the data-generating process described in the Simulation section of the main text, except that the continuous outcome is replaced by a binary response generated via a logistic link. All notation and parameter settings (for $\boldsymbol{G}, \boldsymbol{X}$, and the error components) are the same as in the main simulations. Using the same structural index that defines the continuous outcome, the binary response is generated according to

$$\Pr\left(y_i = 1 \mid x_i, \boldsymbol{g}_i, u_i\right) = \operatorname{logit}^{-1}\left\{f\left(x_i\right) + \boldsymbol{g}_i^{\top}\boldsymbol{\alpha} + u_i\right\},$$

where $f(x)$ is chosen from the same set of nonlinear functions considered in the main simulation. This transformation replaces the continuous outcome $y_i = f(x_i) + \boldsymbol{g_i}^T\boldsymbol{\alpha} + u_i$ in the main simulations with a Bernoulli variable while keeping the underlying structural relation identical.

The binary outcome version of MACFIV, described in Section S2.2, is applied to estimate $f(x), \boldsymbol{\alpha}$ and the endogeneity parameter ρ. To evaluate the binary outcome extension, we focus on the weak-instrument setting and vary the number of invalid instruments at three levels (10, 20, and 40). For each configuration, seven functional forms of $f(x)$ are considered, corresponding to those used in the main simulation study. The sample size is set to $n = 10000$ and the total number of instruments to $p = 100$.

In the framework of interpretable causal modeling, we carefully select comparison methods that are both conceptually aligned with MACFIV-Binary and practically extendable to binary outcomes. Specifically, three benchmark approaches are included:

- **TSP (logistic version)**: a two-stage predictor substitution approach in which the second-stage regression is replaced by a logistic model. Compared with the standard TSP used for continuous outcomes, this version adapts naturally to binary responses while retaining the same substitution-based correction structure.

- **TSP-SCAD**: an extension of the logistic TSP method that adds SCAD penalization in the second-stage logistic regression. This comparison helps isolate the effect of adaptive penalization when applied within the substitution-based framework, contrasting it with the penalized control-function strategy used in MACFIV-Binary.

- **Control Function (CF)**: the most direct competitor to MACFIV-Binary. It also employs

a two-stage approach, where the residual from the first stage is included as an additional covariate in the second-stage logistic regression. Unlike MACFIV-Binary, however, CF does not incorporate model averaging or SCAD-based penalization, which highlights the contribution of these two features in improving estimation stability and interpretability.

Together, these comparisons situate MACFIV-Binary within an interpretable modeling framework, emphasizing how model averaging and adaptive penalization enhance estimation accuracy and robustness beyond standard logistic control-function or substitution-based methods.

Performance is evaluated using the root mean squared error (RMSE), which measures the deviation of the estimated marginal effect from its true value. Each configuration is replicated 500 times, and the averaged RMSE results are summarized in Figure S9, which presents bar plots across the seven nonlinear functions under different proportions of invalid instruments.

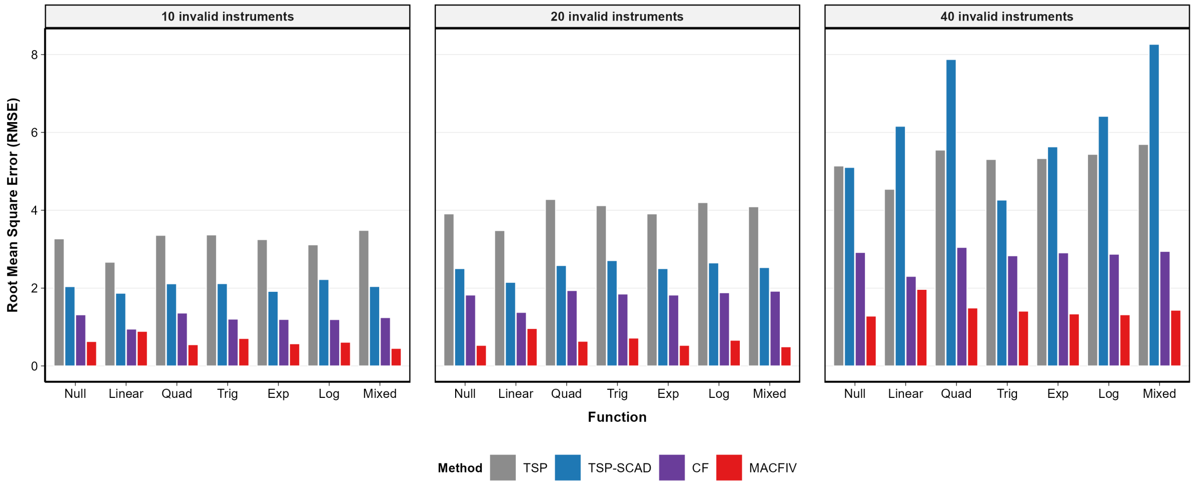

Figure S9: Grouped bar plot of the RMSE of the estimated marginal effect function $f'(x)$ for TSP, TSP-SCAD, CF, and MACFIV methods under the binary outcome setting, across different functional forms ($f(x)$) and numbers of invalid instruments ($s = 10, 20, 40$). The sample size is fixed at $n = 10000$ and the number of instruments at $p = 100$. For each setting, results are averaged over 500 replications.

Alt text: Grouped bar plot showing root mean squared error of marginal effect function estimation for multiple methods under a binary outcome setting, comparing different functional forms and numbers of invalid instruments.

Across all scenarios, MACFIV consistently achieves the lowest RMSE, indicating superior estimation accuracy of the marginal effect function under binary or categorical outcomes. In contrast, the standard TSP and CF methods exhibit substantially larger errors, particularly when the proportion of invalid instruments increases, reflecting their limited robustness to instrument invalidity. While TSP SCAD benefits from adaptive penalization and shows improved performance over TSP, its performance deteriorates markedly as the number of invalid instruments

increases. This degradation is mainly due to the presence of a few unstable estimates under severe invalidity, which inflate the average RMSE we observe. Although MACFIV remains the best-performing method overall, its RMSE also increases slightly as invalid instruments accumulate. Furthermore, under the same simulation settings, the RMSE of the categorical outcome version is somewhat higher than that of the continuous outcome version, suggesting that the estimation becomes more challenging when the response variable is discrete, and further refinement could help improve robustness in such cases. Overall, these results demonstrate that the proposed categorical outcome extension of MACFIV retains its advantages in bias correction and estimation precision, even in challenging weak and partially invalid instrument settings.

10. Robustness checks for the nonlinear BMI–DBP relationship

To verify that the nonlinear pattern estimated by MACFIV is not an artifact of the chosen B-spline specification, we perform robustness checks using alternative smoothing approaches. Specifically, we fit the BMI–DBP curve using B-splines with 4, 5, and 6 knots, and for each knot number, we consider two placement strategies: equally spaced and quantile-based. We also apply kernel smoothing and local polynomial regression as nonparametric alternatives.

Figure S10 shows the fitted curves and their corresponding turning points. Across all methods, the estimated turning points fell within a narrow range ($31.76 \sim 34.34$ kg/m^2), with the default MACFIV setting ($m = 5$, uniform knots) yielding 33.41 kg/m^2. This consistency suggests that the turning point is not an artifact of a specific smoothing specification but rather a robust feature of the data.

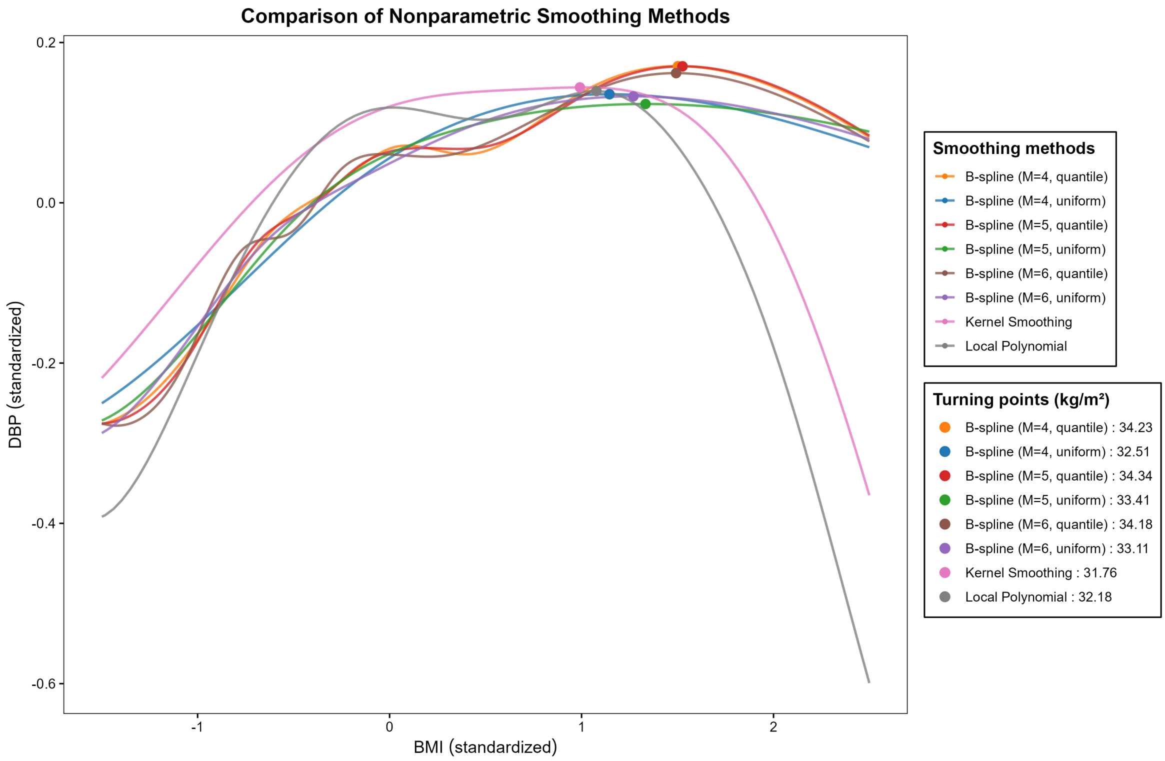

Figure S10: Robustness of the estimated turning point in the BMI–DBP relationship to different smoothing specifications. Curves are estimated using B-splines with varying numbers of basis functions ($m \in \{4, 5, 6\}$) and both quantile-based and evenly spaced knots, as well as kernel smoothing and local polynomial regression.

Alt text: Smoothed curves showing the estimated relationship between body mass index and diastolic blood pressure under different smoothing specifications, illustrating the robustness of the estimated turning point.

References

[1] Fan Q, Guo Z, Mei Z, Zhang CH. Inference for nonlinear endogenous treatment effects accounting for high-dimensional covariate complexity. arXiv preprint arXiv:231008063. 2024.

[2] Wang X, Huang T, Jia J. Non-linear Mendelian randomization with two-stage prediction estimation and control function estimation. arXiv preprint arXiv:240201121. 2024.

[3] Elliott G, Gargano A, Timmermann A. Complete subset regressions. Journal of Econometrics. 2013;177(2):357-73.

[4] Leeb H, Pötscher BM. Model selection and inference: Facts and fiction. Econometric Theory. 2005;21(1):21-59.

[5] Liu CA. Distribution theory of the least squares averaging estimator. Journal of Econometrics. 2015;186(1):142-59.

[6] De Boor C. A practical guide to splines. vol. 27. Springer-Verlag New York; 1978.

[7] Zhou S, Wolfe DA. On derivative estimation in spline regression. Statistica Sinica. 2000;10:93-108.

[8] Fan J, Li R. Variable selection via nonconcave penalized likelihood and its oracle properties. Journal of the American Statistical Association. 2001;96(456):1348-60.
